# Chirality Makes or Breaks Chemically Driven Self‐Assembly

**DOI:** 10.1002/anie.202508481

**Published:** 2025-07-31

**Authors:** Lenard Saile, Kun Dai, Mahesh D. Pol, Thejus Pramod, Ralf Thomann, Charalampos G. Pappas

**Affiliations:** ^1^ Cluster of Excellence *liv*MatS @FIT – Freiburg Center for Interactive Materials and Bioinspired Technologies University of Freiburg Georges‐Köhler‐Allee 105 79110 Freiburg Germany; ^2^ Institute of Organic Chemistry University of Freiburg Albertstrasse 21 79104 Freiburg Germany; ^3^ Freiburg Materials Research Center (FMF) University of Freiburg Stefan‐Meier‐Strasse 21 79104 Freiburg Germany

**Keywords:** Acylation, Aminoacyl phosphates, Chirality, Non‐equilibrium self‐assembly, Peptides

## Abstract

Nature has consistently selected homochiral building blocks from millions of possible diastereomers across diverse biomolecular structures to drive molecular recognition, catalysis and self‐assembly. Despite its central role in biology, chirality's influence on chemically driven reaction networks remains unexplored. Here, we demonstrate that chiral aminoacyl phosphate esters, synthetic analogs of biological acylating intermediates, drive self‐assembly and reaction pathways, that are modulated purely by their configuration, without the need for changes in functional groups. Using enantiopure aminoacyl phosphate esters, we show that these left‐ and right‐handed acylating agents generate transient epimeric (thio)‐esters from homochiral peptide substrates, leading to supramolecular architectures with distinct lifetimes and self‐assembly dynamics. Moreover, chirality regulates downstream reactivity in cascade reactions, where stereochemical control over an intermediate propagates into subsequent transformations. Finally, chiral acylating agents differentiate between two reaction cycles, selectively modulating one pathway while keeping another invariant – a level of control that remains difficult to achieve with conventional chemical strategies. Stereochemical programming enables control over reactivity and self‐assembly, offering new opportunities to encode chirality in reaction networks and modulate their function through a single molecular parameter.

## Graphical abstract

**Chirality‐controlled reaction cycles**: Reaction cycles are constructed to respond to the chirality (handedness) of an acylating agent. Left‐ and right‐handed aminoacyl phosphate esters modulate the structure, lifetime, and transformation of transient (thio)ester intermediates, enabling control over distinct chemical pathways using molecules that are otherwise chemically identical.

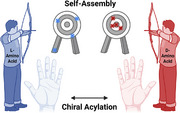

## Introduction

The ability of biomolecules like proteins and sugars to self‐assemble is fundamental to life, governing cellular architecture, enzymatic function and molecular recognition. A defining characteristic of these biopolymers is their exclusive construction from chiral monomers. Remarkably, out of millions of possible heterochiral diastereomers, biosynthesis selects only one homochiral enantiomer.^[^ ^1^, ^2^, ^3^, ^4^, ^5^ ^]^ This stereochemical uniformity ensures highly specific molecular interactions, enabling the formation of functional supramolecular architectures with well‐defined structures and functions. Even minor disruptions to this homochiral preference can have profound consequences,^[^ ^6^ ^]^ altering folding,^[^ ^7^, ^8^ ^]^ binding affinities^[^ ^9^ ^]^ and functional characteristics.^[^ ^10^, ^11^ ^]^ Chirality dictates the self‐organization of biological structures at multiple levels. In proteins and peptides, homochirality underpins enzymatic catalysis, ensuring selective substrate binding and reaction control.^[^ ^12^, ^13^, ^14^ ^]^ In nucleic acids, the chirality of the sugar‐phosphate backbone determines the helical handedness of DNA,^[^ ^15^ ^]^ influencing genetic storage, replication and transcription. At the supramolecular level,^[^ ^16^ ^]^ stereochemical effects play a crucial role in phase separation^[^ ^17^, ^18^ ^]^ and amyloid fibril formation,^[^ ^19^ ^]^ where deviations from homochirality can disrupt aggregation pathways. These examples illustrate how chirality extends beyond molecular recognition to actively regulate higher‐order biological functions.

While the self‐assembly of homo‐ and heterochiral peptides has been widely explored under equilibrium conditions,^[^ ^20^, ^21^, ^22^, ^23^, ^24^, ^25^ ^]^ biological self‐assembly rarely occurs under static conditions. Instead, living systems rely on continuous energy input to drive dynamic structural transformations, leading to assemblies that form, dissipate and reorganize over time.^[^ ^26^, ^27^, ^28^ ^]^ The integration of stereochemical information with chemically driven assembly is a hallmark of biological organization. A striking example is the self‐assembly of cytoskeletal proteins such as actin^[^ ^29^ ^]^ and tubulin,^[^ ^30^ ^]^ which rely on chiral monomers to regulate polymerization and depolymerization dynamics. These highly dynamic architectures are powered by adenosine triphosphate (ATP), enabling precise control over intracellular transport, cell division and motility through cycles of growth and disassembly. Additionally, achiral activation mechanisms, such as palmitoylation,^[^ ^31^ ^]^ acetylation and phosphorylation^[^ ^32^ ^]^ also depend on stereochemical properties of their chiral substrates during folding or self‐assembly. These examples highlight the fundamental role of chirality in active self‐assembly, where stereochemical effects influence reaction kinetics and macroscopic function.

Inspired by natural systems, chemists have developed synthetic strategies to regulate self‐assembly in space and time,^[^ ^33^, ^34^, ^35^, ^36^, ^37^, ^38^ ^]^ employing reaction cycles driven by carbodiimides,^[^ ^39^ ^]^ methylating agents,^[^ ^40^ ^]^ Michael acceptors,^[^ ^41^ ^]^ anhydrides,^[^ ^42^ ^]^ triphosphates,^[^ ^43^, ^44^, ^45^, ^46^ ^]^ disulfides^[^ ^47^ ^]^ and activated acids,^[^ ^48^ ^]^ but also physical stimuli like light^[^ ^49^ ^]^ and electricity.^[^ ^50^ ^]^ These dynamic processes enable the temporal control of self‐assembled structures, mimicking the transient nature of biological assemblies. Despite these advances, the structural characteristics of the activating species have rarely been leveraged to dictate the properties of the assembled state. In most abiotic systems, activating agents either remain excluded from the transiently assembling structure or influence self‐assembly only indirectly. This behavior contrasts with biological systems, where the chemical structure of the activating agents plays an important role in governing transient structural transitions. Particularly, the role of chirality in chemically driven self‐assembly remains underexplored. Understanding how both molecular structure and stereochemical effects influence self‐assembly dynamics^[^ ^51^, ^52^ ^]^ could provide important insights into biological organization and inspire new strategies for designing adaptive synthetic materials. In molecular machines,^[^ ^53^ ^]^ for example, chiral carbodiimides have been used to induce directionality in rotational motion, demonstrating how stereochemical information can be embedded into non‐equilibrium processes.^[^ ^54^, ^55^ ^]^

Recently, (amino)acyl phosphate esters^[^ ^56^ ^]^ have emerged as promising acylating agents^[^ ^57^, ^58^ ^]^ as well as educts^[^ ^59^, ^60^ ^]^ and products^[^ ^42^, ^61^ ^]^ of chemically driven reaction cycles. These compounds exhibit a striking structural resemblance to key biochemical high‐energy intermediates,^[^ ^62^ ^]^ such as aminoacyl adenylates in peptide biosynthesis,^[^ ^63^ ^]^ acyl adenylates in Acetyl‐CoA synthesis and acyl phosphates in glycolysis.^[^ ^64^ ^]^ Their relevance extends beyond biological analogues, as they have also been proposed to play a role in prebiotic chemistry, where they may have contributed to early peptide formation and the emergence of selective self‐assembly.^[^ ^65^, ^66^, ^67^, ^68^ ^]^ These attributes make aminoacyl phosphates particularly compelling candidates for designing abiotic systems that harness chirality to control non‐equilibrium self‐assembly. Our findings reveal a direct connection between chirality and non‐equilibrium self‐assembly, enabling the design of stereochemically programmed reaction cycles with controllable lifetimes, responsiveness and dynamic structural evolution.

Here, we introduce a novel acylating strategy in which chirality, a fundamental aspect of molecular recognition, directly governs chemically driven self‐assembly. Utilizing aminoacyl phosphate esters to drive acyl transfer reactions under non‐equilibrium conditions, we demonstrate that left‐ and right‐handed amino acid‐derived acylating agents induce selective self‐assembly pathways. Acylation of homochiral peptide substrates with either L‐ or D‐configured acylating agents yields diastereomeric products that form supramolecular architectures with distinct differences in self‐assembly, lifetime, reactivity and mechanical strength depending on the acylating agent's configuration. Notably, we show that the stereochemistry of the acylating agent influences both the kinetic stability and morphological evolution of the assembled structures, enabling control over transient states and reaction‐driven transformations. We further demonstrate that chirality influences reaction cascades, where stereochemical control over an intermediate directs downstream transformations. Finally, chiral acylating agents selectively modulate one reaction cycle while keeping another invariant, enabling a level of control that is challenging to achieve with conventional approaches.

## Results and Discussion

### Integrating Chiral Information in an Acylating Agent

**Figure 1:**
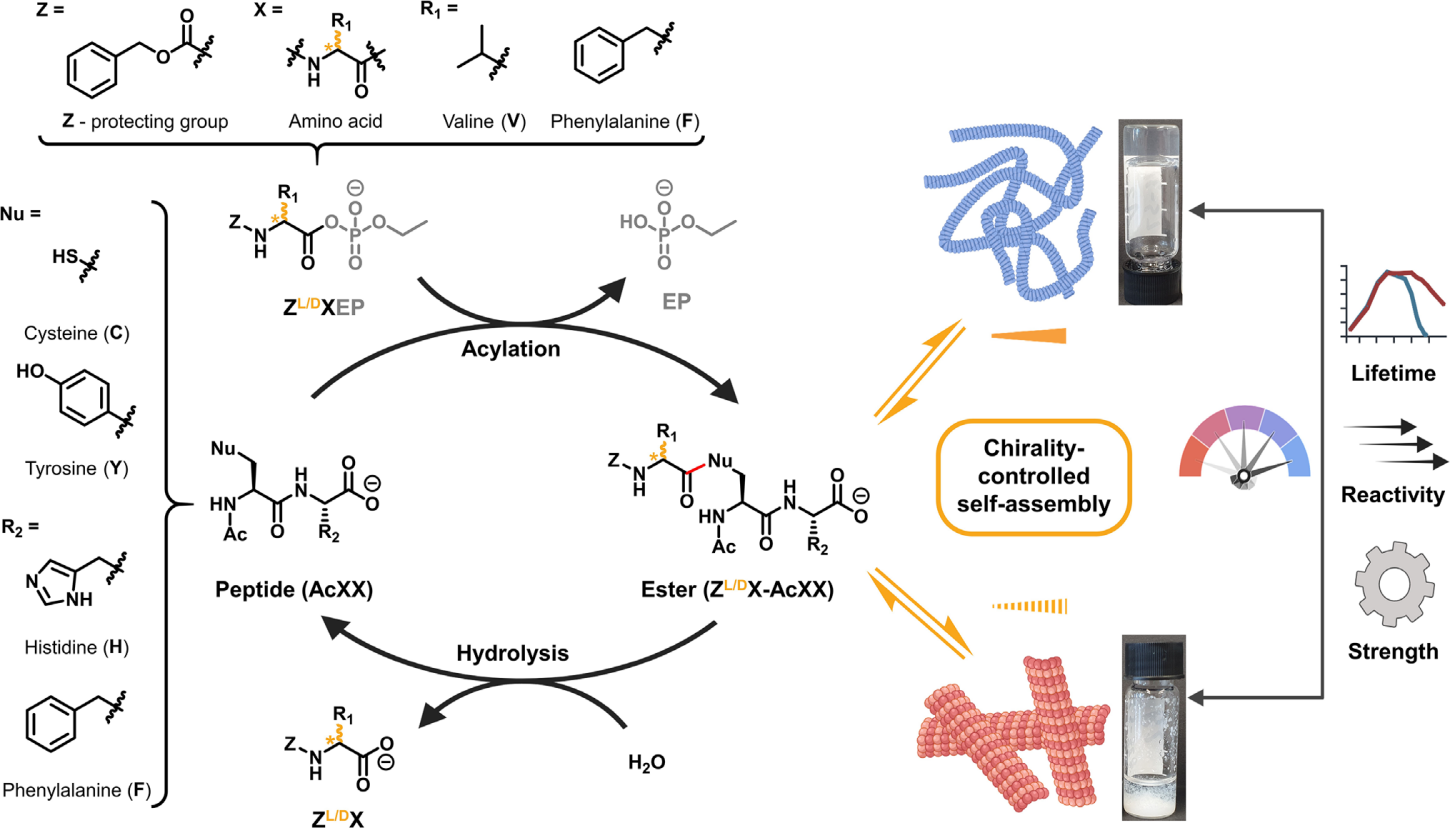
Schematic representation of the reaction cycle driven by chiral aminoacyl phosphate esters (**Z^L/D^XEP**), leading to transient (thio)ester formation and subsequent hydrolysis. The stereochemistry of the acylating agent dictates self‐assembly, resulting in supramolecular architectures with distinct lifetimes, reactivity and mechanical properties. Representative images illustrate the impact of chirality on assembly behavior. The molecular structures of amino acids (X), aminoacyl phosphate esters (**Z^L/D^XEP**) and peptide substrates (**AcXX**) are shown on the left side of the figure.

We previously introduced a chemically driven self‐assembly system in which high‐energy acylating species incorporate structural elements into activated states within a reaction cycle.^[^ ^59^ ^]^ This approach allowed us to modulate the properties of self‐assembled structures by tailoring the acylating agent's design. Here, we extend this concept by utilizing the chirality of the aminoacyl phosphates as central motifs, enabling precise control over assembly and reactivity. Within these structures, one key variable is the N‐terminus, which can be left unprotected to facilitate amino acid oligomerization in multicomponent reaction networks.^[^ ^58^ ^]^ However, in the simpler reactions examined here, we employed Z‐protected (Cbz‐protected) amino acids to suppress oligomerization while introducing an aromatic ring to promote π‐stacking interactions in the assembled state. Importantly, the amino acid side chain serves as the primary structural element: by incorporating natural and non‐natural amino acids, we modulated both nanostructure formation and assembly lifetime,^[^ ^59^ ^]^ as well as reactivity in cascade transformations.^[^ ^60^ ^]^ Additionally, the high polarity of the phosphate activation group allows hydrophobic side chains to remain soluble in aqueous conditions without requiring organic co‐solvents. In this study, both L‐ and D‐amino acid derivatives were separately phosphorylated using ethyl phosphate (EP) (Figure 1), generating enantiomeric acylating agents following previously reported methods.^[^ ^42^ ^]^ The notation ^L/D^ and L‐ or D‐driven indicates that either the L‐ or D‐configured acylating agents were used. Throughout this manuscript, we use single‐letter amino acid codes, with **X** denoting a variable amino acid, as depicted in Figure 1. Under alkaline conditions, these acyl phosphates (**Z^L/D^XEP**, Figure 1) undergo acyl transfer reactions with nucleophilic peptide side chains. Cysteine (C) and tyrosine (Y) peptides were chosen as nucleophilic substrates due to their ability to form metastable (thio)esters (**Z^L/D^X‐AcXX**). These intermediates spontaneously hydrolyze under aqueous alkaline conditions, regenerating the peptide substrate (**AcXX**) and producing the deactivated amino acid derivative (**Z^L/D^X**). The high nucleophilicity of cysteine enabled efficient thioester formation at pH 9.1, whereas tyrosine, with a higher pKa (∼10.1),^[^ ^69^ ^]^ required deprotonation to phenolate at pH 10.1 for sufficient reactivity. To investigate how amino acid side chains influence reactivity, we compared phenylalanine (F) and valine (V) as model residues. Phenylalanine was selected for its π‐stacking capability, while valine, with its aliphatic structure, was expected to engage in hydrophobic interactions and exhibit reduced reactivity. The lower reactivity of aliphatic acyl phosphates relative to aromatics was hypothesized to arise from steric hindrance and electronic inductive effects of the aromatic ring.^[^ ^60^ ^]^ These reactivity differences are also reflected in different hydrolysis rates (Figure S45). We then examined how chirality affects the behavior of reaction cycles by adding either L‐ or D‐acyl phosphate esters to L‐peptide substrates, maintaining the natural homochirality of peptide backbones. The lifetime of the assembled (thio)ester intermediates was measured via high‐performance liquid chromatography (HPLC), while the resulting self‐assembled structures were characterized using confocal fluorescence and transmission electron microscopy. Additionally, we assessed their mechanical and optical properties in the activated state to establish the relation between reaction kinetics and self‐assembly behavior.

### Chirality Transiently Modulates Self‐Assembly

**Figure 2:**
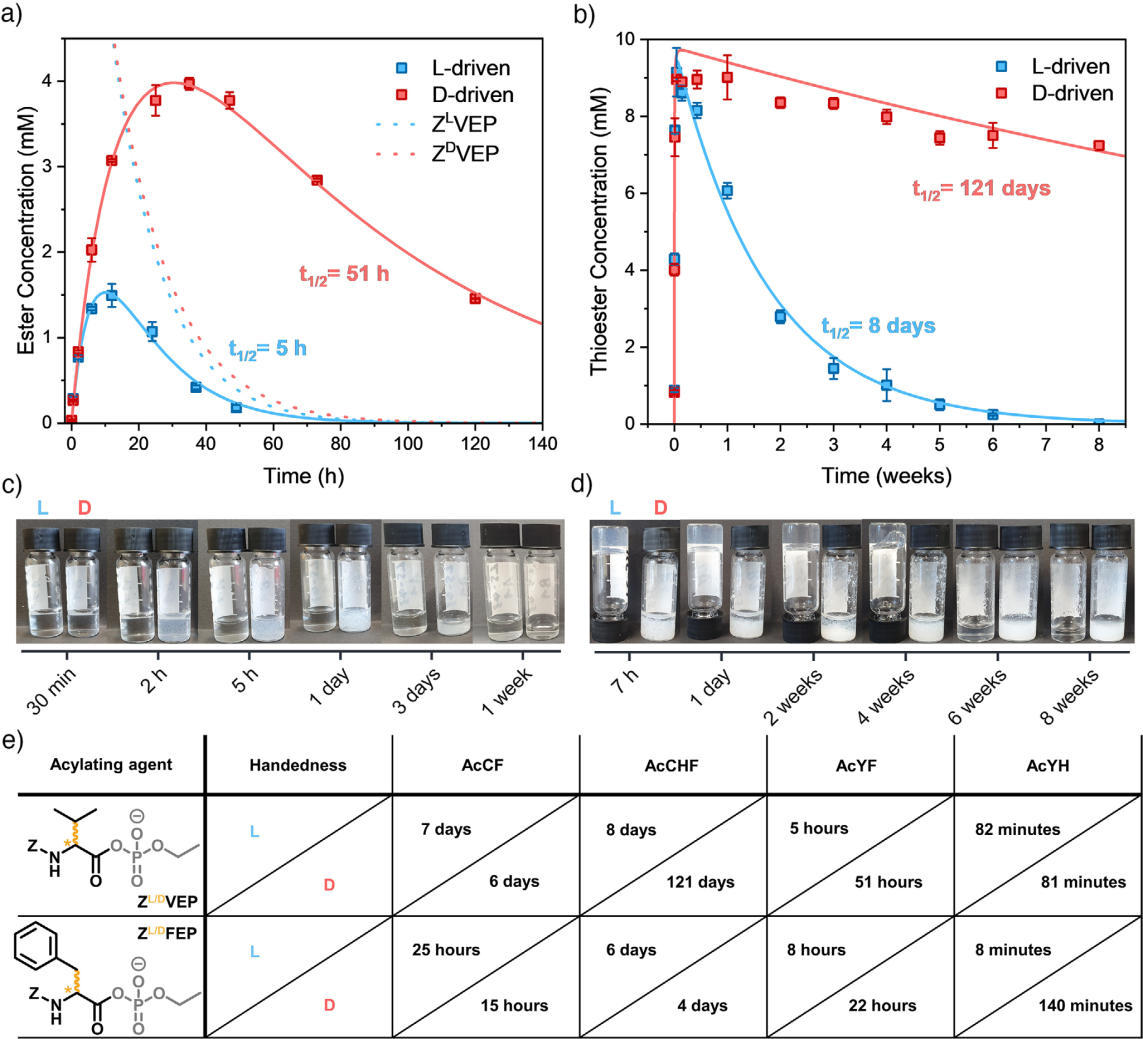
a) Ester concentration over time and c) images of the reaction vials from the reaction of **Z^L/D^VEP** (10 mM) with **AcYF** (20 mM) in bicarbonate buffer. The dotted lines represent the concentration of the enantiomeric acylating agents. b) Thioester concentration over time and d) images of the reaction vials from the reaction of **Z^L/D^VEP** (10 mM) with **AcCHF** (10 mM) in borate buffer. e) (Thio)ester half‐lifetimes from fitting (thio)ester concentration data to a first‐order exponential decay using a kinetic model (Table S1). Reactions with cysteine (C) peptides were carried out in borate buffer (0.6 M, pH 9.1) with one equivalent of peptide and reactions with tyrosine (Y) peptides were carried out in bicarbonate buffer (0.2 M, pH 10.1) with two equivalents of peptide. Error bars represent the standard deviation of three independent experiments.

**Figure 3:**
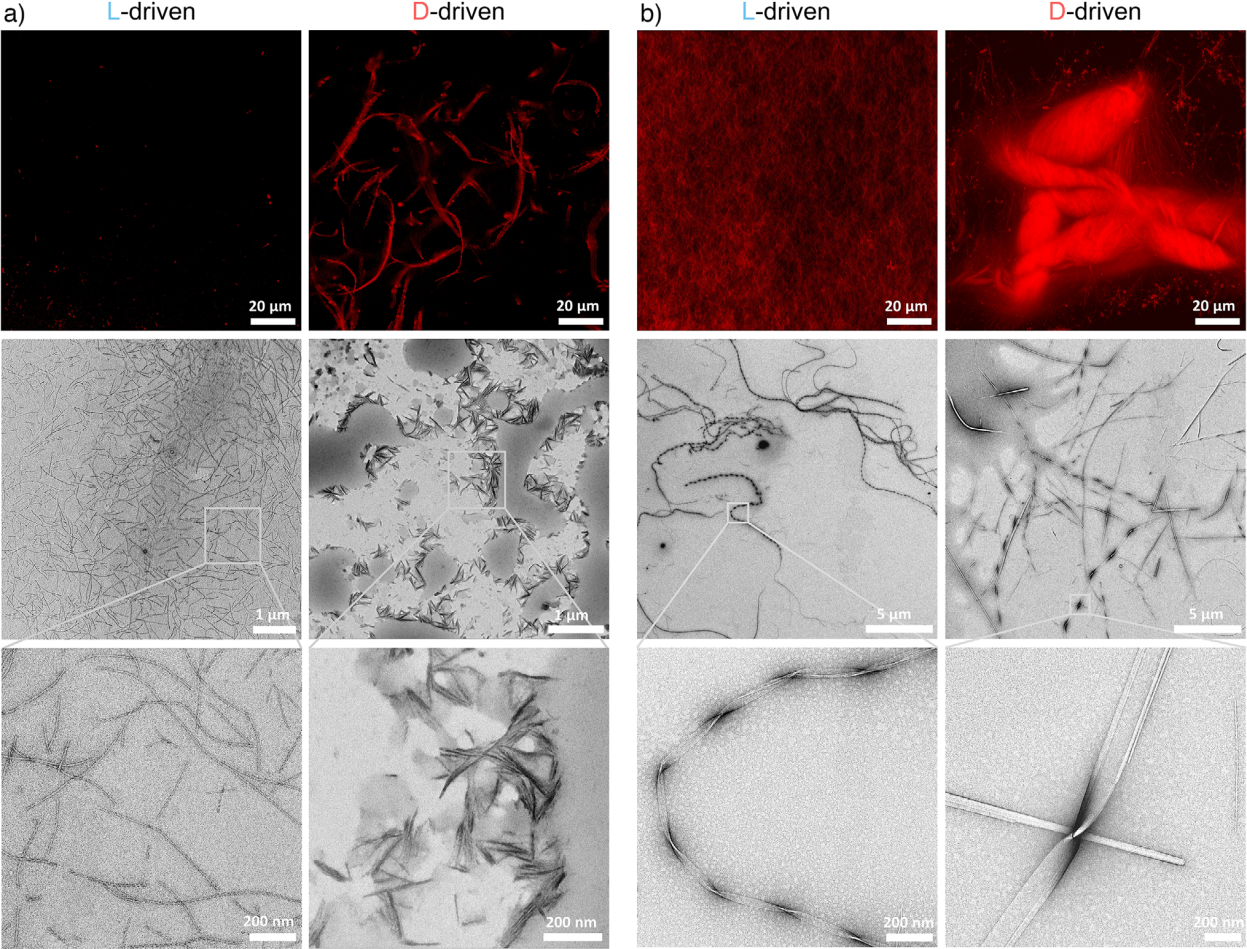
Confocal images (first row) and TEM images (second and third row) for a) the reaction of **Z^L/D^VEP** (10 mM) with **AcYF** (20 mM) in bicarbonate buffer after one day and b) the reaction of **Z^L/D^VEP** (10 mM) with **AcCHF** (10 mM) in borate buffer after 3 days. Nile Red was used as fluorescent dye for the confocal images and uranyl acetate was used to stain TEM samples.

We started by synthesizing each left and right‐handed acyl phosphate ester from commercially available enantiopure Z‐protected amino acids using literature reported methods.^[^ ^42^ ^]^ The enantiomeric purity of the hydrolysis products was confirmed via HPLC on a chiral stationary phase, yielding an enantiomeric excess (ee) of >98% (Figures S1 and S2). An overview of chemical structures and corresponding names can be found in Figures S3 and S4. In order to investigate the influence of the handedness of the acylating agent on the reaction cycle, we examined the system with a range of acylating agents and peptide combinations. We selected peptide substrates with either cysteine (C) or tyrosine (Y) at the first position as the nucleophilic site for acyl transfer. The peptide substrate was acetylated to suppress amide bond formation. Previous studies in related systems^[^ ^59^ ^]^ demonstrated that ester hydrolysis occurred faster in solution than in the assembled phase. Consequently, differences in transient diastereomer lifetimes could arise via three potential mechanisms (Figure S5), where the diastereomeric (thio)esters hydrolyze: 1) with different rates in solution (k_hydrolysis, solution, L_ ≠ k_hydrolysis, solution, D_), 2) with similar rates in solution and similar rates in the assembled state but the equilibrium between (thio)esters in solution and in the assembled state is shifted (K_eq, L_ ≠ K_eq, D_) or 3) with different rates in the assembled state (k_hydrolysis, assembly, L_ ≠ k_hydrolysis, assembly, D_). From different combinations of acylating agents and peptide substrates that produce a soluble diastereomer, we have not observed differences in their lifetime (**Z^L/D^VEP** and **AcYH**, **Z^L/D^FEP** and **AcYD**), which rules out mechanism 1 as a significant factor. For simplicity, we focused on short peptides (up to tripeptides) and restricted our study to conditions where self‐assembly occurs only after acylation. In order to induce self‐assembly after acylation by the aminoacyl phosphate esters, the peptide needs to be able to engage efficiently in intermolecular interactions in the acylated state. Thus, we used phenylalanine at the second position of the peptide to induce pi‐pi‐stacking and hydrophobic interactions (**AcYF**). If one diastereomer is integrated more effectively into the assembled state, differences in lifetime would likely originate from a shifted equilibrium between soluble and assembled states (mechanism 2). Indeed, distinct behavior was observed when **AcYF** (20 mM) was acylated with either the L‐ or D‐configured acyl phosphate **Z^L/D^VEP** (10 mM) in bicarbonate buffer (0.2 M, pH 10.1). The L‐driven system remained in solution, while the D‐driven system formed a precipitate after 2 h (Figure 2c). This macroscopic difference correlated directly with deactivation kinetics with hydrolysis rates changing by an order of magnitude (Figure 2a, Table S1). Kinetic differences were quantified by building a reaction model and fitting it to experimental data from HPLC measurements using COPASI^[^ ^70^ ^]^ (Table S1). LC‐MS analysis (Figures S54–S89), calibration curves (Figures S46–S53), images of reaction vials (Figures S21–S28) and concentration plots (Figures S29–S37) for each reaction can be found in the Supporting Information. Reaction cycles were compared using the half‐lifetime (t_1/2_) of a first‐order reaction as a comparable parameter for (thio)ester hydrolysis rates (Figure 2e). The L‐driven system had a half‐lifetime of 5 h, whereas the D‐driven system, which formed a precipitate, had a half‐lifetime of 51 h. To better understand the structural basis of these differences, we performed confocal fluorescence microscopy, rheology, circular dichroism (CD) and transmission electron microscopy (TEM). Confocal images using Nile Red staining (Figure 3a), confirmed that the L‐driven system did not form large assemblies, while the D‐driven system exhibited pronounced aggregation. TEM images further revealed that D‐driven assemblies were denser and structurally more compact, supporting the hypothesis that acylation‐induced aggregation impacts hydrolysis kinetics. Additional insights into assembly properties were obtained through rheological measurements, which indicated that the D‐driven system transiently increased in mechanical strength, whereas the L‐driven system remained in solution (Figure S13a). To complement these findings, Nile Red fluorescence was used as a probe for the existence of hydrophobic environments. Nile Red exhibits low fluorescence in polar conditions but increases in intensity in apolar environments, making it a reliable marker for hydrophobic assembly formation.^[^ ^71^ ^]^ The fluorescence intensity over time closely matched the trends observed in HPLC and microscopy: both enantiomers induced a transient fluorescence intensity increase; however, the L‐driven system only reached 20% of the maximum intensity compared to the D‐driven system after 1 day (Figure S9a). In the CD spectra, both systems display pronounced signals at 200 and 230 nm, indicative of beta‐turn assemblies.^[^ ^22^ ^]^ The overall stronger signals observed in the D‐driven system suggest a more defined supramolecular environment, likely due to enhanced organization of aromatic residues within the assembled state (Figure S14a). Next, we applied non‐enantiopure mixtures of the acylating agents to determine whether intermediate lifetimes of the activated state could be accessed between those of the enantiopure systems. Varying the L:D ratio allowed precise control over the lifetime of the transient ester. Specifically, a 100:0 L:D ratio resulted in a half‐lifetime of 5 h, which progressively increased to 8 h (75:25), 12 h (50:50), 25 h (25:75), and up to 51 h for the 0:100 D‐enantiomer (Figures S20 and S29, Table S2).

To explore whether the chirality‐dependent effects observed with valine (V) extend to more reactive aminoacyl phosphates, we substituted valine with phenylalanine (F) in the acylating agent. The trend remained unchanged when **AcYF** (20 mM) was acylated with **Z^L/D^FEP** (10 mM) in bicarbonate buffer: the half‐lifetime of the L‐driven system was significantly shorter (8 h) compared to the D‐driven system (22 h) (Figures 2e, S34). Macroscopically, both systems initially formed opaque, strong gels, however the L‐driven reaction turned into a solution after one day while the D‐driven reaction formed an aggregate that disappeared after one week (Figure S27). Under the confocal microscope, the L‐driven system showed unresolved small structures that were found to be centered clusters of nanotapes of around 1 µm size from TEM (Figure S15). On the other hand, the D‐driven system aggregated into larger fibers visible from the confocal microscope and identified as nanotape‐like assemblies that clustered into elongated structures on the µm scale (Figure S15). Rheology data indicated that both gels initially exhibited similar mechanical strength, but the L‐driven gel rapidly lost its mechanical strength after one day, whereas the D‐driven gel remained intact for three days (Figure S13b).

Next, we examined a cysteine (C)‐containing peptide substrate, **AcCF**, to evaluate whether chirality‐dependent effects were consistent with those observed in tyrosine‐containing systems. There were no significant differences observed in (thio)ester lifetime (Figures 2e, S30 and S31). However, despite similar kinetics, distinct macroscopic and microscopic differences were noticed (Figures S21 and S24). When **Z^L^VEP** (10 mM) was added to **AcCF** (10 mM) in borate buffer (0.6 M, pH 9.1), a precipitate formed, corresponding to long, broad needles, visualized using confocal microscopy. In contrast, the reaction with **Z^D^VEP** yielded a gel‐like network composed of thin, entangled fibers on the micron scale (Figure S18). These findings indicate that the ability of two epimeric thioesters to assemble into different structures does not necessarily lead to significant differences in their lifetime.

Since histidine (H) is known to catalyze (thio)ester hydrolysis in assembled states,^[^ ^72^ ^]^ we investigated whether it could introduce chirality‐dependent differences in ester lifetime through mechanism 3. Specifically, we hypothesized that in a three‐dimensional assembly, the orientation of histidine side chains and ester groups might allow one diastereomer to form a more efficient catalytic transition state, accelerating hydrolysis selectively. To test this, we acylated **AcYH** (20 mM) with **Z^L/D^VEP** (10 mM) in bicarbonate buffer. In contrast to previous systems, no aggregation was macroscopically observed and both diastereomeric esters exhibited similar lifetimes of ∼ 80 min (Figure 2e, Figure S33). This lack of differentiation might be attributed to the fact that **Z^L/D^V‐AcYH** ester remains fully soluble, as its increased polarity prevents aggregation. To evaluate whether increased hydrophobicity could restore chirality‐dependent assembly and hydrolysis, we substituted valine (V) with phenylalanine (F) in the acylating agent. Indeed, this modification reintroduced aggregation in the D‐driven system, whereas the L‐driven system remained in solution (Figure S28). Moreover, half‐lifetimes for the **Z^L/D^F‐AcYH** ester diverged substantially: 8 min for the L‐driven system and 140 min for the D‐driven system (Figures 2e, S35). Structural analysis revealed differential assembly behaviors. The L‐driven system showed no detectable aggregates under the confocal microscope, although TEM images captured small, transient aggregates (Figure S16). In contrast, the D‐driven system exhibited star‐shaped assemblies, consisting of layered sheets and dense fiber‐like structures. Fluorescence and rheology measurements confirmed that aggregation was significantly stronger in the D‐driven system (Figures S9d and S13c). Taken together, these findings suggest that histidine did not catalyze hydrolysis in the assembled phase, reinforcing the conclusion that the lifetime differences in this system originate from equilibrium shifts in the assembly process (mechanism 2). This observation is consistent with the behavior of **AcXF** containing systems, further supporting the role of stereochemical effects in controlling non‐equilibrium self‐assembly.

Considering the lower hydrophobicity of cysteine (C) compared to tyrosine (Y),^[^ ^73^ ^]^ we introduced a phenylalanine (F) residue into the C‐ and H‐containing peptide substrate to enhance hydrophobic interactions. **AcCHF** (10 mM) was acylated in high yield with **Z^L/D^VEP** (10 mM) in borate buffer. The self‐assembly behavior varied significantly depending on the chirality of the acylating agent: The L‐driven system formed a strong gel that decayed after one month, whereas the D‐driven system led to a precipitate that persisted throughout the entire observation period (Figure 2d). The lifetime differences were also pronounced, with the L‐driven reaction exhibiting a half‐lifetime of 8 days, while the D‐driven system remained stable with a half‐lifetime of 121 days (Figure 2b). Microscopy and structural analysis revealed distinct morphological features. The L‐driven system formed a network of entangled fibers that appeared as thin (∼40 nm) twisted structures in TEM (Figure 3b). In contrast, the D‐driven system formed spindle‐like aggregates several micrometers in size, consisting of broader (∼200 nm) twisted fibers with longer persistence lengths. These structural differences correlated well with macroscopic observations and rheological data, which indicated that the L‐driven assembly was mechanically stronger than the D‐driven system (Figure S13d). Additionally, Nile Red fluorescence intensity was twice as high for the L‐driven gel after three days, further supporting the enhanced self‐assembly in this case (Figure S8b). CD spectroscopy indicated beta‐sheet and random coil secondary structures with negative peaks around 220 and 200 nm for the L‐driven system, while the D‐driven assembly produced a much flatter spectrum with no pronounced signals indicative of a disordered aggregate (Figure S14b). The strong assembly observed in the L‐driven system hydrolyzed significantly faster than its epimeric ester, which may be attributed to histidine‐mediated catalysis. However, when compared to the analogous system containing **AcCF** instead of **AcCHF**, the thioester half‐lifetimes remained similar (7 and 6 days). Therefore, histidine catalysis is unlikely and this system is best described by mechanism 2 (different assembly equilibrium). When changing the acylating agent to **Z^L/D^FEP**, similar thioester lifetimes but differences in morphology were observed with **AcCHF** (Figures S19, S25 and S32). Taken together, these findings illustrate how a reaction cycle can discriminate between enantiomeric acylating agents, enabling control over the reactivity of transient epimers via a chirality‐dependent self‐assembly process.

To better understand the hydrolysis mechanism, we built an extended kinetic model based on mechanism 2 that includes a self‐assembly equilibrium between the epimeric ester in solution and in the self‐assembly (Figure S5). Acylation and hydrolysis were restricted to proceed in solution and only the rate constants for assembly (k_on_) and disassembly (k_off_) were variable, resulting in an estimate for K_eq_ (K_eq _= k_on_/k_off_). As suggested by experiments conducted at high concentrations where no self‐assembly was observed, we assume the acylation and hydrolysis rate in solution to be the same for L‐ and D‐driven systems. We estimated k_acylation, solution, L_ and k_hydrolysis, solution, L_ by measuring the kinetics of each system under conditions where no assembly was observed (Figures S38–S44, Table S5). Reducing the concentration of the acylating agent from 10 to 0.5 mM resulted in clear solutions and Nile Red fluorescence measurements over time showed no transient increase in intensity, indicating that the reactions proceed in solution under these conditions (Figures S10–S12). We then used k_acylation, solution, L_ and k_hydrolysis, solution, L_ as constraints in the extended model to estimate k_on_ and k_off_ of the reactions under assembly conditions. Notably, the estimated K_eq_ matched the observed lifetimes of the assemblies: when comparing L‐ and D‐driven systems, an increase in K_eq_ is correlated with an increase in ester lifetime (Tables S6 and S7). The exchange kinetics also play an important role as a slow k_off_ relative to k_hydrolysis, solution_ can be the rate determining step for the hydrolysis. While this is observed for most systems, some k_off_ values also exceed k_hydrolysis, solution_, yielding a deactivation that is regulated by K_eq_ and hence by the concentration of ester in solution.

### Chirality of an Acylating Agent Directs Downstream Reactivity

**Figure 4:**
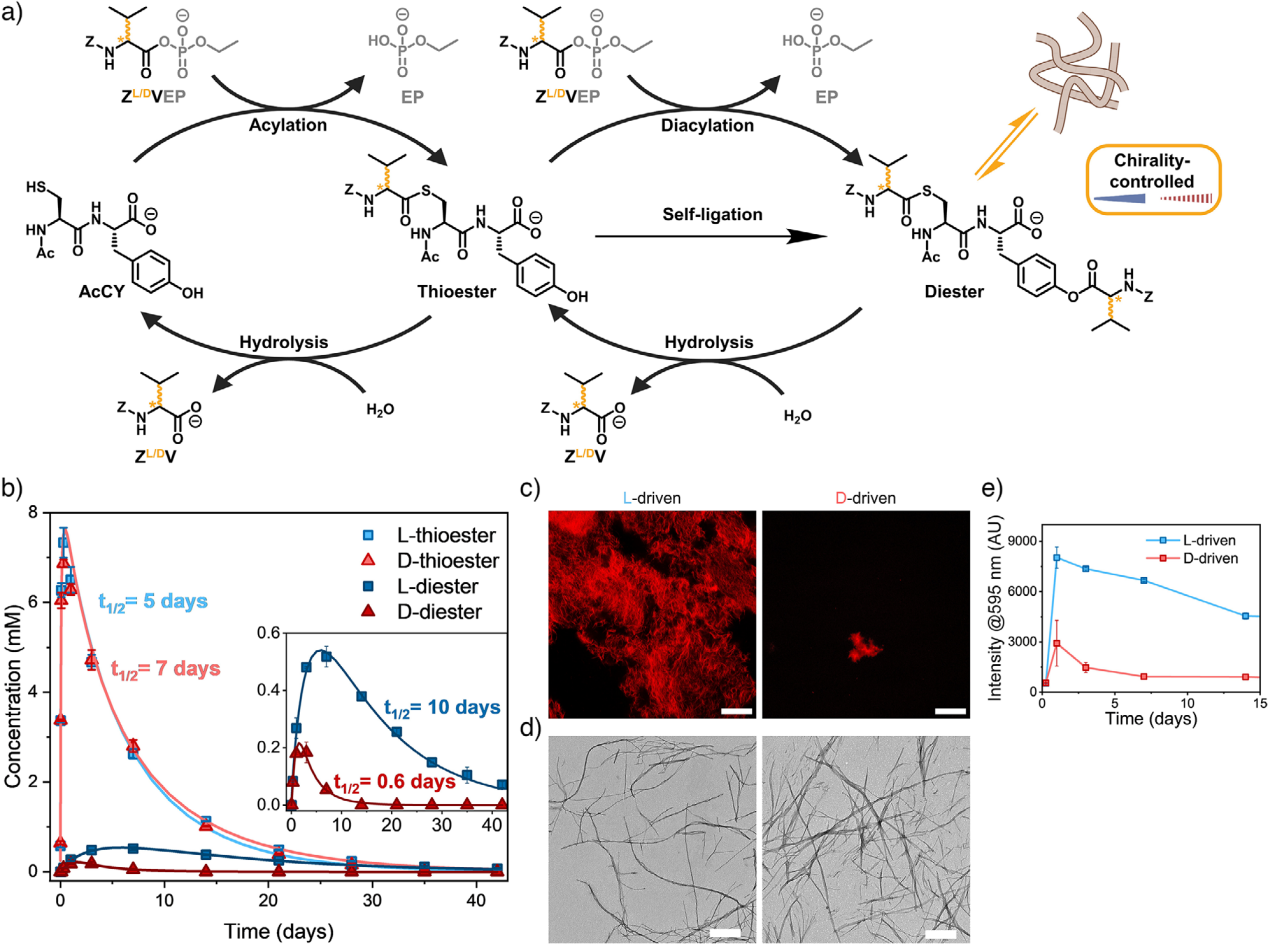
a) Schematic representation of the two coupled reaction cycles starting from the bifunctional **AcCY** peptide substrate. After chirality independent monoacylation, the handedness of the secondary acylating agent (L‐ or D‐thioester) controls the amplitude of the second reaction cycle toward the diester. b) Thioester (light color) and diester (dark color) concentration over time, c) confocal fluorescence microscopy images (Nile Red staining) after 3 days, d) TEM images after 3 days and e) Nile Red fluorescence intensity over time for the reaction of **Z^L/D^VEP** (10 mM) with **AcCY** (10 mM) in borate buffer. The inset in b) highlights the different kinetics of the diester formation and hydrolysis. Scale bars are 20 µm in the confocal images and 500 nm in the TEM images. Error bars represent the standard deviation of three independent experiments.

We moved toward applying the concept of chiral acylating agents to a more complex system that allows us to harvest the reactivity differences of the transient epimers. In biology, series of reactions that occur after an initial signal regulate cellular responses like the ERK1 activation pathway^[^ ^74^ ^]^ or the TGF‐β signaling pathway.^[^ ^75^ ^]^ In many of these cascade reactions, the initial signal occurs via binding of a signaling molecule on a receptor, followed by a complex network of downstream reactions. Thus, we hypothesized that controlling the reactivity of an intermediate through chirality‐dependent interactions could regulate its downstream reactions. Previous studies showed that replacing the aliphatic side chain of aminoacyl phosphate esters with an aromatic one alters self‐assembly, thereby shaping the outcome of cascade reactions.^[^ ^60^ ^]^ We focused on demonstrating that chirality alone without changing functional groups can achieve a similar effect. Thus, we acylated **AcCY** (10 mM), a peptide substrate containing both cysteine (C) and tyrosine (Y) as nucleophilic residues with **Z^L/D^VEP** (10 mM). The reactivity of C was found to be higher than that of Y, as indicated by the greater yields and reaction rates for thioesterification compared to esterification (Figure 2a,b). As a result, the thioester formed first, followed by the formation of the mixed diester, where both C and Y residues were acylated (Figure 4a). Notably, we observed significant differences in the yield and half‐lifetime of the diester. In the L‐driven cascade, the maximum diester yield was twice as high, and its half‐lifetime was more than ten times longer than in the D‐driven cascade (Figure 4b). Two pathways can lead to diester formation: 1) diacylation, where the thioester is acylated a second time by the original acyl phosphate, and 2) self‐ligation, where the thioester undergoes intermolecular transacylation (Figure 4a). While it remains challenging to determine the contribution of each individual pathway to the diester formation, our kinetic model estimates that in the L‐driven cascade ∼0.8 mM diester is formed compared to ∼0.3 mM in the D‐driven cascade (Table S3, Figures S6 and S7). These flux differences demonstrate that the L‐driven system facilitates the formation of an intermediate, that is more reactive toward a subsequent reaction than its D‐driven counterpart. We propose that these differences in kinetics and yield arise from chirality‐dependent interactions of the thioester intermediates as well as chirality‐dependent self‐assembly of the diester product. For the forward reaction, thioester and diester concentrations were similar in both cascades in the first 24 h, indicating that acylation and diacylation occur at comparable rates, while the acyl phosphate ester is still present (Figure 4b). Fluorescence intensity was initially similar at 5 h, but after one day it was 2.5 times higher in the L‐driven cascade compared to the D‐driven system (Figure 4e). This observation suggests that both thioester intermediates remain in solution while the L‐driven diester aggregates stronger than the D‐driven diester.^[^ ^60^ ^]^ After 3 days, the diester concentration reached its maximum conversion in both systems, and dense fibers were observed in both cascades, indicating that both L‐ and D‐acylated diesters assemble (Figure 4d), although to a lesser extent in the D‐driven cascade (Figures 4c,e, S17 and S23). Taken together, these findings indicate that the D‐driven diester not only forms slower but also hydrolyzes faster than the L‐driven diester, resulting in significant differences in concentration profiles. Thus, by controlling the chirality of an acylating agent, we can modulate both the amplitude and the lifetime of a downstream signal via a chirality‐dependent assembly mechanism.

### Chirality of an Acylating Agent Differentiates Between Two Reaction Cycles

**Figure 5:**
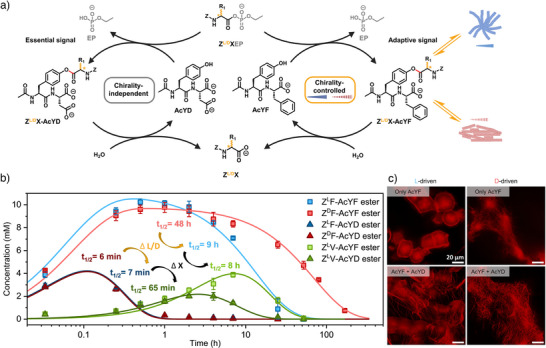
a) Schematic representation of two reaction cycles driven by L‐ or D‐configured aminoacyl phosphate esters: the cycle on the left yields a soluble ester with **AcYD**, while the cycle on the right generates an assembling ester with **AcYF**, resulting in a chirality‐independent and a chirality‐dependent reaction cycle, respectively. (b) Concentration profiles over time for the reaction of **Z^L^FEP** (20 mM, blue) or **Z^D^FEP** (20 mM, red) or **Z^L^VEP** (20 mM, green) with **AcYF** (20 mM, light color) and **AcYD** (20 mM, dark color) in bicarbonate buffer. ∆L/D stands for a change in the chirality of the acylating agent, while ∆X stands for a change in the amino acid of the acylating agent from F to V (change of R_1_ from benzyl to isopropyl). c) Confocal fluorescence images (Nile Red staining) from reactions of **Z^L/D^FEP** (10 mM) with **AcYF** alone (20 mM, top row) and **Z^L/D^FEP** (20 mM) with both **AcYF** (20 mM) and **AcYD** (20 mM, bottom row) after 30 min in bicarbonate buffer. Scale bars: 20 µm. Error bars represent the standard deviation of three independent experiments.

To explore the potential of chiral acylating agents in chemically driven self‐assembly, we introduced a second reaction cycle designed to be chirality‐independent, ensuring a consistent response regardless of the handedness of the acylating agent. In contrast, the primary reaction cycle was designed to be chirality‐sensitive, adapting its behavior depending on the handedness of the acylating agent. This design allows the system to maintain a stable “essential” signal, while simultaneously modulating a dynamic “adaptive” signal (Figure 5a). This concept mirrors biological systems, where essential acylating intermediates, such as Acetyl‐CoA in citric acid cycle,^[^ ^76^ ^]^ are indispensable for cellular respiration, providing a uniform metabolic signal. Meanwhile, adaptive acylation mechanisms, such as ubiquitin conjugation, are tightly regulated to enable selective protein degradation via the proteasome.^[^ ^77^ ^]^ Through the integration of both essential and adaptive cycles, our system could mimic key principles of biochemical regulation, where chirality acts as a molecular switch to tune reaction dynamics.

To establish a chirality‐independent reaction cycle, we selected the negatively charged peptide substrate **AcYD** (D denoting aspartic acid), ensuring that acyl transfer retains the assembly equilibrium toward the solubilized ester state. For the chirality‐dependent cycle, we used **AcYF**, as prior experiments revealed significant differences in reaction lifetimes between L‐ and D‐driven cycles (Figure 2e). To test these two cycles, **Z^L/D^FEP** (1 equivalent) was used as the acylating agent with **AcYD** or **AcYF** (each 2 equivalents) to generate **Z^L/D^F‐AcYD** or **Z^L/D^F‐AcYF** esters (Figure 5a). We conducted the reaction in three different ways: 1) only **AcYF** (Figure 2e), 2) only **AcYD** (Figure S37) and 3) a mixed system where **Z^L/D^FEP** (20 mM) acylated both **AcYF** (20 mM) and **AcYD** (20 mM, Figure 5b). For the hydrophilic, chirality‐independent reaction cycle, half‐lifetimes were nearly identical for L‐ and D‐driven reactions (8 and 7 min, respectively) when **AcYD** was acylated by **Z^L/D^FEP** individually. This trend remained consistent in the mixed system (7 and 6 min), confirming that **AcYD** follows a chirality‐independent pathway. Conversely, the chirality‐dependent reaction cycle retained its distinct lifetime differences. The D‐driven ester had a half‐lifetime of 48 h, while the L‐driven ester decayed with a half‐lifetime of 9 h, mirroring the behavior observed when **AcYF** was used alone (Figure 2e, Table S4). Confocal microscopy confirmed that the assemblies formed in the individual cycles resembled those in the combined system, though minor variations were observed due to potential cross‐interactions between the two cycles (Figure 5c). This behavior shows that our system can preserve one stable, invariant signal while selectively tuning another through chirality.

A key advantage of chiral acylating agents lies in their ability to selectively differentiate between reaction cycles without considerably altering the electronic structure of the activated states. In contrast, conventional strategies often involve modifying substrates or the functional groups of acylating agents, which impacts both the assembly behavior and electronic structure of transient intermediates simultaneously. However, such changes typically affect all reaction steps indiscriminately, making it challenging to target and regulate specific pathways while keeping others constant within a reaction network. We have previously investigated how changing the amino acid derivative of the acylating agent,^[^ ^59^, ^60^ ^]^ from phenylalanine (F) to valine (V) altered self‐assembly behavior and reaction kinetics. While this strategy effectively controlled assembly lifetimes, it also modified the electronic properties of the acyl donor, leading to global changes across multiple reaction cycles. In the current study, when F was replaced with V in the system from Figure 5a, not only the assembling **Z^L/D^V‐AcYF** ester exhibited different kinetics, but also the non‐assembling **AcYD** cycle was significantly affected. While the half‐lifetimes of **Z^L/D^F‐AcYD** esters differed by only one minute (∆L/D), the difference between **Z^L^F‐AcYD** and **Z^L^V‐AcYD** extended to one hour (∆X, Figure 5b). ∆L/D stands for a change in the chirality of the acylating agent, while ∆X stands for a change in the amino acid of the acylating agent from F to V. This strategy demonstrates that chiral acylating agents enable precise control over self‐assembly and reactivity through stereochemical effects, allowing selective pathway differentiation, while preserving fundamental electronic properties of the system.

## Conclusion

Our findings reveal that enantiomeric acylating agents, despite having identical physicochemical properties, induce distinct self‐assembly behaviors. Left‐ and right‐handed acylating agents generate transient epimeric esters from homochiral peptide substrates containing cysteine or tyrosine as nucleophilic residues, leading to supramolecular architectures with striking differences in assembly lifetime, reactivity, morphology and mechanical properties. This highlights the essential role of chirality in directing self‐assembly equilibria and establishes chiral acylating agents as a powerful tool for engineering dynamic synthetic systems with precise structural and temporal control. We leveraged this strategy to regulate downstream reactivity in cascade reactions, where the chirality of the acylating agent governed the reactivity of transient intermediates, influencing the overall reaction outcome. This transient epimerization allowed for selective propagation of molecular transformations, demonstrating how chirality can serve as a determinant for reactivity in chemically driven reaction networks.

Furthermore, in a system of two concurrent reaction cycles, we demonstrated selective modulation of one product while maintaining another invariant – an important property that is difficult to achieve through conventional strategies. Notably, this differentiation was achieved without altering the electronic structure of the acylating agents, but relying instead on chirality‐dependent self‐assembly. Overall, our study provides new insights into how subtle stereochemical variations can shape active self‐assembling systems. Structural elements surrounding activated motifs have been utilized to modulate the properties of dissipative assemblies, influencing their lifetime, dynamics and reactivity. However, achieving selective control often requires extensive molecular design across a large sequence space. In contrast, our findings demonstrate that chirality alone without altering functional groups can direct self‐assembly, reaction kinetics, and pathway selection. This stereochemical variation offers an important direction for controlling non‐equilibrium behavior, providing a compelling alternative to structural diversification. Future investigations into the oligomerization of L‐ and D‐aminoacyl phosphate esters could elucidate how chirality directs the selective formation of oligomers with defined length and composition. Additionally, exploring the effect of non‐enantiopure acylating agents or substrates within chemical reaction networks may provide insights into stereochemical information transfer and amplification in dynamic systems with implications for stereoselective synthesis and the origins of homochirality.

## Supporting Information

The Supporting Information contains a description of materials and methods, HPLC chromatograms, LC‐MS analysis, kinetic profiles, kinetic modeling with COPASI, chemical structures, transmission electron and confocal fluorescence microscopy images, rheology, CD and fluorescence data, macroscopic images as well as synthesis and characterization of acylating agents.

## Conflict of Interests

The authors declare no conflict of interest.

## Supporting information



Supporting information

## Data Availability

The data that support the findings of this study are available from the corresponding author upon reasonable request.
