# Supplementary material for: Chirality Makes or Breaks Chemically Driven Self‐Assembly
Source: Angew Chem Int Ed Engl. 2025 Jul 31;64(38):e202508481. doi: 10.1002/anie.202508481 (PMC12435440; doi:10.1002/anie.202508481)
Supplement: Supplementary file 1 — Supporting information [file ANIE-64-e202508481-s001.pdf]

Supporting Information for:

Chirality Makes or Breaks Chemically Driven Self-Assembly

Lenard Saile,+[a, b] Kun Dai,+[a] Mahesh D. Pol,[a, b] Thejus Pramod,[a, b] Ralf Thomann[a, c] and Charalampos G. Pappas*[a, b]

[a] Lenard Saile, Dr. Kun Dai, Dr. Mahesh D. Pol, Thejus Pramod, Dr. Ralf Thomann, Dr. Charalampos G. Pappas
DFG Cluster of Excellence *liv*MatS @FIT – Freiburg Center for Interactive Materials and Bioinspired Technologies
University of Freiburg, Georges-Köhler-Allee 105, 79110, Freiburg, Germany.
E-mail: charalampos.pappas@livmats.uni-freiburg.de

[b] Lenard Saile, Dr. Mahesh D. Pol, Thejus Pramod, Dr. Charalampos G. Pappas
Institute of Organic Chemistry, University of Freiburg, Albertstrasse 21, 79104, Freiburg, Germany.

[c] Dr. Ralf Thomann
Freiburg Materials Research Center (FMF), University of Freiburg, Stefan-Meier-Strasse 21, 79104, Freiburg, Germany.

1 Table of Contents

2 MATERIALS AND METHODS 3

2.1 Materials 3

2.2 Methods 3

3 SYNTHESIS AND CHARACTERIZATION 5

3.1 General procedures 5

3.2 Characterization 5

4 CHEMICAL STRUCTURES 11

5 KINETIC MODEL 13

6 SUPPORTING FIGURES 25

6.1 Fluorescence 25

6.2 Rheology 28

6.3 Circular Dichroism 29

6.4 Microscopy images 30

6.5 Macroscopic appearance 36

6.6 Plots of concentration over time 40

6.7 Calibration curves 57

6.8 LC-MS analysis 60

6.9 References 79

2 Materials and Methods

2.1 Materials

All reagents were purchased from Sigma-Aldrich and Carl Roth and were used without any further purification unless otherwise indicated. Z-protected amino acids were purchased from Carbolution. Aluminium metal plates precoated with silica gel 60 matrix, 0.25 mm or 0.5 mm were utilized for thin-layer chromatography (TLC). Visualization of the developed TLC plate was performed by irradiation with UV light. Preparative RP-MPLC was performed using an automated Interchim-puriFlash® system.

2.2 Methods

Reaction procedure. Reactions were performed by dissolving the acylating agent in a small volume of 0.1 M MES buffer at pH 5.5 and adding aliquots of this solution to a solution containing the respective peptide in pH-controlled buffer (0.6 M borate buffer at pH 9.1 for cysteine-containing peptides and 0.2 M bicarbonate buffer pH 10.1 for tyrosine-containing peptides) at room temperature.

Transmission Electron Microscopy (TEM). Reactions were vortexed and sonicated prior to sample preparation for imaging. Small drops of the solution, the gel or the suspension were applied to a carboncoated Cu grid for 30 seconds incubation, followed by two drops of water wash and 5 µl of 2% (w/v) uranyl acetate solution for 30 seconds for staining. Excess solution was removed by blotting the grid with a piece of filter paper and left to air dry. Imaging was performed using a FEI Talos 120C at 120 kV operating voltage. Images were taken using a Ceta 16-megapixel camera.

Confocal Microscopy. Imaging was performed on a Zeiss LSM 880 confocal microscope system, equipped with a 63x oil immersion objective. Samples were mixed with 1 mM Nile red in DMSO to reach a final dye concentration of 1 µM and then transferred into ibidi µ-Slide 8-well Bioinert microplates. Samples were excited at 561 nm and emission signals were collected in the range of 575–630 nm.

HPLC. A Shimadzu HPLC LC-40D system equipped with a Shim-pack GIST C18 column (150 mm length, 4.6 mm internal diameter,5 µm particle size) was used to quantify the reactions. All solvent contained 0.1% trifluoroacetic acid. For HPLC-Method 1, the eluting solvent system had a linear gradient starting from 5% (v/v) acetonitrile in water at 0 min to 95% (v/v) acetonitrile in water at 10 min followed by 95% (v/v) acetonitrile in water from 10 to 12 min and 5% (v/v) acetonitrile in water from 12.5 to 16 min. The flow rate was 1 ml/min^{-1}. For the determination of enantiomeric excess (ee) method 2 was used on a Lux i-Cellulose-5 column (150 mm length, 4.6 mm internal diameter,5 µm particle size) where a linear gradient starting from 10% (v/v) acetonitrile in water at 0 min to 45% (v/v) acetonitrile in water at 23 min followed by 95% (v/v) acetonitrile in water from 23.5 to 26 min and 10% (v/v) acetonitrile in water from 26.5 to 30 min. The flow rate was 1 ml/min^{-1}. The ee was determined from the hydrolysis product of the acylating agents. Chromatograms were monitored at 214 nm and the areas under the peaks were used to identify the concentration using a calibration curve. The absorbance of (thio)esters was calculated by summing the absorbance of the peptide and the acylating agent. The HPLC-samples were prepared by immediately splitting the reaction into aliquots of 25-50 µl into individual Eppendorf tubes for each time point during the first 2 minutes of the reaction while the reaction still maintained a solution state. At the corresponding time point, the reaction aliquots were dissolved completely by adding 950 or 975 µl of 50%

(v/v) ACN in water containing 0.1% (v/v) of trifluoroacetic acid. This procedure ensured that the HPLC-sample preparation was identical for all reactions and independent from self-assembly effects for example to avoid issues related to pipetting strong gels. Concentration plots were measured as triplicates of reaction aliquots.

Rheology. Rheological measurements were carried out with an Anton Paar MCR 302 rheometer at 25 °C, using a 25 mm cone-plate geometry (CP25-1, Anton Paar) and a measuring gap of 0.047 mm. Samples were prepared by separating aliquots of 100 µl in the first minute of the reaction into individual Eppendorf tubes and subsequently adding the aliquot on the bottom plate at the timepoint of the measurement. For gel samples, a spatula was used to transfer the gel on the bottom plate. For all the samples, storage moduli (G′) and loss moduli (G″) were measured at a strain amplitude of γ = 5.0 % and a frequency of ω = 1.0 rad/s. A solvent trap was placed around the sample holder to avoid evaporation.

Fluorescence: Fluorescence measurements were carried out at 22 °C on a Microplate Spectrophotometer (Tecan Safire or Agilent H1) equipped with a monochromator. Measurements were performed in a 96-well plate (GRE96fb) that was sealed with Greiner EASYSEAL in between measurements to avoid evaporation of water. Excitation wavelength was 550 nm with a bandwidth of 5 nm and emission wavelength was 595 nm with a bandwidth of 20 nm. A stock solution of 1 mM Nile red in DMSO was added to the samples to reach a final concentration of 1 µM during the measurement.

Circular Dichroism (CD). CD spectra were recorded using a J-810 circular dichroism spectrometer from Jasco at 20 °C, established by a PTC-423S Peltier controller. Samples were placed into a quartz cuvette with 0.5 mm path length. Each spectrum was obtained by scanning wavelength from 300 nm to 190 nm at a scanning rate of 100 nm/min, 2 seconds integration time and 1 nm bandwidth. 5 successive wavelength scans were taken to average for each sample.

UPLC-MS. Ultraperformance liquid chromatography-mass spectrometry (UPLC-MS) experiments were performed on an Agilent 6546 LC/Q-TOT equipped with an Infinity 1290 II in the LC section. We correlated the chromatograms from HPLC analysis with the UPLC chromatograms from UPLC-MS analysis to identify the peaks. The Q-TOF was equipped with a dual AJS ESI source. The experiments were conducted at a VCap voltage of 4000 V, a sheath gas temperature of 300°C and a fragmentor voltage of 120 V. An internal reference was used.

NMR. ^{1}H NMR spectra were recorded on a Bruker 300 MHz spectrometer and Bruker Avance Neo 400 MHz with broadband cryoprobe prodigy. The ^{31}P NMR spectra were recorded on Bruker 162 MHz and Bruker 122 MHz spectrometers using ^{1}H-broad band decoupling in the indicated deuterated solvent. Chemical shifts are reported as delta values from standard peaks.

3 Synthesis and characterization

3.1 General procedures

General procedure 1 of ethyl phosphoric acid (EP):

Ethyl phosphorodichloridate was added dropwise to a ten-fold excess of distilled water in an ice-cooled round-bottom flask. After stirring the reaction mixture for one hour, hydrochloric acid was generated as a by-product and was removed by rotary evaporation. This yielded ethyl phosphoric acid as a clear oil. The oil was freeze-dried and used for coupling reactions without further purification.

General procedure 2 for the synthesis of Z-protected aminoacyl phosphate esters ($Z^{L/D}$XEP):

Synthesis of Z-protected aminoacyl phosphate esters was modified from a reported procedure.[1]

Z-protected amino acid (1.00 g, 3.98 mmol, 1.0 eq), ethyl phosphoric acid (1.18 g, 7.96 mmol, 2.0 eq) and pyridine (0.32 ml, 0.31 g, 3.98 mmol, 1.0 eq) was dissolved in ACN:H_2O (1:4) containing 0.5 M MES buffer and the pH was set to 5.5 with NaOH. EDC (2.28 g, 11.94 mmol, 3 eq) was added and the reaction was stirred for 20-60 minutes at rt. Subsequently, aq. HCl (2 M, 50 ml) was added and the solution extracted with DCM (3 x 50 ml). The combined organic phase was washed with aq. HCl (2 M, 2 x 50 ml). DCM was evaporated under reduced pressure and the remaining residue dissolved in phosphate buffer (2 M, pH 7.4) and the pH set to 7 with NaOH. After flash chromatography (C-18-AQ, linear gradient after 1 CV H_2O/ACN, 8:92) and lyophilization, the product was collected as colorless granulate.

3.2 Characterization

Mass and NMR spectra for Z^LFEP and Z^LVEP were reported previously.[2] HPLC with a chiral stationary phase determined the ee to be >98% for Z^LFEP and >98% for Z^LVEP (see Figure S1 and 2).

3.2.1.1 Ethyl phosphoric acid (EP)

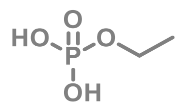

EP

The title compound was synthesized according to general procedure 1. **^{1}H NMR (400 MHz, D_2O)** δ (ppm) 3.99 – 3.89 (m, 2H), 1.21 (td, J = 7.1, 0.7 Hz, 3H); **^{31}P NMR (162 MHz, D_2O)** δ (ppm) 0.05.

3.2.1.2 (R)-2-(((benzyloxy) carbonyl)amino)-3-methylbutanoic (ethyl phosphoric) anhydride sodium salt (Z^DVEP):

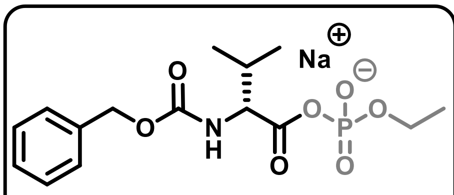

Z^DVEP

The title compound was synthesized according to general procedure 2 as colorless granulate in 28% yield and 98% ee (see Figure S1). **^{1}H NMR (400 MHz, Chloroform-d)** δ 7.31 – 7.26

(m, 5H), 6.11 (d, J = 8.7 Hz, 1H), 5.07 (dd, J = 85.3, 12.2 Hz, 2H), 4.25 (dd, J = 8.7, 5.0 Hz, 1H), 3.95 (t, J = 7.3 Hz, 2H), 2.20 (dt, J = 6.3, 3.4 Hz, 1H), 1.17 (t, J = 7.1 Hz, 3H), 0.91 (dd, J = 26.1, 6.8 Hz, 6H); **^{31}P NMR (162 MHz, Chloroform-d)** δ (ppm) - 6.49.

3.2.1.3 (R)-2-(((benzyloxy)carbonyl)amino)-3-phenylpropanoic (ethyl phosphoric) anhydride sodium salt (Z^{D}FEP):

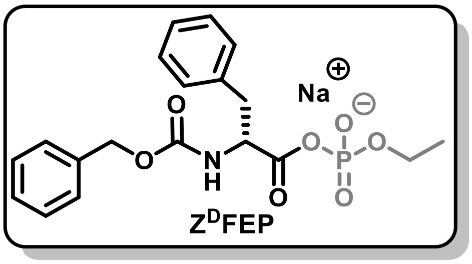

The title compound was synthesized according to general procedure 2 as colorless granulate in 32% yield and >98% ee (see Figure S2). **^{1}H NMR (300 MHz, DMSO-d$_6$)** δ 7.62 (d, J = 8.8 Hz, 1H), 7.37 – 7.18 (m, 10H), 4.95 (s, 2H), 4.22 (ddd, J = 10.9, 8.8, 3.8 Hz, 1H), 3.77 (dq, J = 8.1, 7.1 Hz, 2H), 3.12 (dd, J = 13.9, 3.8 Hz, 1H), 2.79 (dd, J = 13.9, 10.9 Hz, 1H), 1.10 (t, J = 7.1 Hz, 3H); **^{31}P NMR (122 MHz, DMSO-d$_6$)** δ (ppm) - 0.28, - 8.19.

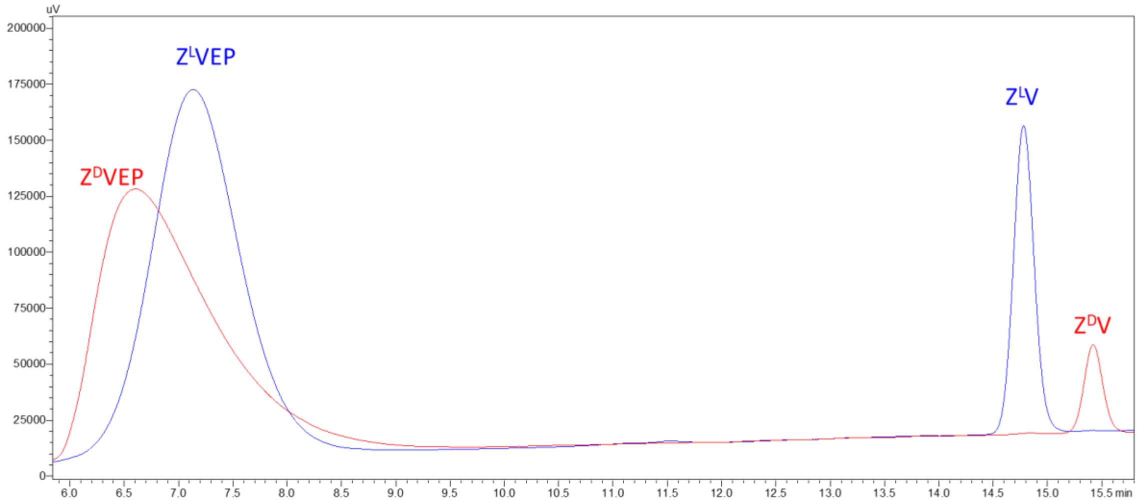

Figure S1: Overlaid chromatograms for partially hydrolyzed Z^{L}VEP and Z^{D}VEP in bicarbonate buffer (0.2 M, pH 10.1) obtained from method 2 using a chiral stationary phase.

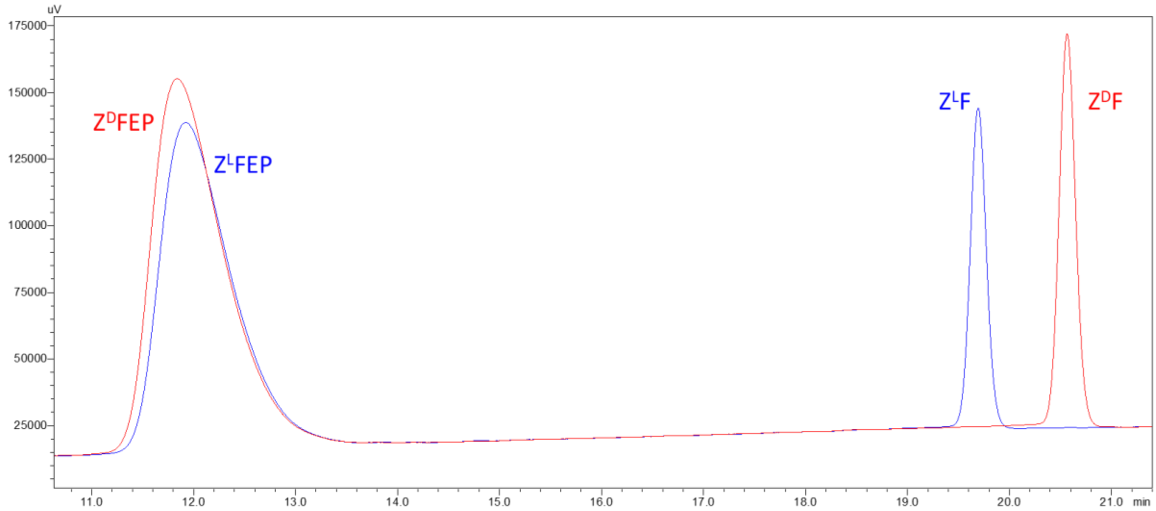

Figure S2: Overlaid chromatograms for partially hydrolyzed Z^{L}FEP and Z^{D}FEP in bicarbonate buffer (0.2 M, pH 10.1) obtained from method 2 using a chiral stationary phase.

EP ^{1}H, 400 MHz, D_2O

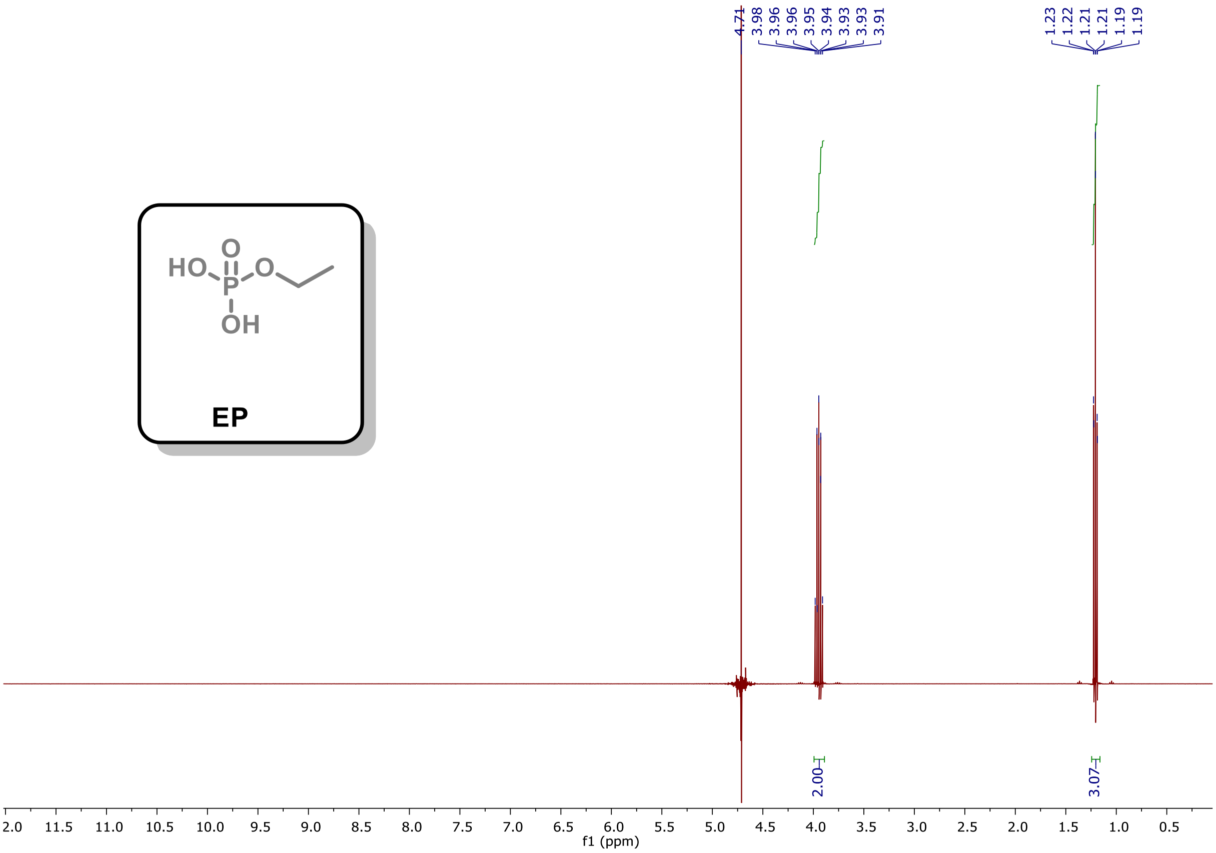

EP ^{31}P, 162 MHz, D_2O

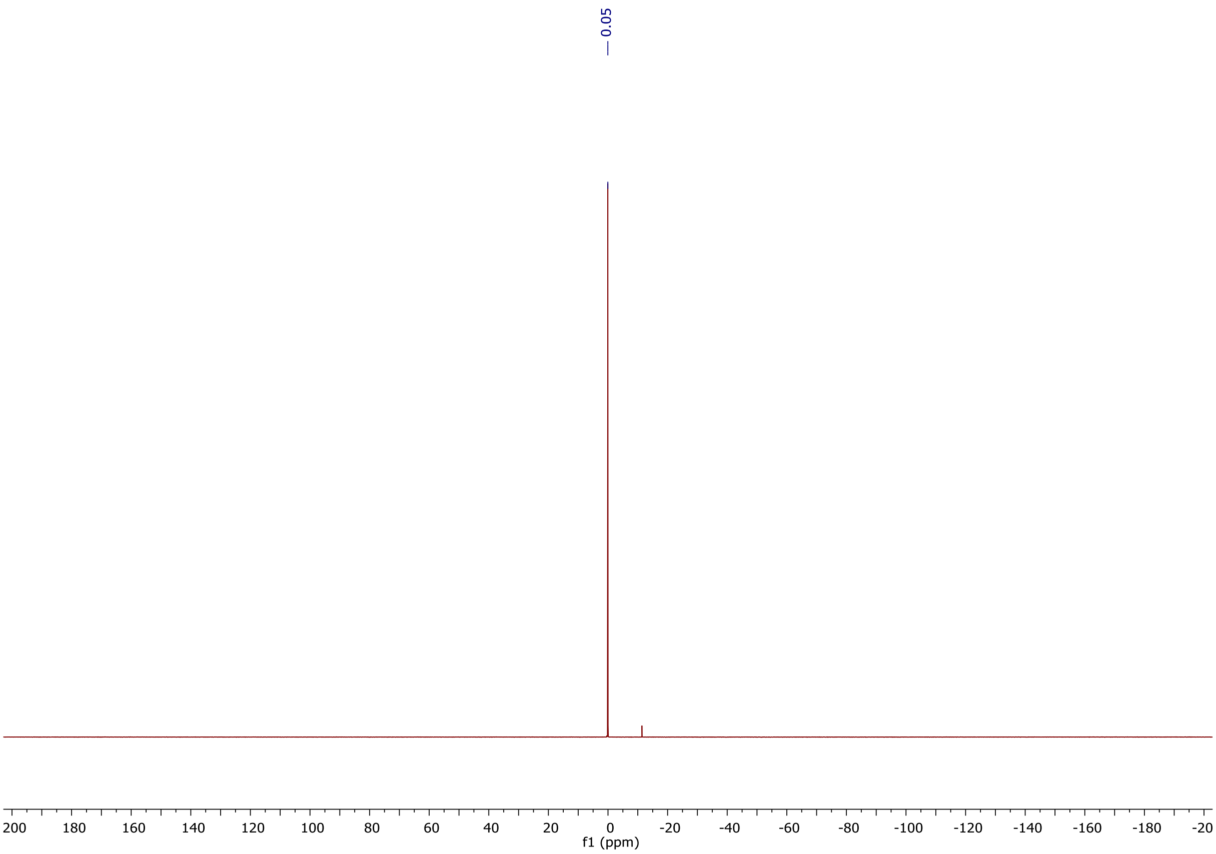

Z^DVEP ¹H, 400 MHz, CDCl₃

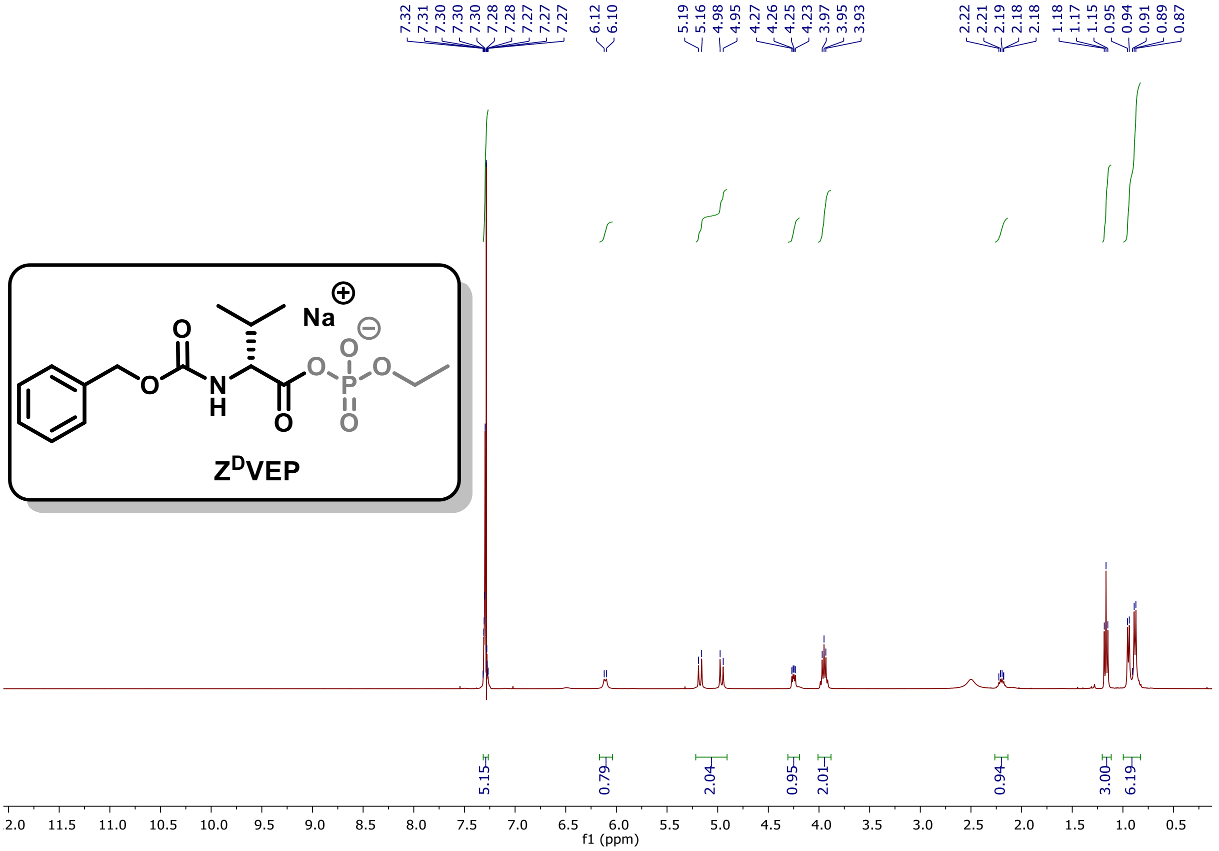

Z^DVEP ³¹P, 162 MHz, CDCl₃

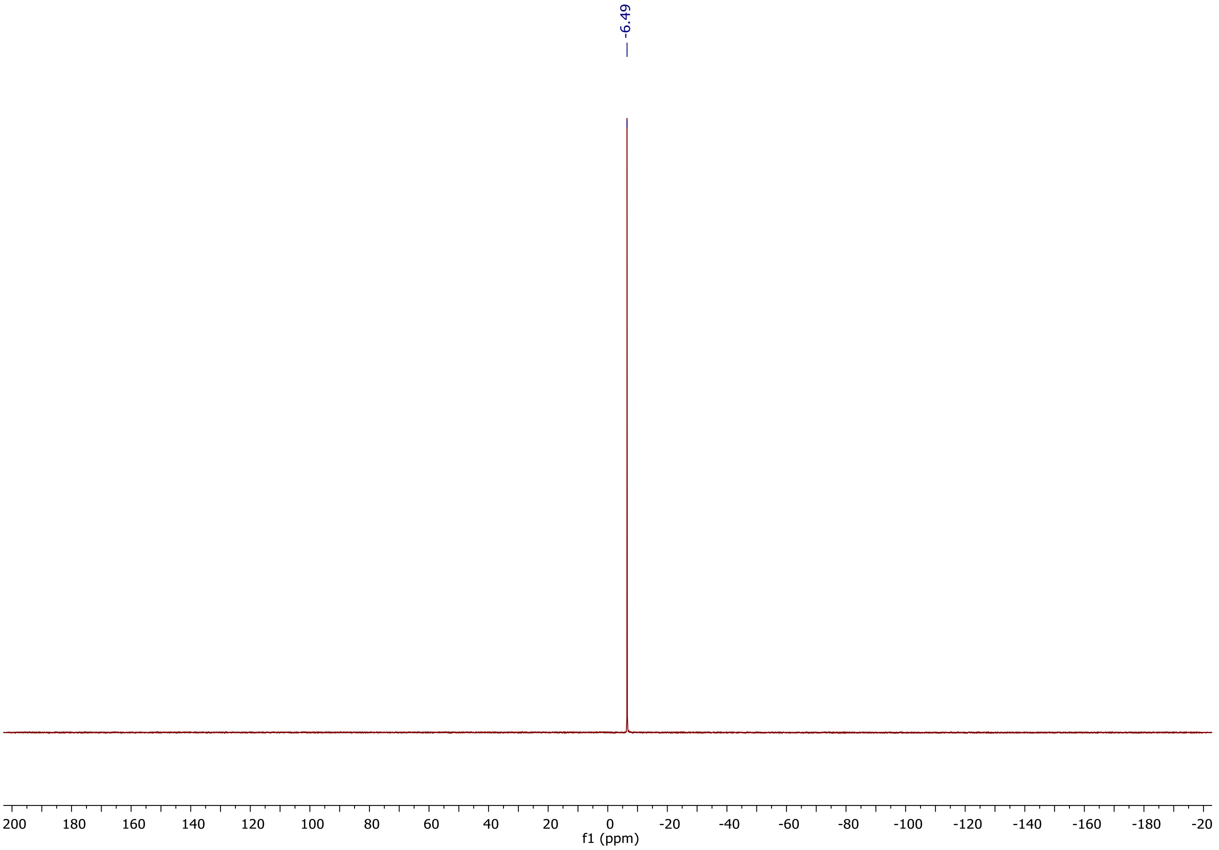

Z^{D}FEP ^{1}H, 300 MHz, DMSO-d$_6$

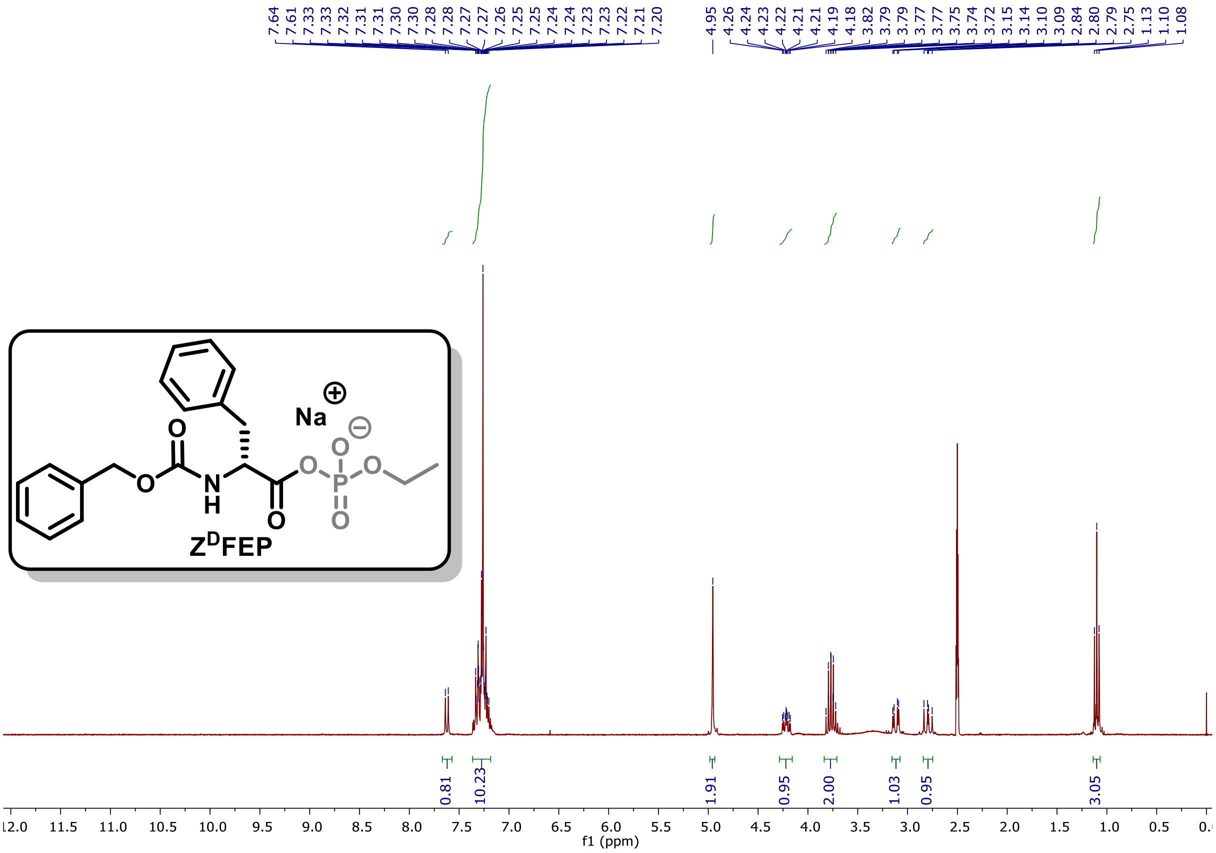

Z^{D}FEP ^{31}P, 122 MHz, DMSO-d$_6$

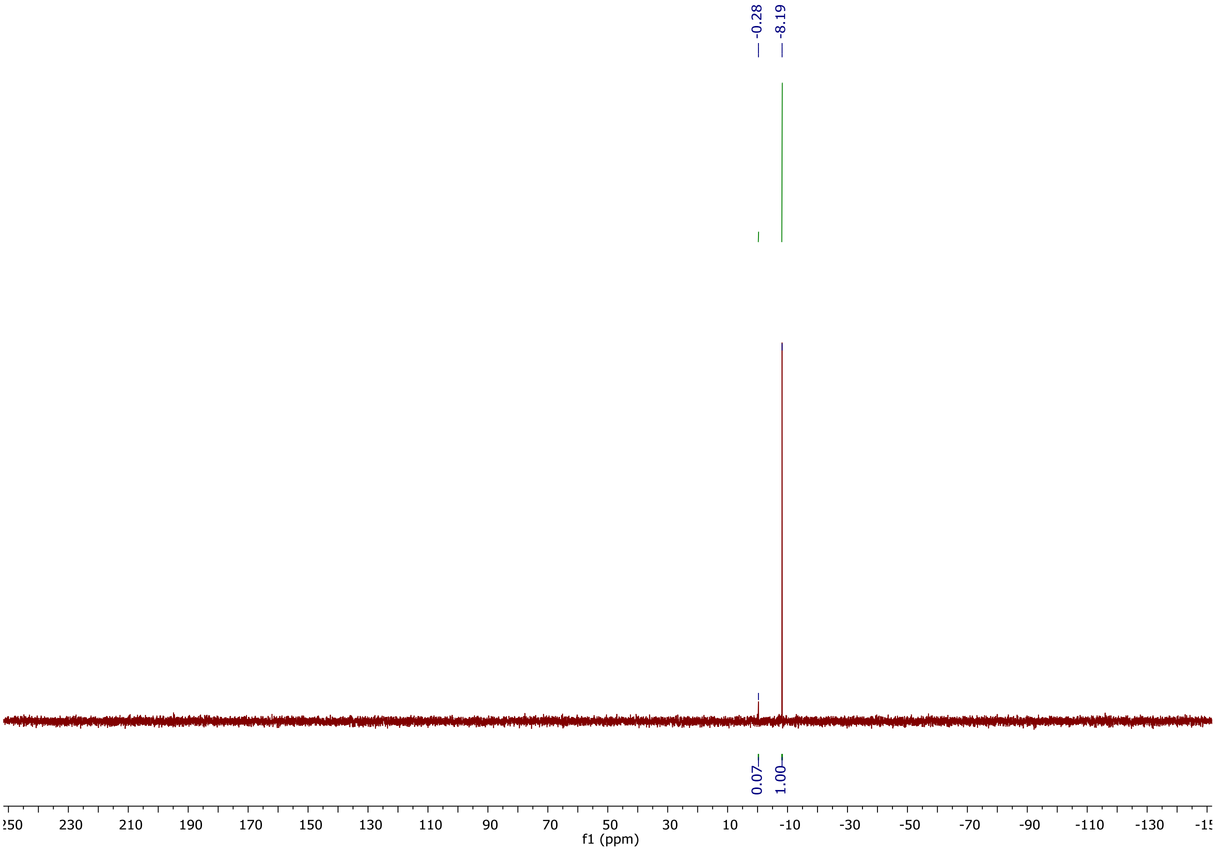

4 Chemical structures

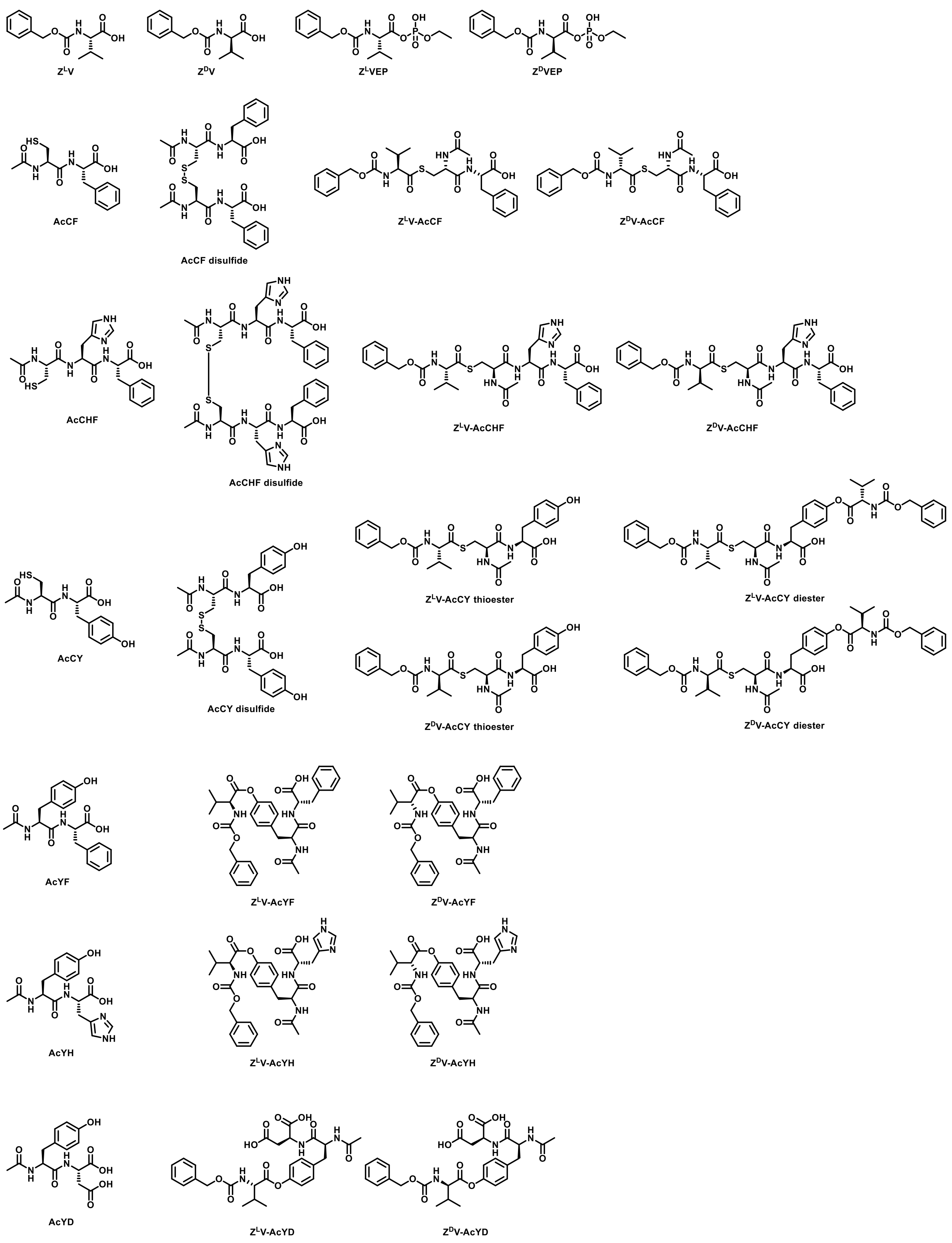

Figure S3: Chemical structures and corresponding abbreviations for Valine (V) containing molecules and peptides as well as their disulfides used in this study.

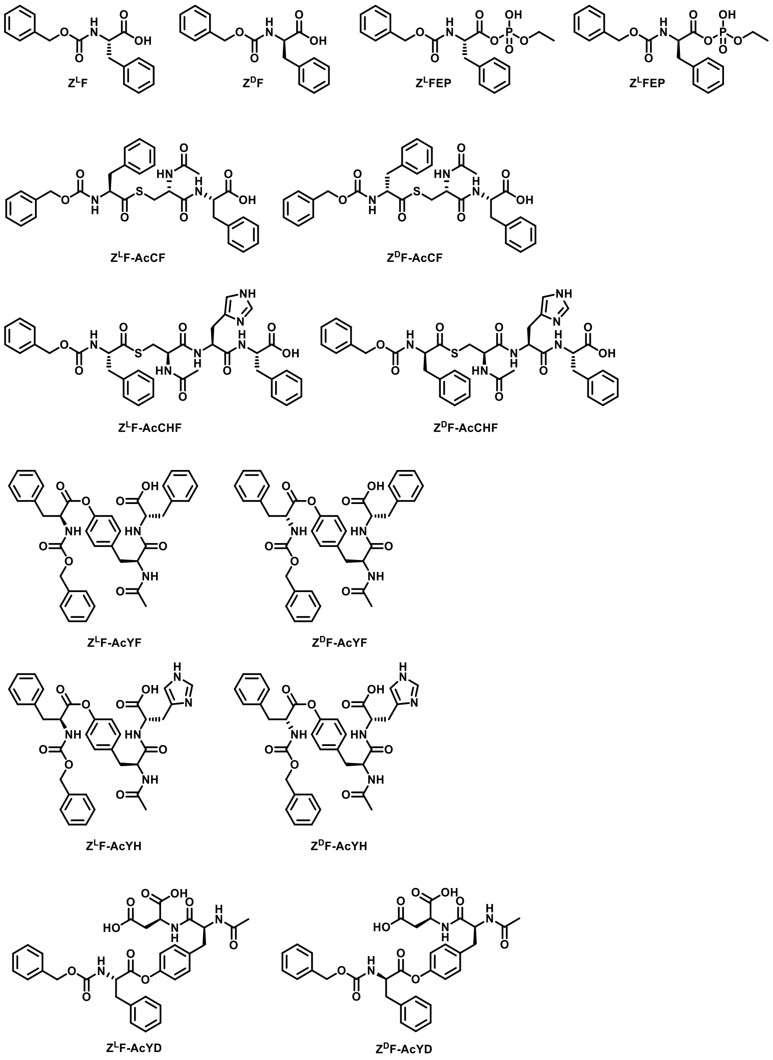

Figure S4: Chemical structures and corresponding abbreviations for Phenylalanine (F) containing molecules used in this study.

5 Kinetic model

Kinetic models were written using the software COPASI.[3] Parameter estimations were performed using evolutionary programming (200 generations, 20 populations) and the experimental (thio)ester concentration profiles from HPLC-measurements. Start values were random, the lower boundary at 1E-9 and the upper boundary at 1E5. Rate constants for the hydrolysis of the acylating agents ($k_{acyl\ phosphate\ hydrolysis}$) were determined empirically in previous publications.[2,4] The rate constants for hydrolysis and acylation in solution were determined by measuring the concentration of (thio)esters in experiments at low concentrations, where no assembly was observed (Figures S10-S12, Table S5). Parameter estimation of these concentration plots yielded $k_{acylation,\ solution,\ L}$ and $k_{hydolysis,\ solution,\ L}$. The hydrolysis rate constants of phosphate esters as well as the rate constants for acylation and hydrolysis in solution were assumed to be identical in the L- and D-driven systems (as suggested by experiments with soluble ester) and the hydrolysis rate at pH 9.1 was found to be a good approximation for all experiments. The rate constants of acylation in solution were assumed to be equal to the total rate constant for acylation ($k_{acylation,\ solution,\ L}= k_{acylation}$, $k_{acylation,\ assembly,\ L}=0$) and used as constraints for the estimation of the observed hydrolysis rate constants ($k_{hydolysis,\ observed,\ L}= k_{hydrolysis,\ solution,\ L} + k_{hydolysis,\ assembly,\ L}$), except for the reaction of $Z^{L/D}$VEP with AcYF, the cascade system (Table S3) and the system with two reaction cycles (Table S4), where $k_{acylation}$ was estimated from the experiment at high concentration. The observed rate constants for the hydrolysis of esters were used to calculate the first-order half-lifetimes of the esters. An extended model was used to estimate the self-assembly equilibrium constants (k_{on}, k_{off}, K_{eq}). As constraints, $k_{acylation,\ solution,\ L}$ and $k_{hydolysis,\ solution,\ L}$ were used and $k_{acylation,\ assembly,\ L}$ and $k_{hydolysis,\ assembly,\ L}$ were set to 0 to only allow acylation and hydrolysis in the disassembled state. The ester concentration profiles under assembly conditions were input as the sum of esters in solution and assembly. Estimated rate constants can be found in Table S1 (individual reaction cycles), S2 (individual reaction cycles with different L:D-ratios), S3 (cascade reaction), S4 (combined reaction cycle with AcYF and AcYD), S5 (experiments in solution conditions) and S6 and S7 (self-assembly equilibrium). Time course simulations were performed with the deterministic (LSODA) method, using the estimated k-values and experimental values (for $k_{acyl\ phosphate\ hydrolysis}$ and $k_{acylation}$). From the time course, flux data was obtained from COPASI and integrated numerically (Figures S6 and S7). A set of ordinary differential equations (ODEs) was used to described the systems and was solved numerically by COPASI. As the reaction only involves one compartment, V_1 can be ignored (set to 1). A general reaction scheme with rate constants for acylation and hydrolysis can be found in Figure S5.

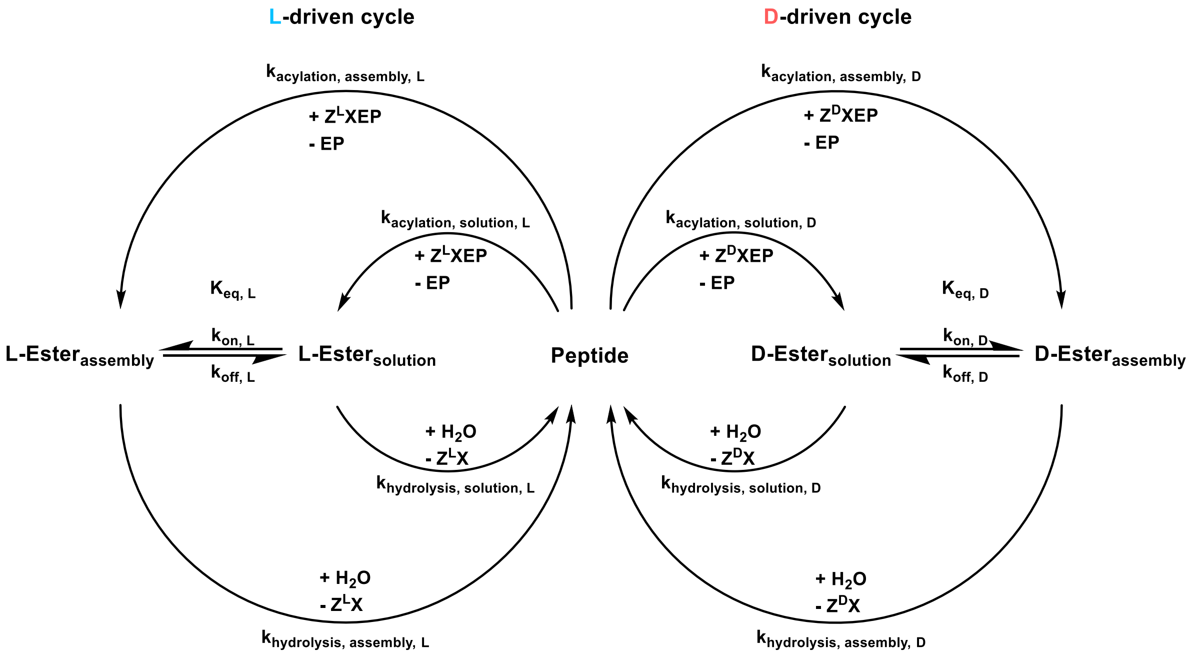

Figure S5: Schematic illustration of acylation and hydrolysis in the assembly and in the solution and how they are connected by the self-assembly equilibrium K_{eq}.

For the individual reaction cycles, the ODEs are:

$$\frac{d([EP]\cdot V_1)}{dt} = +V_1\cdot k1_{(\text{"acyl phosphate hydrolysis"})}\cdot[ZXEP] + V_1\cdot k1_{(\text{"acylation, observed"})}\cdot[ZXEP]\cdot[peptide]$$

$$\frac{d([ZX]\cdot V_1)}{dt} = +V_1\cdot k1_{(\text{"acyl phosphate hydrolysis"})}\cdot[ZXEP] + V_1\cdot k1_{(\text{"hydrolysis, observed"})}\cdot[ester]$$

$$\frac{d([ZXEP]\cdot V_1)}{dt} = -V_1\cdot k1_{(\text{"acyl phosphate hydrolysis"})}\cdot[ZXEP] - V_1\cdot k1_{(\text{"acylation, observed"})}\cdot[ZXEP]\cdot[peptide]$$

$$\frac{d([peptide]\cdot V_1)}{dt} = -V_1\cdot k1_{(\text{"acylation, observed"})}\cdot[ZXEP]\cdot[peptide] + V_1\cdot k1_{(\text{"hydrolysis, observed"})}\cdot[ester]$$

$$\frac{d([ester]\cdot V_1)}{dt} = +V_1\cdot k1_{(\text{"acylation, observed"})}\cdot[ZXEP]\cdot[peptide] - V_1\cdot k1_{(\text{"hydrolysis, observed"})}\cdot[ester]$$

For the cascade reaction, the ODEs are:

$$\frac{d([EP]\cdot V_1)}{dt} = +V_1\cdot k1_{(\text{"acyl phosphate hydrolysis"})}\cdot[ZXEP] +V_1\cdot k1_{(\text{acylation})}\cdot[ZXEP]\cdot[peptide] +V_1\cdot k1_{(\text{diacylation})}\cdot[thioester]\cdot[ZXEP]$$

$$\frac{d([ZX]\cdot V_1)}{dt} = +V_1\cdot k1_{(\text{"acyl phosphate hydrolysis"})}\cdot[ZXEP] +V_1\cdot k1_{(\text{"thioester hydrolysis"})}\cdot[thioester] +2\cdot V_1\cdot k1_{(\text{"diester hydrolysis"})}\cdot[diester]$$

$$\frac{d([ZXEP]\cdot V_1)}{dt} = -V_1\cdot k1_{(\text{"acyl phosphate hydrolysis"})}\cdot[ZXEP] -V_1\cdot k1_{(\text{acylation})}\cdot[ZXEP]\cdot[peptide] -V_1\cdot k1_{(\text{diacylation})}\cdot[thioester]\cdot[ZXEP]$$

$$\frac{d([thioester]\cdot V_1)}{dt} = +V_1\cdot k1_{(\text{acylation})}\cdot[ZXEP]\cdot[peptide] -V_1\cdot k1_{(\text{"thioester hydrolysis"})}\cdot[thioester] -V_1\cdot k1_{(\text{diacylation})}\cdot[thioester]\cdot[ZXEP] -2\cdot V_1\cdot k1_{(\text{"self-ligation"})}\cdot[thioester]\cdot[thioester]$$

$$\frac{d([diester]\cdot V_1)}{dt} = +V_1\cdot k1_{(\text{diacylation})}\cdot[thioester]\cdot[ZXEP] +V_1\cdot k1_{(\text{"self-ligation"})}\cdot[thioester]\cdot[thioester] -V_1\cdot k1_{(\text{"diester hydrolysis"})}\cdot[diester]$$

$$\frac{d([peptide]\cdot V_1)}{dt} = -V_1\cdot k1_{(\text{acylation})}\cdot[ZXEP]\cdot[peptide] +V_1\cdot k1_{(\text{"thioester hydrolysis"})}\cdot[thioester] +V_1\cdot k1_{(\text{"self-ligation"})}\cdot[thioester]\cdot[thioester] +V_1\cdot k1_{(\text{"diester hydrolysis"})}\cdot[diester]$$

For the combined reaction cycles with AcYF and AcYD, the ODEs are:

$$\frac{d([EP]\cdot V_1)}{dt} = +V_1\cdot k1_{(\text{"acyl phosphate hydrolysis"})}\cdot[ZXEP] + V_1\cdot k1_{(\text{"AcYD acylation"})}\cdot[AcYD]\cdot[ZXEP] + V_1\cdot k1_{(\text{"AcYF acylation"})}\cdot[AcYF]\cdot[ZXEP]$$

$$\frac{d([ZX]\cdot V_1)}{dt} = +V_1\cdot k1_{(\text{"acyl phosphate hydrolysis"})}\cdot[ZXEP] + V_1\cdot k1_{(\text{"FAcYD ester hydrolysis"})}\cdot[XAcYDester] + V_1\cdot k1_{(\text{"FAcYF ester hydrolysis"})}\cdot[XAcYFester]$$

$$\frac{d([ZXEP]\cdot V_1)}{dt} = -V_1\cdot k1_{(\text{"acyl phosphate hydrolysis"})}\cdot[ZXEP] - V_1\cdot k1_{(\text{"AcYD acylation"})}\cdot[AcYD]\cdot[ZXEP] - V_1\cdot k1_{(\text{"AcYF acylation"})}\cdot[AcYF]\cdot[ZXEP]$$

$$\frac{d([XAcYDester]\cdot V_1)}{dt} = +V_1\cdot k1_{(\text{"AcYD acylation"})}\cdot[AcYD]\cdot[ZXEP] - V_1\cdot k1_{(\text{"FAcYD ester hydrolysis"})}\cdot[XAcYDester]$$

$$\frac{d([XAcYFester]\cdot V_1)}{dt} = +V_1\cdot k1_{(\text{"AcYF acylation"})}\cdot[AcYF]\cdot[ZXEP] - V_1\cdot k1_{(\text{"FAcYF ester hydrolysis"})}\cdot[XAcYFester]$$

$$\frac{d([AcYD]\cdot V_1)}{dt} = -V_1\cdot k1_{(\text{"AcYD acylation"})}\cdot[AcYD]\cdot[ZXEP] + V_1\cdot k1_{(\text{"FAcYD ester hydrolysis"})}\cdot[XAcYDester]$$

$$\frac{d([AcYF]\cdot V_1)}{dt} = -V_1\cdot k1_{(\text{"AcYF acylation"})}\cdot[AcYF]\cdot[ZXEP] + V_1\cdot k1_{(\text{"FAcYF ester hydrolysis"})}\cdot[XAcYFester]$$

For the estimation of the self-assembly equilibrium constants $k1_{self\text{-}assembly}$ and $k2_{self\text{-}assembly}$ (k_{on} and k_{off}) with the expanded model the ODEs are:

$$\frac{d([EP]\cdot V_1)}{dt} = +V_1\cdot k1_{(\text{"acyl phosphate hydrolysis"})}\cdot[ZXEP] + V_1\cdot k1_{(\text{acylation})}\cdot[ZXEP]\cdot[\text{peptide}]$$

$$\frac{d([ZX]\cdot V_1)}{dt} = +V_1\cdot k1_{(\text{"acyl phosphate hydrolysis"})}\cdot[ZXEP] + V_1\cdot k1_{(\text{"ester hydrolysis solution"})}\cdot[\text{"ester solution"}]$$

$$\frac{d([ZXEP]\cdot V_1)}{dt} = -V_1\cdot k1_{(\text{"acyl phosphate hydrolysis"})}\cdot[ZXEP] - V_1\cdot k1_{(\text{acylation})}\cdot[ZXEP]\cdot[\text{peptide}]$$

$$\frac{d([\text{peptide}]\cdot V_1)}{dt} = -V_1\cdot k1_{(\text{acylation})}\cdot[ZXEP]\cdot[\text{peptide}] + V_1\cdot k1_{(\text{"ester hydrolysis solution"})}\cdot[\text{"ester solution"}]$$

$$\frac{d([\text{"ester solution"}]\cdot V_1)}{dt} = +V_1\cdot k1_{(\text{acylation})}\cdot[ZXEP]\cdot[\text{peptide}] - V_1\cdot k1_{(\text{"ester hydrolysis solution"})}\cdot[\text{"ester solution"}] - V_1\cdot(k1_{(\text{"self-assembly"})}\cdot[\text{"ester solution"}] - k2_{(\text{"self-assembly"})}\cdot[\text{"ester assembly"}])$$

$$\frac{d([\text{"ester assembly"}]\cdot V_1)}{dt} = +V_1\cdot(k1_{(\text{"self-assembly"})}\cdot[\text{"ester solution"}] - k2_{(\text{"self-assembly"})}\cdot[\text{"ester assembly"}])$$

$$\text{"[sum esters]"} = \text{"[ester assembly]"} + \text{"[ester solution]"}$$

Table S1: Estimated rate constants for the observed (thio)ester hydrolysis with standard deviation for the reaction of $Z^{L/D}$XEP (10 mM) with AcCX (10 mM) in borate buffer (0.6 M, pH 9.1) or with AcYX (20 mM) in bicarbonate buffer (0.2 M, pH 10.1). Half-lifetimes $t_{1/2}$ of the (thio)ester hydrolysis were calculated according to its rate constant in first order kinetics.

**reaction**	**$k_{hydrolysis, observed}$ (min^{-1})**	**standard deviation (min^{-1})**	**$t_{1/2}$ (h)**
**Z^LVEP + AcCF**	6.73E-05	7.87E-06	171.757
**Z^DVEP + AcCF**	7.49E-05	9.84E-06	154.234
**Z^LVEP + AcCHF**	5.71E-05	5.04E-06	202.247
**Z^DVEP + AcCHF**	3.99E-06	5.79E-07	2897.596
**Z^LFEP + AcCF**	4.56E-04	3.28E-05	25.315[a]
**Z^DFEP + AcCF**	7.84E-04	3.55E-05	14.732
**Z^LFEP + AcCHF**	8.34E-05	3.22E-06	138.436
**Z^DFEP + AcCHF**	1.13E-04	8.57E-06	102.483
**Z^LVEP + AcYF**	2.28E-03	5.49E-05	5.056[b]
**Z^DVEP + AcYF**	2.28E-04	3.17E-06	50.669[b]
**Z^LVEP + AcYH**	8.46E-03	7.55E-04	1.365
**Z^DVEP + AcYH**	8.58E-03	8.38E-04	1.346
**Z^LFEP + AcYF**	1.41E-03	7.98E-04	8.175
**Z^DFEP + AcYF**	5.34E-04	6.46E-05	21.631
**Z^LFEP + AcYH[c]**	8.34E-02	1.74E-03	0.138
**Z^DFEP + AcYH[c]**	4.95E-03	4.08E-04	2.335

**Z^{L}FEP + AcYD**	8.41E-02	2.01E-03	0.137
**Z^{D}FEP + AcYD**	9.90E-02	3.85E-03	0.117
**Z^{L}VEP + AcYD**	7.60E-03	6.11E-04	1.519

[a] Differences with our previously reported half-lifetime[4] (12 h) arise because a different methodology was used to measure the concentration profile and to fit the ester decay. Reactions were not vortexed and sonicated as previously but rather aliquots of the reaction were separated at the beginning of the reaction and dissolved completely before HPLC injection. Additionally, in our previous publication, we fitted the decay directly without considering the acylation and the acyl phosphate hydrolysis happening at the same time. When considering these reactions and fitting the previous data using the model described above with COPASI, we found the previous half-lifetime to be 20 h, which is much more comparable to the result in this work (25 h). [b] $k_{acylation}$ was estimated from the reaction at 10 mM concentration ($k_{acylation}$=3.66E-05±5.46E-07) [c] $k_{acyl\ phosphate\ hydrolysis}$ was set to 0.5 min^{-1} to account for fast hydrolysis due to histidine in this reaction.

Table S2: Estimated rate constants for the observed ester hydrolysis with standard deviation for the reaction of Z^{L}VEP and Z^{D}VEP with different L:D-ratios (total Z$^{L/D}$VEP concentration of 10 mM) with AcYF (20 mM) in bicarbonate buffer (0.2 M, pH 10.1). Half-lifetimes $t_{1/2}$ of the (thio)ester hydrolysis were calculated according to its rate constant in first order kinetics.

**L:D-ratio**	**$k_{hydrolysis,\ observed}$ (min^{-1})**	**standard deviation (min^{-1})**	**$t_{1/2}$ (h)**
**100:0**	2.28E-03	5.49E-05	5.056
**75:25**	1.47E-03	3.67E-05	7.849
**50:50**	9.34E-04	3.38E-05	12.373
**25:75**	4.71E-04	2.39E-05	24.531
**0:100**	2.28E-04	3.17E-06	50.669

Table S3: Estimated rate constants for thioester and diester hydrolysis, acylation, diacylation and self-ligation with standard deviation and root mean square of the estimation for the reaction of $Z^{L/D}$VEP (10 mM) with AcCY (10 mM) in borate buffer (0.6 M, pH 9.1).

reaction	Z^{L}VEP + AcCY		Z^{D}VEP + AcCY	
	rate constant (min^{-1})	standard deviation (min^{-1})	rate constant (min^{-1})	standard deviation (min^{-1})
**thioester hydrolysis**	9.95E-05	8.72E-06	1.13E-04	1.61E-05
**acylation**	1.29E-03	1.17E-04	9.24E-04	1.23E-04
**diacylation**	1.02E-05	4.09E-06	1.87E-05	2.19E-06
**self-ligation**	2.80E-06	2.48E-07	6.96E-07	3.28E-07
**diester hydrolysis**	4.54E-05	3.50E-06	1.73E-04	5.13E-05
**Root mean square**	0.032		0.016	

Table S4: Estimated rate constants for ester hydrolysis and acylation with standard deviation and root mean square of the estimation for the reaction of $Z^{L/D}$XEP (20 mM) with AcYF (20 mM) and AcYD (20 mM) in bicarbonate buffer (0.2 M, pH 10.1).

reaction	Z^{L}FEP + AcYF + AcYD		Z^{D}FEP + AcYF + AcYD		Z^{L}VEP + AcYF + AcYD	
	rate constant (min^{-1})	standard deviation (min^{-1})	rate constant (min^{-1})	standard deviation (min^{-1})	rate constant (min^{-1})	standard deviation (min^{-1})
**AcYD acylation**	5.38E-03	3.52E-04	5.07E-03	2.86E-04	1.08E-04	8.15E-06
**ZX-AcYD ester hydrolysis**	9.47E-02	5.72E-03	1.11E-01	5.09E-03	1.06E-02	8.68E-04
**AcYF acylation**	8.50E-03	9.12E-04	6.19E-03	4.87E-04	7.11E-05	4.24E-06
**ZX-AcYF ester hydrolysis**	1.23E-03	1.90E-04	2.41E-04	2.77E-05	1.42E-03	1.61E-04
**Root mean square**	0.174		0.154		0.173	

Table S5: Estimated rate constants for ester hydrolysis and acylation with standard deviation for the reactions of Z^LXEP (0.5 mM) with AcCX (0.5 mM) in borate buffer (0.6 M, pH 9.1) or with AcYX (1 mM) in bicarbonate buffer (0.2 M, pH 10.1). Half-lifetimes $t_{1/2}$ of the (thio)ester hydrolysis were calculated according to its rate constant in first order kinetics. Plots of concentration over time can be found in Figures S38-S44.

reaction	$k_{hydrolysis, solution, L}$ (min^{-1})	standard deviation (min^{-1})	$t_{1/2}$ (h)	$k_{acylation, solution, L}$ (min^{-1})	standard deviation
**Z^LVEP + AcCF**	2.28E-04	9.65E-07	50.569	4.59E-03	2.49E-05
**Z^LVEP + AcCHF**	2.21E-04	3.21E-06	52.156	7.21E-03	1.46E-04
**Z^LFEP + AcCF**	1.50E-03	2.47E-05	7.720	2.11E-01	5.07E-03
**Z^LFEP + AcCHF**	4.51E-03	1.09E-04	2.564	3.48E-01	3.96E-02
**Z^LVEP + AcYF**	1.75E-02	1.41E-03	0.658	2.54E-04	2.01E-05
**Z^LFEP + AcYF**	1.47E-01	9.87E-03	0.079	5.72E-03	4.38E-04
**Z^LFEP + AcYH**	1.64E-01	4.23E-03	0.070	1.53E-02	5.41E-04

Table S6: Estimated rate constants from the extended kinetic model for assembly and disassembly of (thio)esters (k_{on} and k_{off}) with standard deviations and the equilibrium constants K_{eq} for the reactions of $Z^{L/D}$XEP (10 mM) with AcCX (10 mM) in borate buffer (0.6 M, pH 9.1) or with AcYX (20 mM) in bicarbonate buffer (0.2 M, pH 10.1).

**reaction**	**k_{on} (min^{-1})**	**standard deviation (min^{-1})**	**k_{off} (min^{-1})**	**standard deviation (min^{-1})**	**K_{eq}**
**Z^LVEP + AcCF**	1.18E-03	1.05E-03	4.65E-04	4.37E-04	2.528
**Z^DVEP + AcCF**	6.04E-04	3.99E-04	2.61E-04	1.86E-04	2.316
**Z^LVEP + AcCHF**	1.11E-03	8.03E-04	4.62E-06	3.81E-06	239.549
**Z^DVEP + AcCHF**	1.13E-03	6.16E-04	1.50E-07	2.37E-07	7528.369
**Z^LFEP + AcCF**	9.17E+01	1.05E+03	4.02E+01	4.61E+02	2.280
**Z^DFEP + AcCF**	6.82E-01	2.81E+01	7.50E-01	3.09E+01	0.909
**Z^LFEP + AcCHF**	1.86E-01	1.09E-01	3.49E-03	2.07E-03	53.386
**Z^DFEP + AcCHF**	8.52E+00	4.43E+02	2.19E-01	1.14E+01	38.993
**Z^LVEP + AcYF**	6.35E+00	1.35E+02	9.51E-01	2.03E+01	6.680
**Z^DVEP + AcYF**	1.13E+00	9.83E-01	1.47E-02	1.29E-02	76.638
**Z^LFEP + AcYF**	1.69E-01	9.04E-02	1.20E-03	1.21E-03	140.752
**Z^DFEP + AcYF**	2.27E-01	6.60E-02	6.00E-04	3.36E-04	378.872
**Z^LFEP + AcYH**	6.31E-02	8.89E-02	8.29E-02	1.53E-01	0.761
**Z^DFEP + AcYH**	5.95E-02	9.89E-03	2.98E-03	1.14E-03	19.992

Table S7: Estimated rate constants from the extended model for assembly and disassembly of (thio)esters (k_{on} and k_{off}) with standard deviations and the equilibrium constants K_{eq} for the reactions of $Z^{L/D}$XEP (20 mM) with AcYF (20 mM) and AcYD (20 mM) in bicarbonate buffer (0.2 M, pH 10.1).

reaction	k_{on} (min^{-1})	standard deviation (min^{-1})	k_{off} (min^{-1})	standard deviation (min^{-1})	K_{eq}
**Z^LFEP + AcYF + AcYD**	5.45E+01	1.15E+03	4.37E-01	9.26E+00	124.766
**Z^DFEP + AcYF + AcYD**	1.09E+00	4.25E-01	1.95E-03	8.48E-04	560.834
**Z^LVEP + AcYF + AcYD**	1.41E+01	3.75E+02	9.04E-01	2.41E+01	15.551

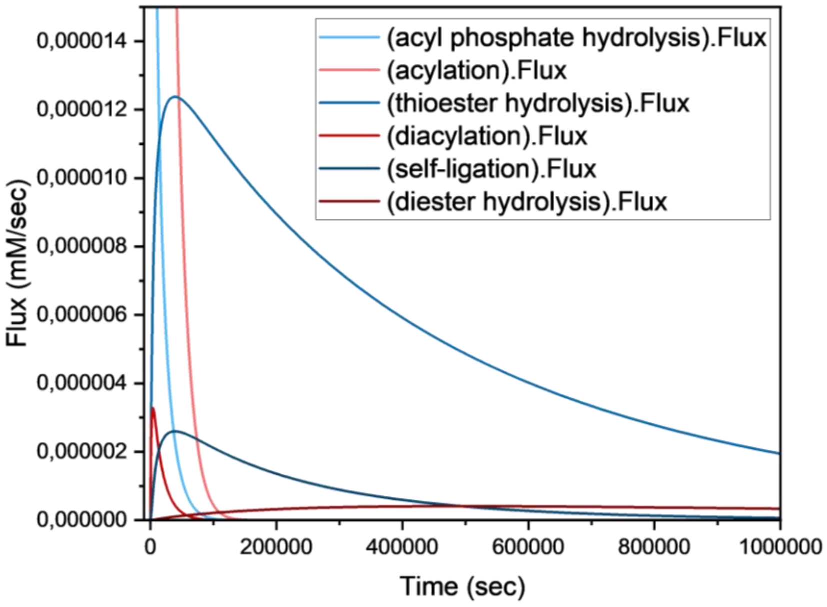

Figure S6: Flux over time for the reaction of Z^LVEP (10 mM) with AcCY (10 mM) in borate buffer (0.6 M, pH 9.1). Integrated fluxes over the first 8 weeks (completion of reaction) are 0.1 mM for the diacylation, 0.7 mM for the self-ligation and 0.8 mM for the diester hydrolysis. The relative contributions of diacylation and self-ligation are rough estimates and emphasis is given to the sum (0.8 mM).

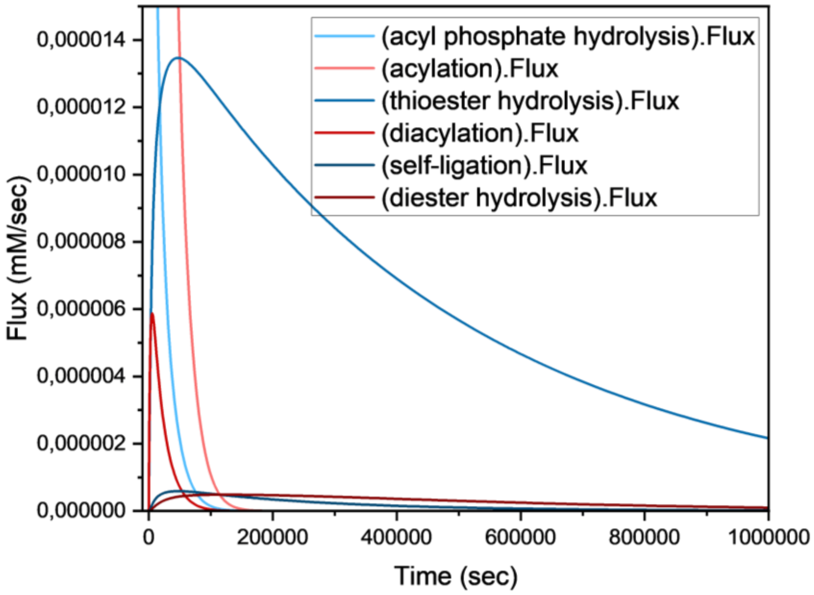

Figure S7: Flux over time for the reaction of Z^{D}VEP (10 mM) with AcCY (10 mM) in borate buffer (0.6 M, pH 9.1). Integrated fluxes over the first 8 weeks (completion of reaction) are 0.1 mM for the diacylation, 0.2 mM for the self-ligation and 0.3 mM for the diester hydrolysis. The relative contributions of diacylation and self-ligation are rough estimates and emphasis is given to the sum (0.3 mM).

6 Supporting Figures

6.1 Fluorescence

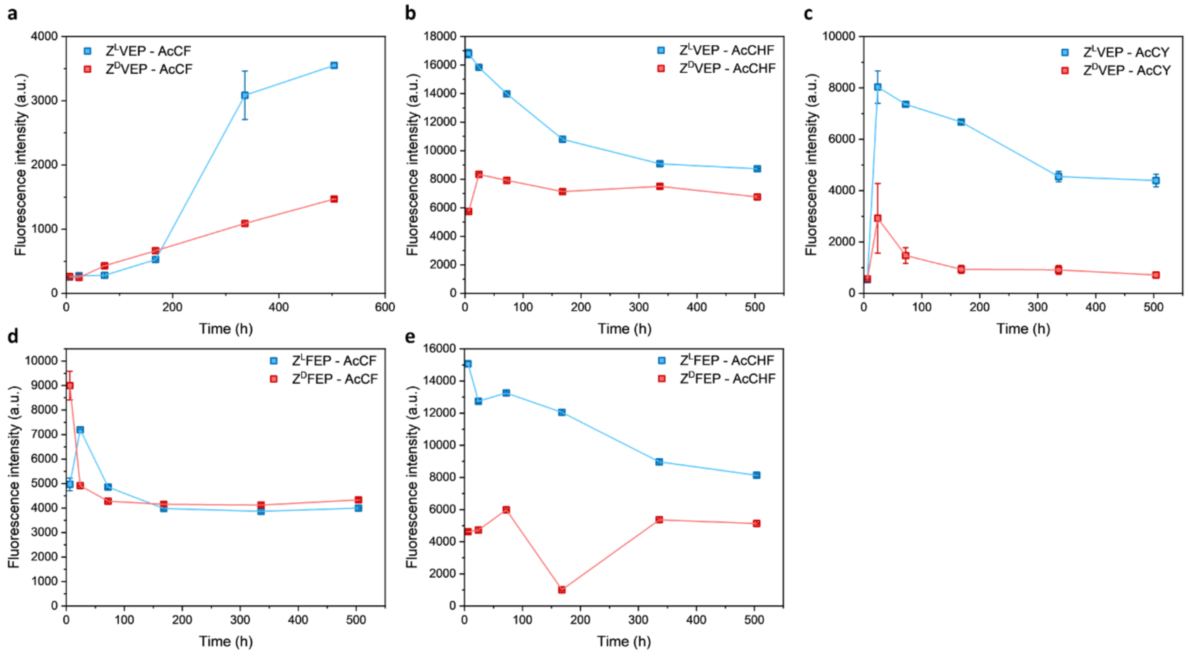

Figure S8: Fluorescence intensity over time for the reaction of $Z^{L/D}$VEP (10 mM) with a) AcCF (10 mM), b) AcCHF (10 mM), c) AcCY (10 mM) and $Z^{L/D}$FEP (10 mM) with d) AcCF (10 mM) and e) AcCHF (10 mM). The final concentration of Nile Red in the samples was 1 µM. Error bars represent the standard deviation of three independent experiments.

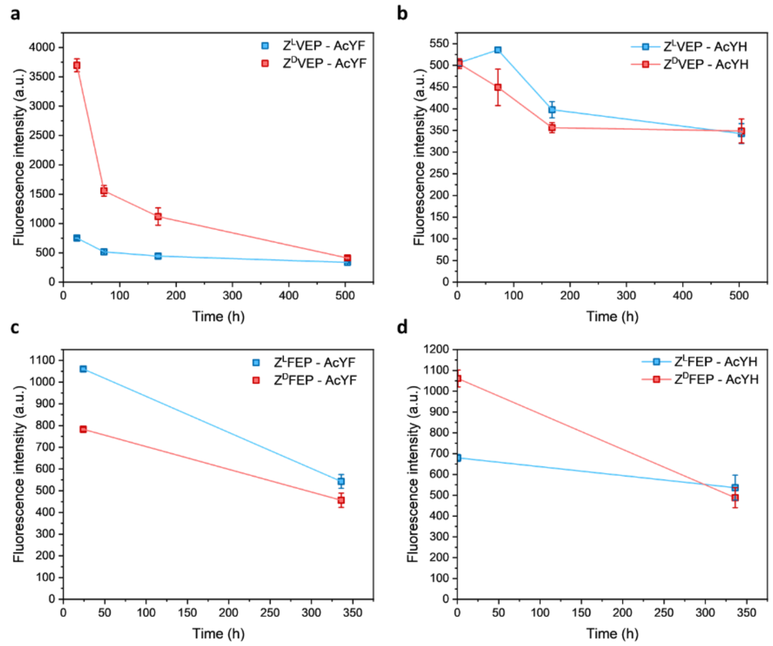

Figure S9: Fluorescence intensity over time for the reaction of $Z^{L/D}$VEP (10 mM) with a) AcYF (20 mM) and b) AcYH (20 mM) and $Z^{L/D}$FEP (10 mM) with c) AcYF (20 mM) and d) AcYH (20 mM). The final concentration of nile red in the samples was 1 µM. Error bars represent the standard deviation of three independent experiments.

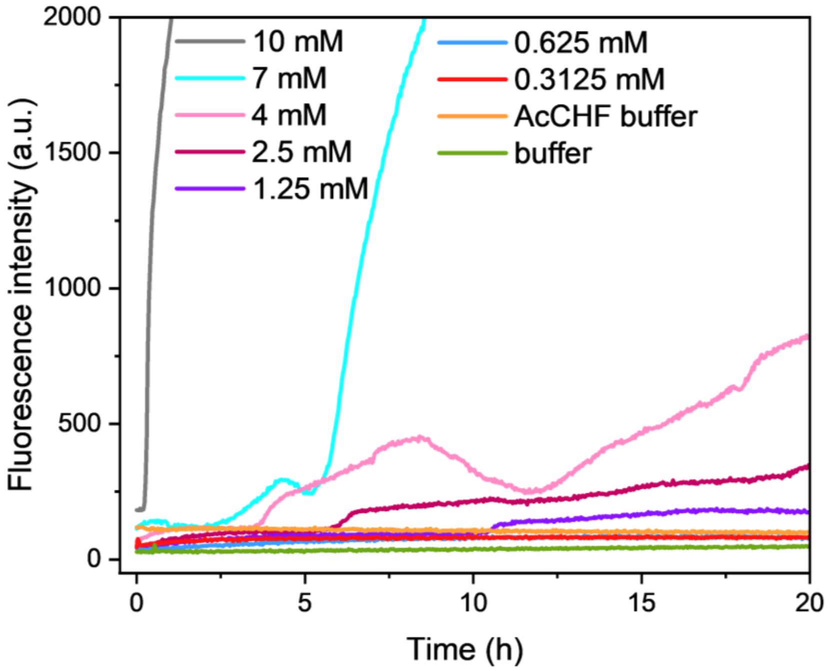

Figure S10: Fluorescence intensity over time for the reaction of Z^{L}VEP (1 eq) with AcCHF (1 eq) in borate buffer (0.6 M, pH 9.1). Borate buffer and AcCHF (2.5 mM) in borate buffer were used as controls. The final concentration of Nile Red in all samples was 1 µM. A lid was placed on the plate to reduce evaporation.

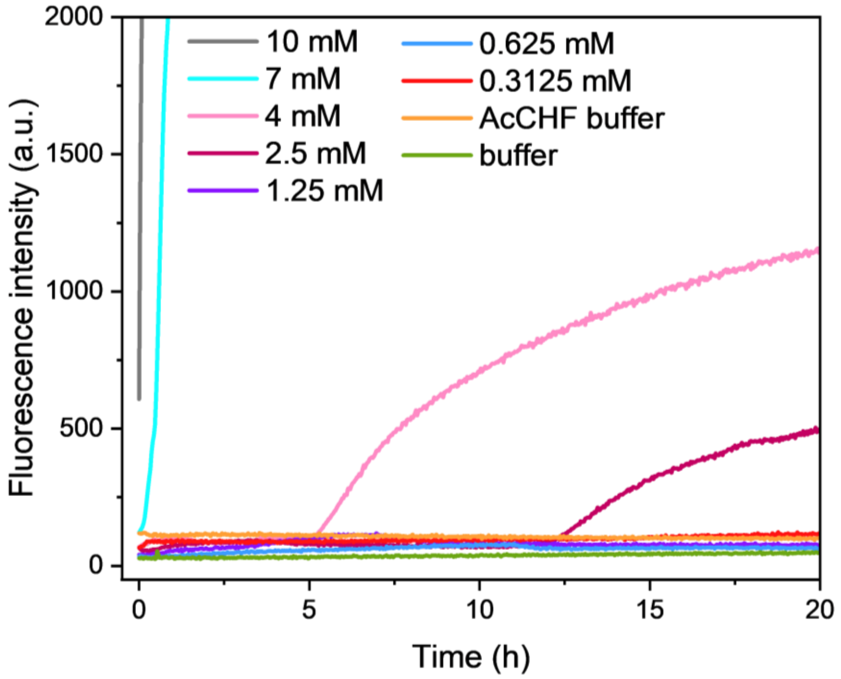

Figure S11: Fluorescence intensity over time for the reaction of Z^{D}VEP (1 eq) with AcCHF (1 eq) in borate buffer (0.6 M, pH 9.1). Borate buffer and AcCHF (2.5 mM) in borate buffer were used as controls. The final concentration of Nile Red in all samples was 1 µM. A lid was placed on the plate to reduce evaporation.

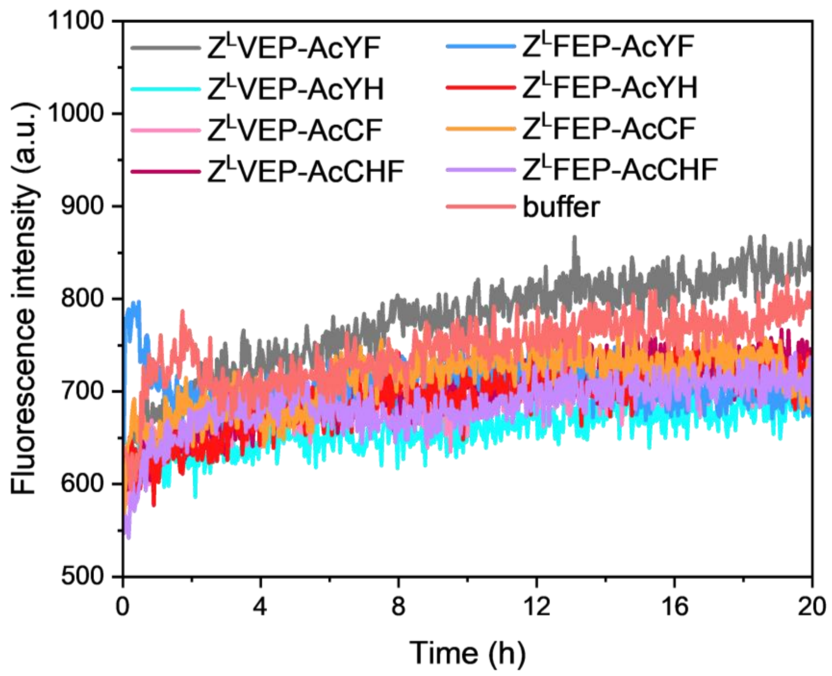

Figure S12: Fluorescence intensity over time for the reaction of Z^{L}FEP (0.5 mM) or Z^{L}VEP (0.5 mM) with AcYF (1 mM), AcYH (1 mM)), AcCF (0.5 mM), AcCHF (0.5 mM) and AcCY (0.5 mM). Borate buffer was used as control. The final concentration of Nile Red in all samples was 1 µM. A slight increase of fluorescence intensity can be attributed to up-concentration of the dye by evaporation. A lid was placed on the plate to reduce evaporation.

6.2 Rheology

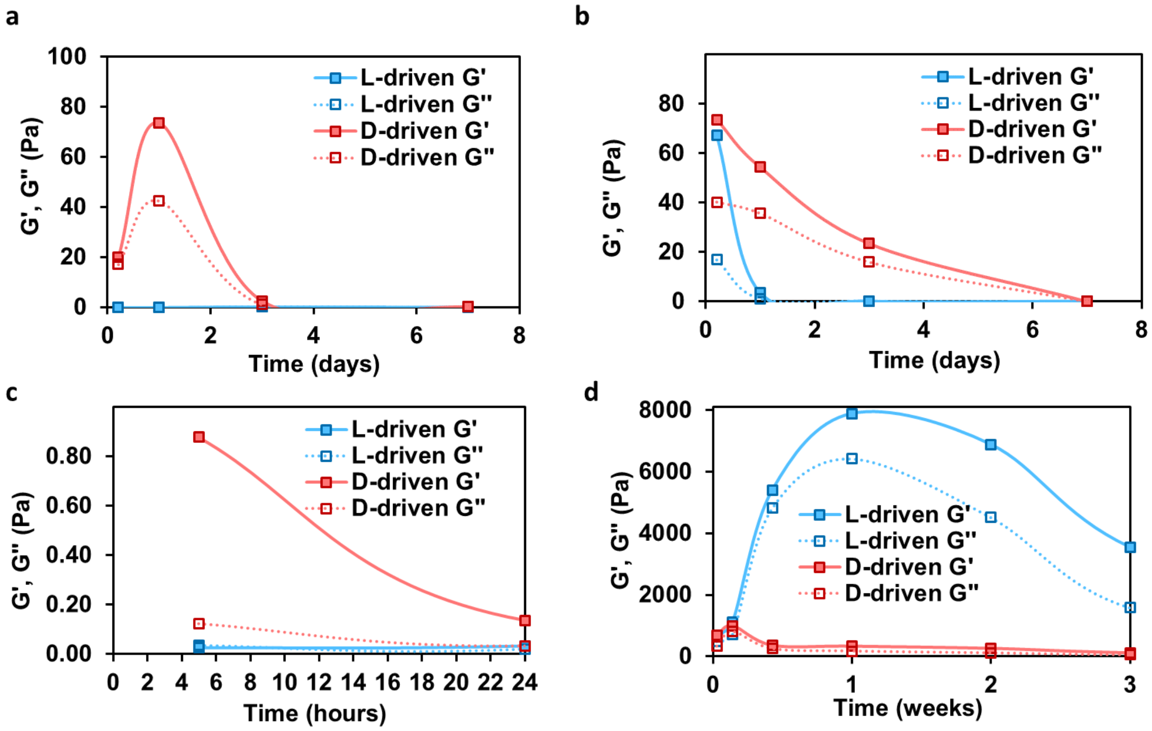

Figure S13: Storage (G’) and loss modulus (G’’) over time for the system containing a) $Z^{L/D}$VEP (10 mM) and AcYF (20 mM) in bicarbonate buffer (0.2 M, pH 10.1), b) $Z^{L/D}$FEP (10 mM) and AcYF (20 mM) in bicarbonate buffer (0.2 M, pH 10.1), c) $Z^{L/D}$FEP (10 mM) and AcYH (20 mM) in bicarbonate buffer (0.2 M, pH 10.1) and d) $Z^{L/D}$VEP (10 mM) and AcCHF (10 mM) in borate buffer (0.6 M, pH 9.1). The solid squares represent the storage modulus (G′) and the open squares represent the loss modulus (G″).

6.3 Circular Dichroism

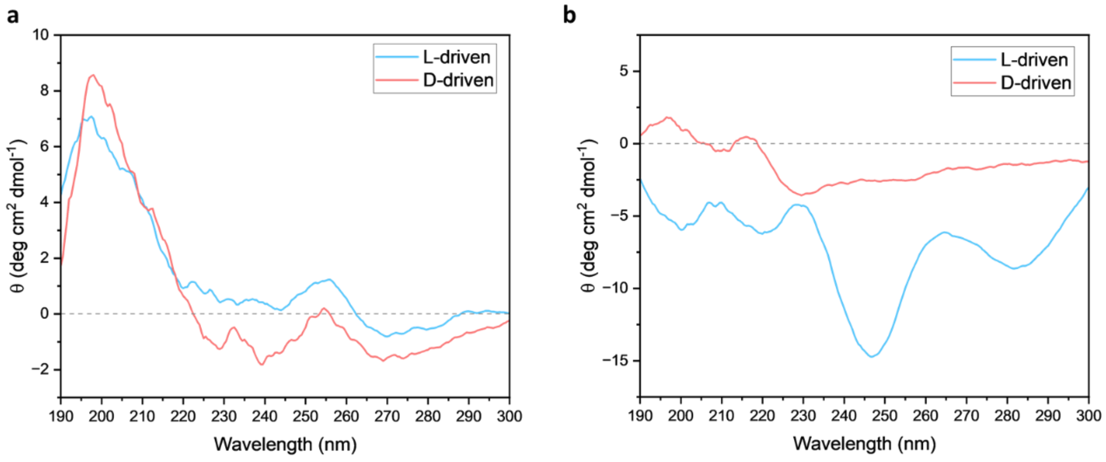

Figure S14: CD-spectra for the reaction of $Z^{L/D}$VEP (10 mM) with a) AcYF (20 mM) and b) AcCHF (10 mM) after 6 h.

6.4 Microscopy images

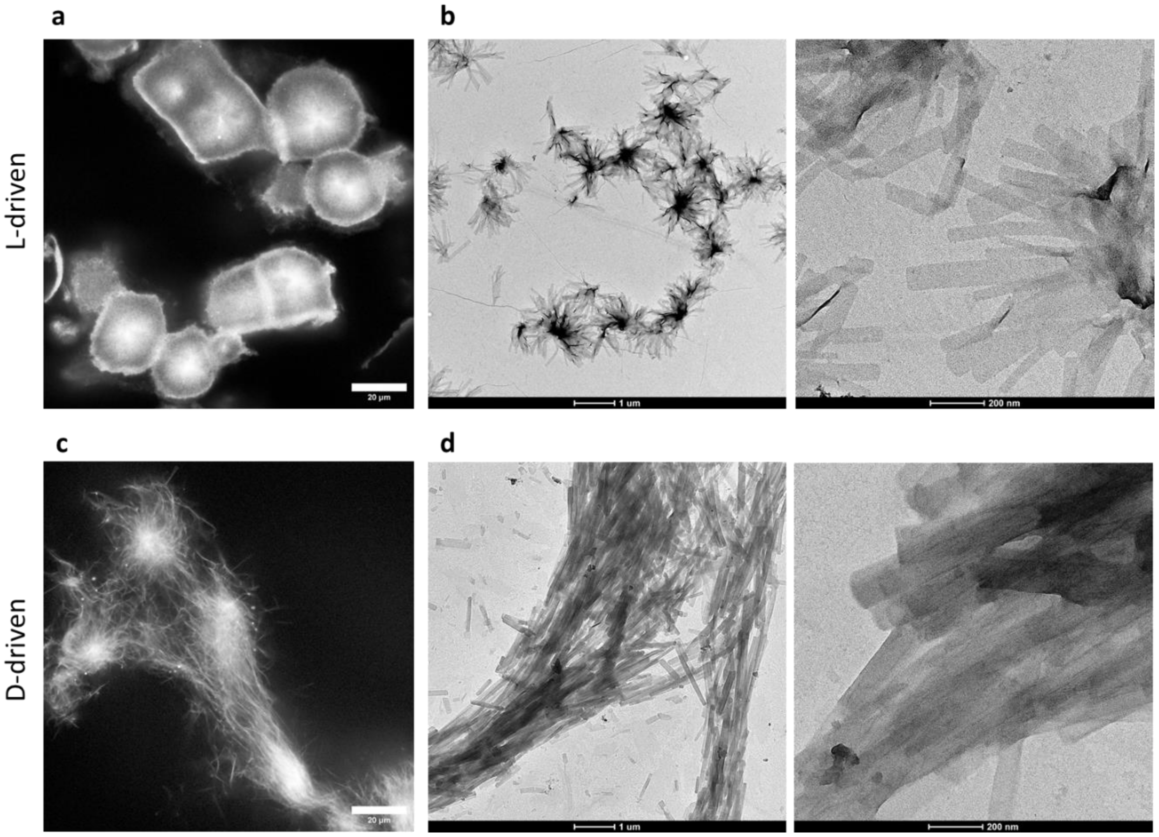

Figure S15: Confocal (a,c) and TEM (b, d) images of the reaction of Z$^{L/D}$FEP (10 mM) with AcYF (20 mM) in bicarbonate buffer (0.2 M, pH 10.1) after 3 h reaction time. a) and b) show the left-handed system while c) and d) show the right-handed system. The scale bar for the confocal images is 20 µm and the scale bars for the TEM images are 1 µm (left) and 200 nm (right). The sample for confocal microscopy was stained with 1 µM Nile Red and the samples for TEM were stained with uranyl acetate.

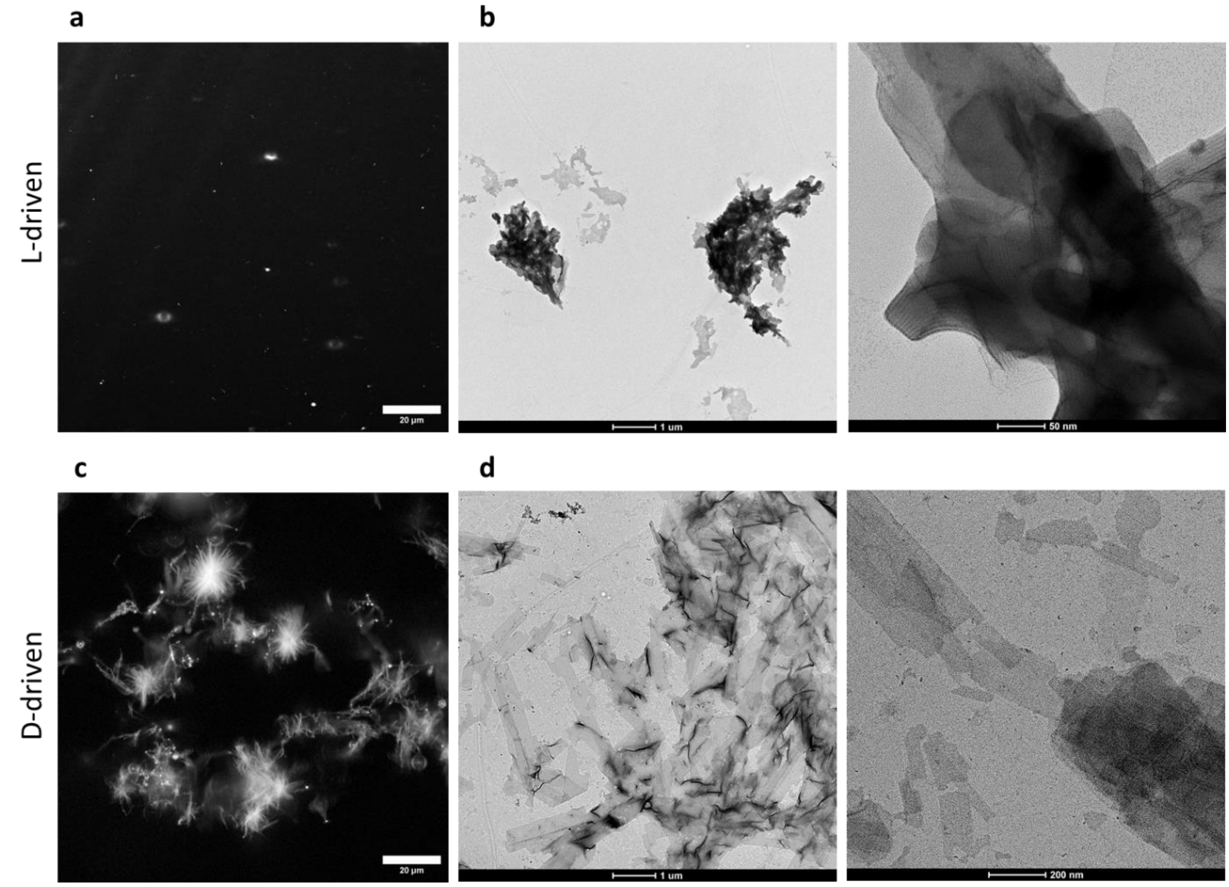

Figure S16: Confocal (a,c) and TEM (b, d) images of the reaction of Z$^{L/D}$FEP (10 mM) with AcYH (20 mM) in bicarbonate buffer (0.2 M, pH 10.1) after 1 h reaction time. a) and b) show the left-handed system while c) and d) show the right-handed system. The scale bar for the confocal images is 20 µm and the scale bars for the TEM images are 1 µm (b, d, left) and 200 nm (b, right) and 50 nm (d, right). The sample for confocal microscopy was stained with 1 µM Nile Red and the samples for TEM were stained with uranyl acetate.

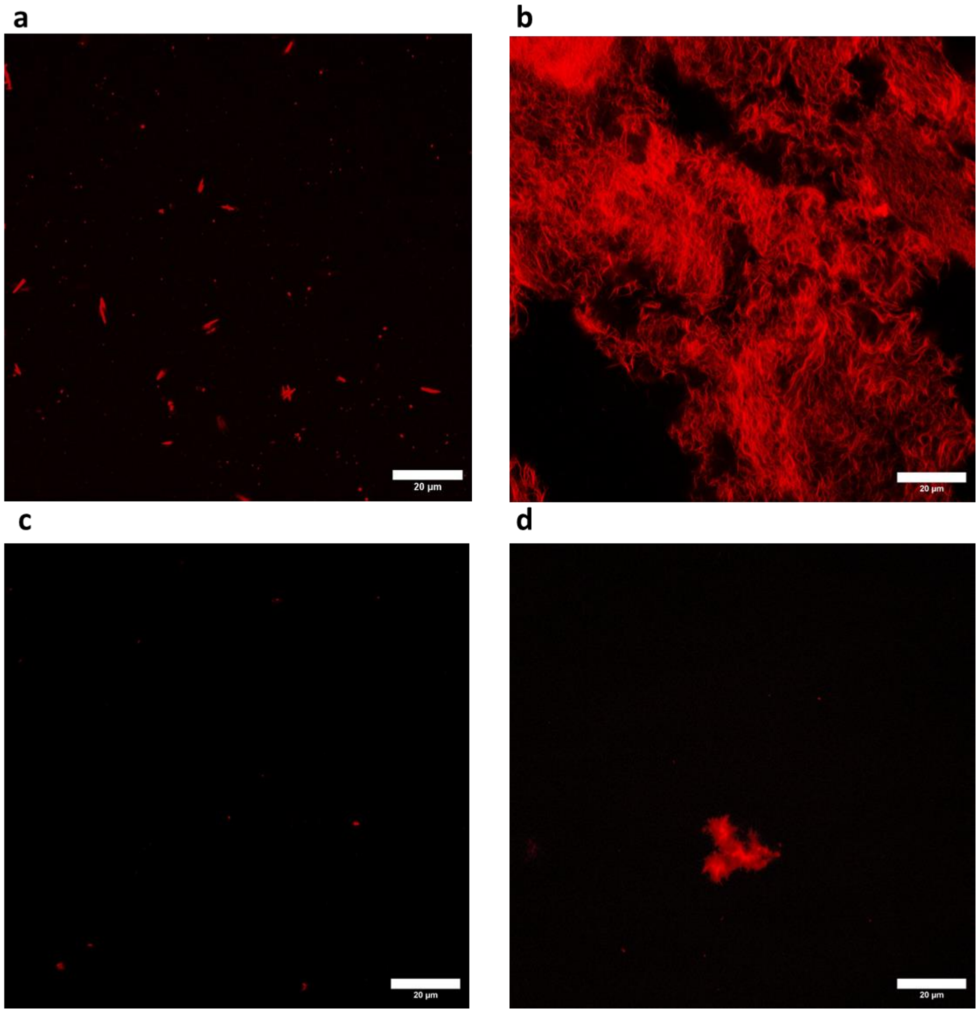

Figure S17: Confocal images of the reaction of $Z^{L/D}$VEP (10 mM) with AcCY (10 mM) in borate buffer (0.6 M, pH 9.1) after a, c) 5 h and b, d) 3 days reaction time. a) and b) show the left-handed system while c) and d) show the right-handed system. All scale bars represent 20 µm. The samples were stained with 1 µM Nile Red.

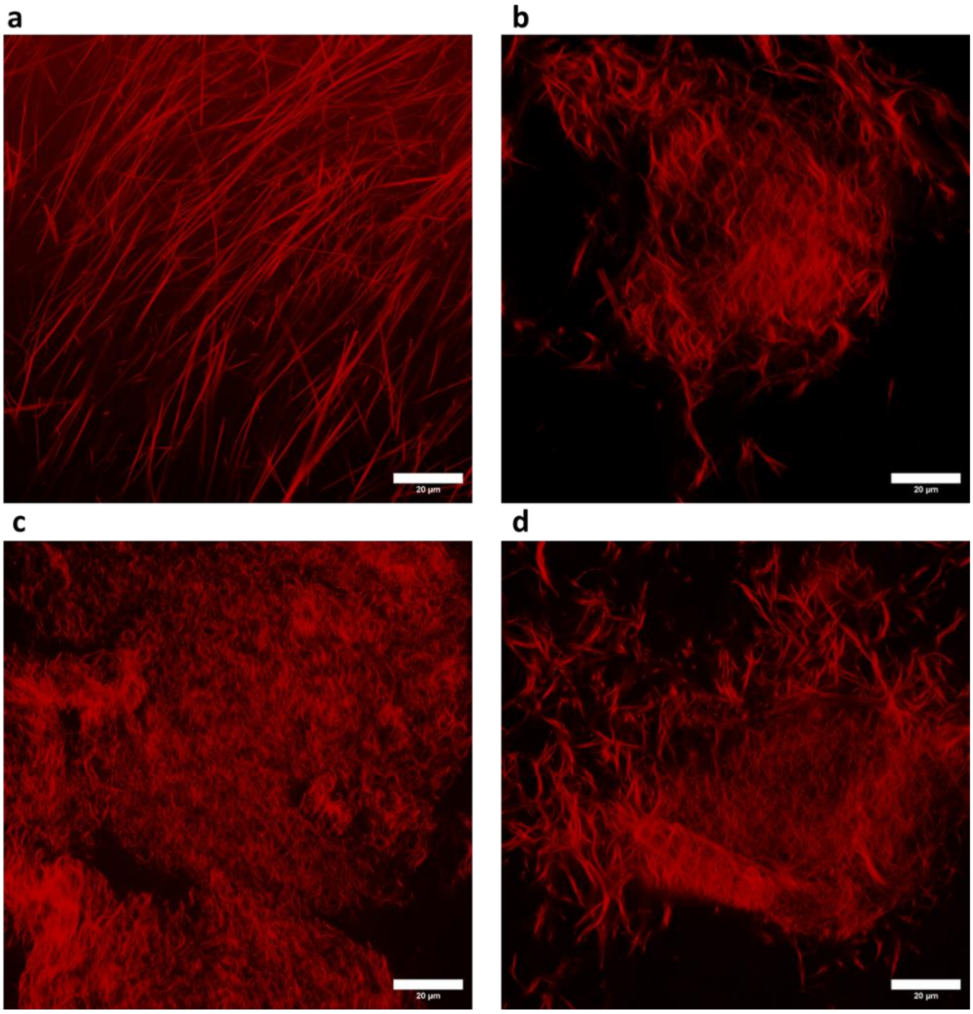

Figure S18: Confocal images of the reaction of AcCF (10 mM) in borate buffer (0.6 M, pH 9.1) with a) Z^{L}VEP (10 mM), b) Z^{D}VEP (10 mM), c) Z^{L}FEP (10 mM) and d) Z^{D}FEP (10 mM) after 2 days reaction time. All scale bars represent 20 µm. The samples were stained with 1 µM Nile Red.

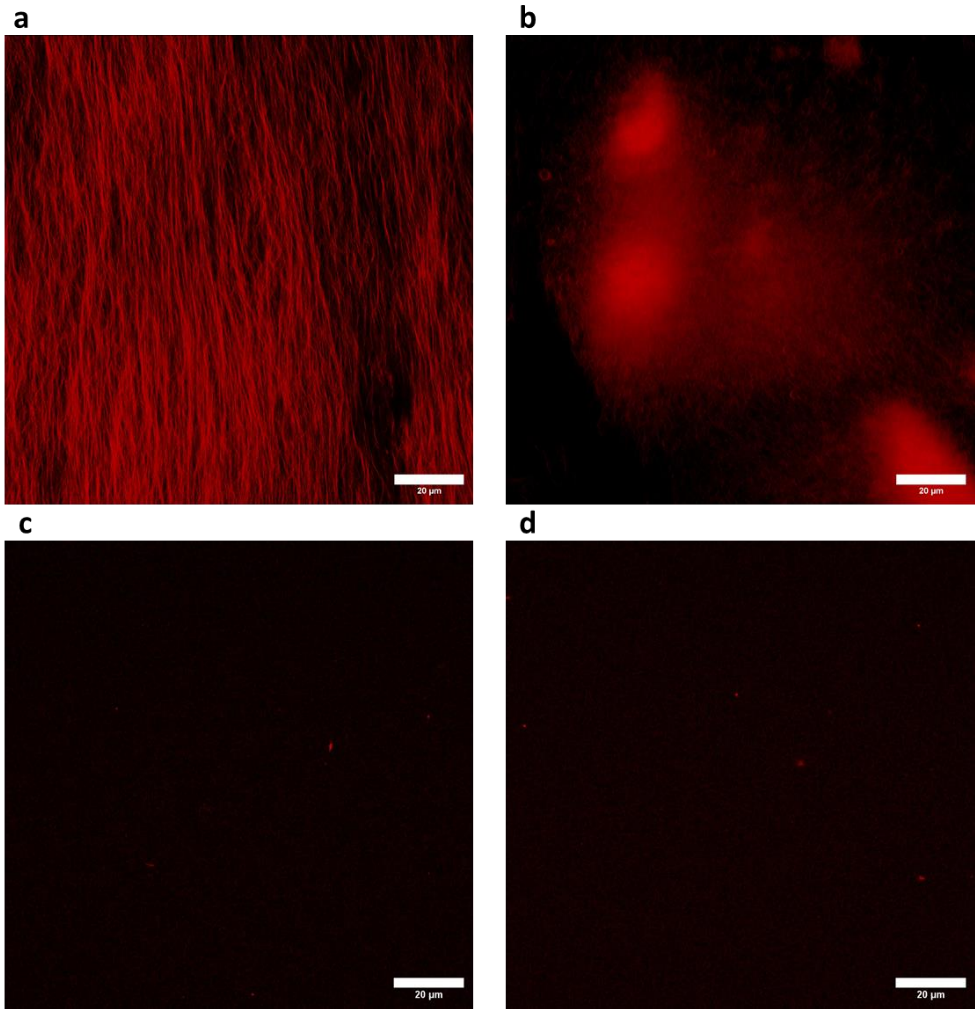

Figure S19: Confocal images of the reaction of a) Z^{L}FEP (10 mM) or b) Z^{D}FEP (10 mM) with AcCHF (10 mM) in borate buffer (0.6 M, pH 9.1) after 2 days reaction time and confocal images of the reaction of c) Z^{L}FEP (10 mM) or d) Z^{D}FEP (10 mM) with AcYD (20 mM) in bicarbonate buffer (0.2 M, pH 10.1) after 30 min reaction time. All scale bars represent 20 µm. The samples were stained with 1 µM Nile Red.

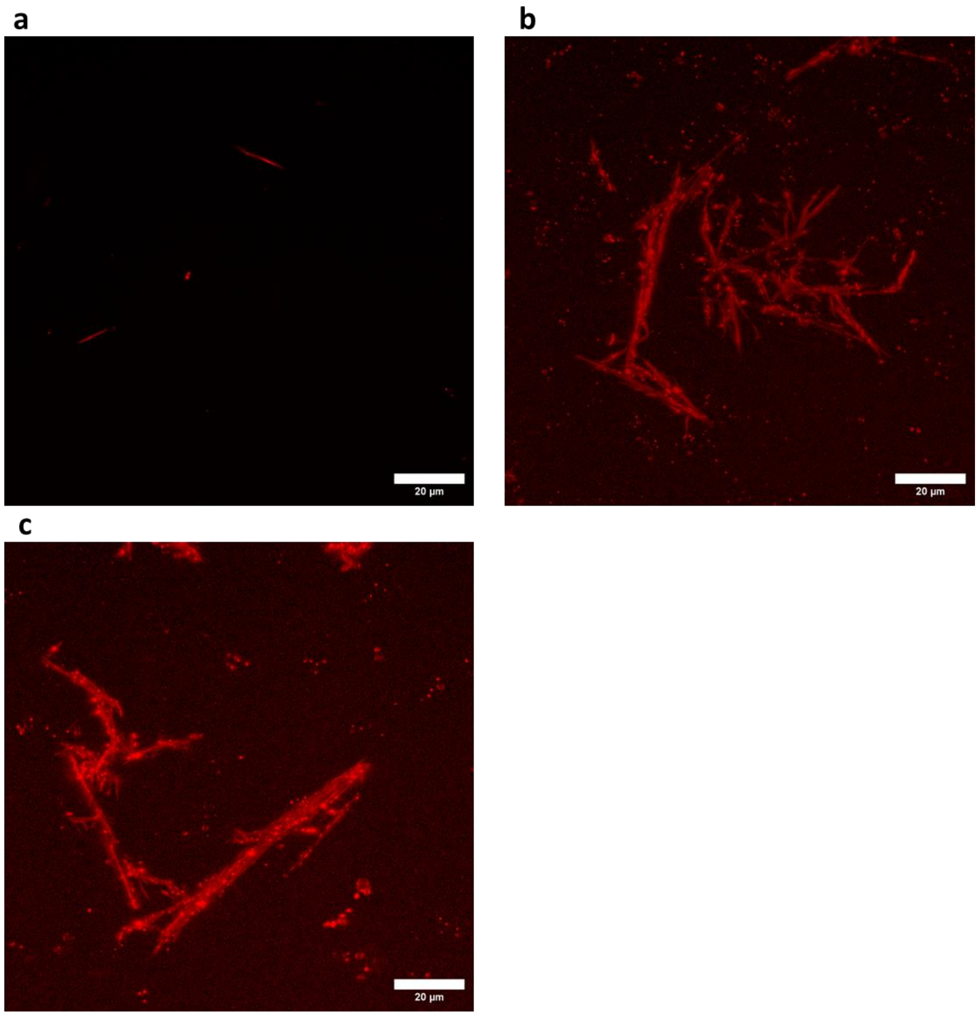

Figure S20: Confocal images of the reaction of mixtures of Z^{L}VEP and Z^{D}VEP with L:D-ratios of a) 75:25, b) 50:50 and c) 25:75 (total $Z^{L/D}$VEP concentration of 10 mM) with AcYF (20 mM) in bicarbonate buffer (0.2 M, pH 10.1) after one day reaction time. All scale bars represent 20 µm. The sample for confocal microscopy was stained with 1 µM Nile Red.

6.5 Macroscopic appearance

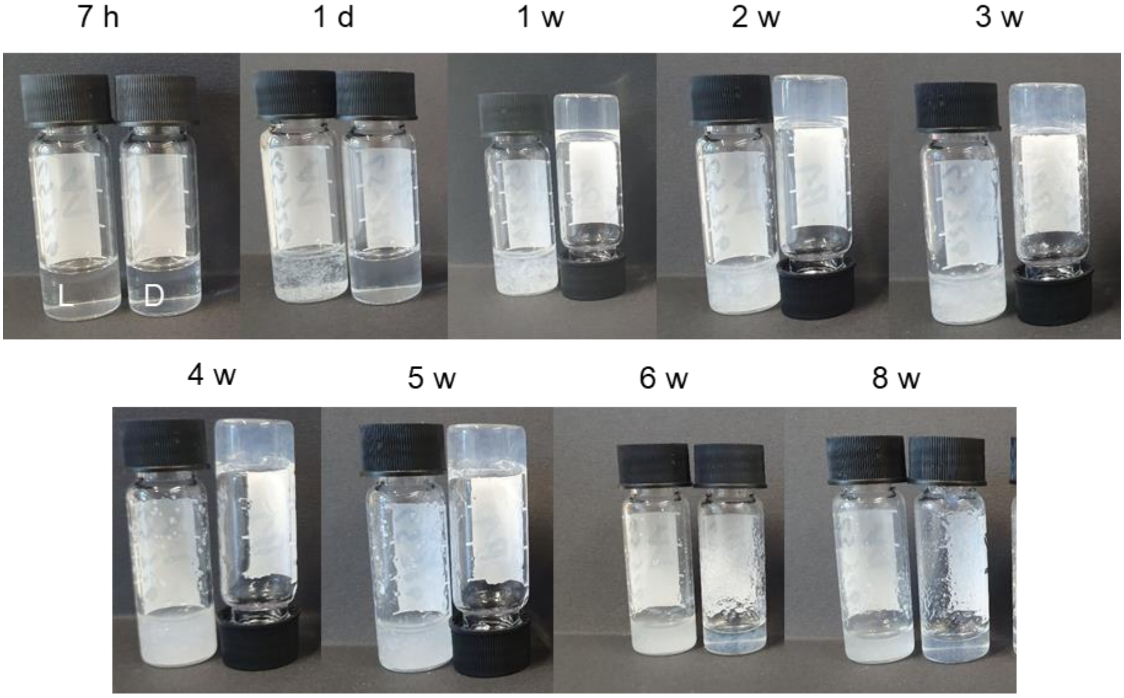

Figure S21: Images of vials containing the reaction of $Z^{L/D}$VEP (10 mM) with AcCF (10 mM) in borate buffer (0.6 M, pH 9.1) over time. The vials on the left contain the left-handed acylating agent, whereas the vials on the right contain the right-handed acylating agent. Time units are abbreviated for clarity: hours (h), days (d) and weeks (w).

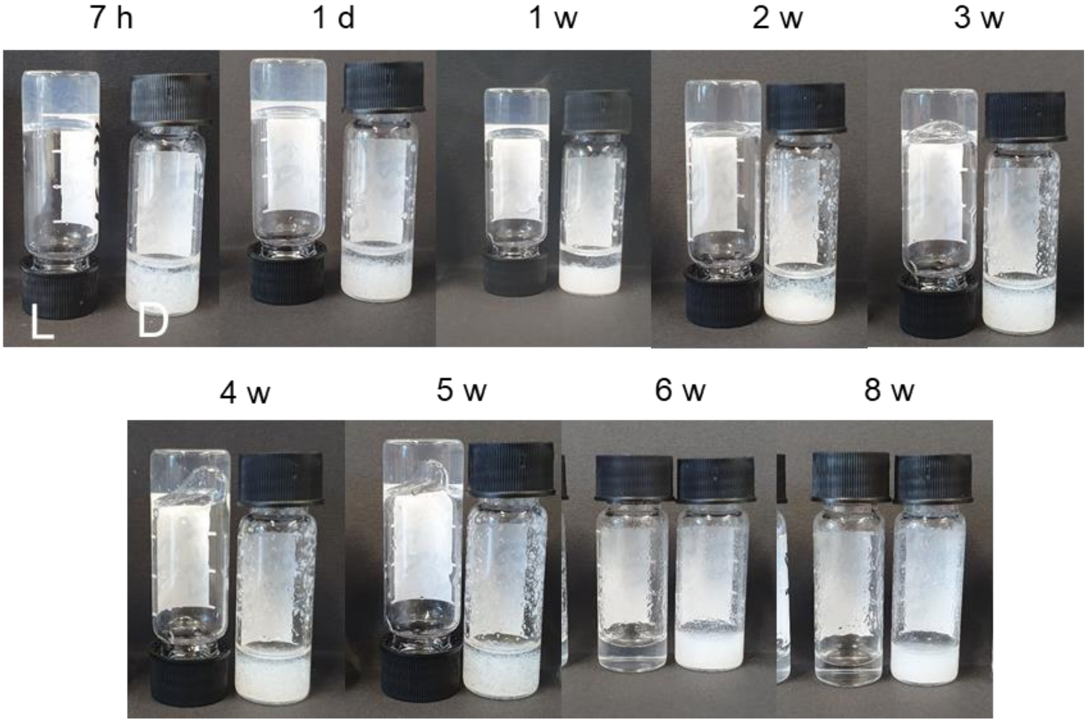

Figure S22: Images of vials containing the reaction of $Z^{L/D}$VEP (10 mM) with AcCHF (10 mM) in borate buffer (0.6 M, pH 9.1) over time. The vials on the left contain the left-handed acylating agent, whereas the vials on the right contain the right-handed acylating agent. Time units are abbreviated for clarity: hours (h), days (d) and weeks (w).

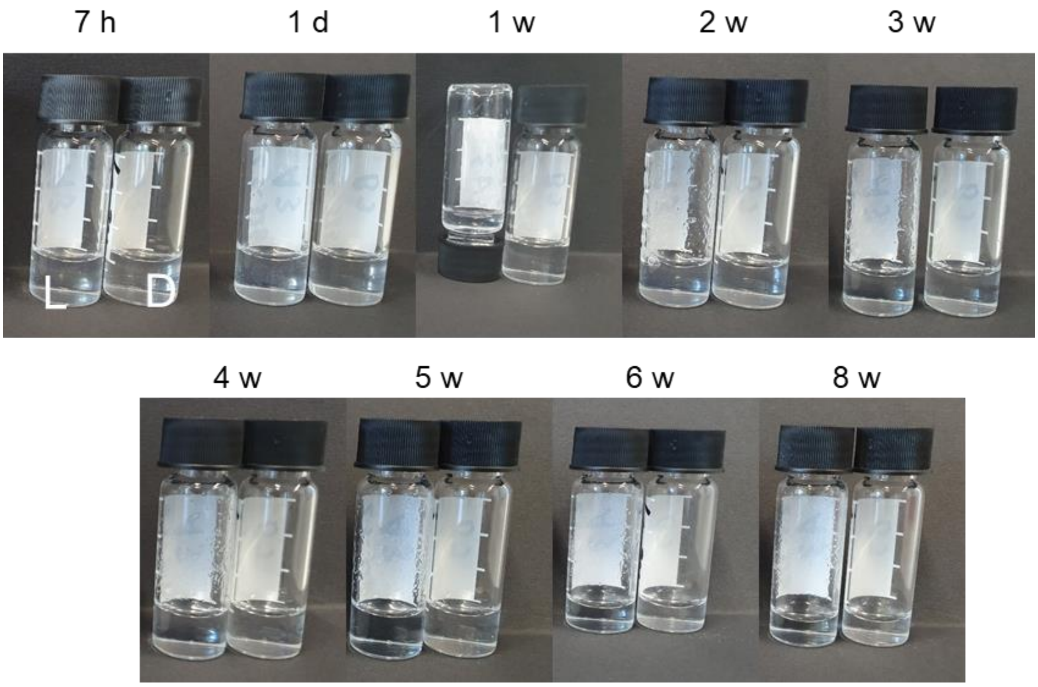

Figure S23: Images of vials containing the reaction of $Z^{L/D}$VEP (10 mM) with AcCY (10 mM) in borate buffer (0.6 M, pH 9.1) over time. The vials on the left contain the left-handed acylating agent, whereas the vials on the right contain the right-handed acylating agent. Time units are abbreviated for clarity: hours (h), days (d) and weeks (w).

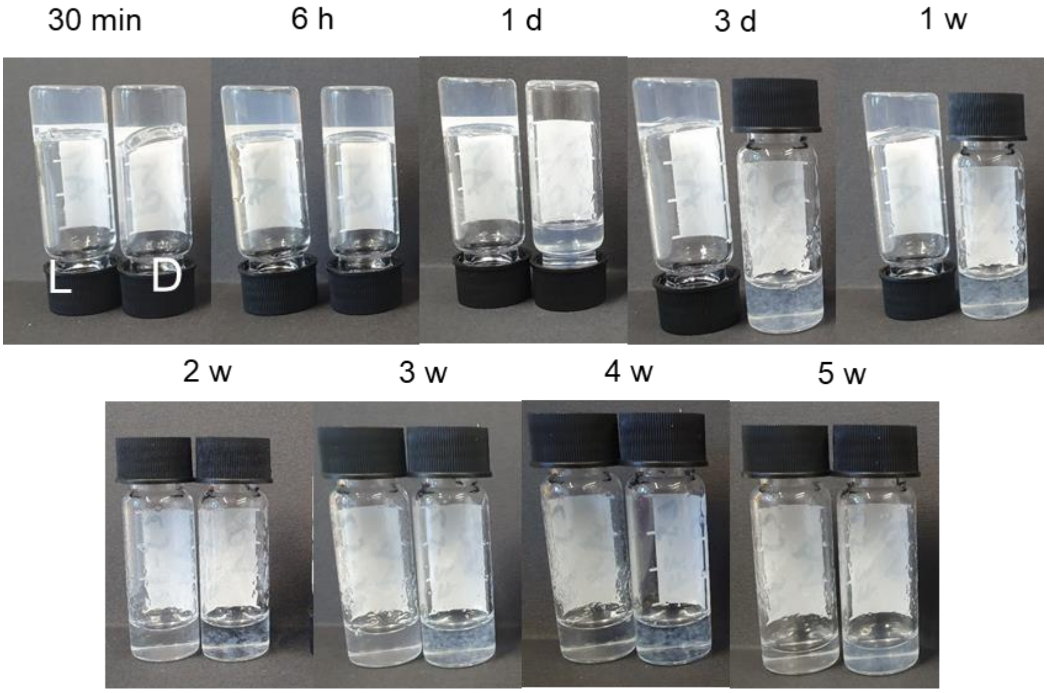

Figure S24: Images of vials containing the reaction of $Z^{L/D}$FEP (10 mM) with AcCF (10 mM) in borate buffer (0.6 M, pH 9.1) over time. The vials on the left contain the left-handed acylating agent, whereas the vials on the right contain the right-handed acylating agent. Time units are abbreviated for clarity: hours (h), days (d) and weeks (w).

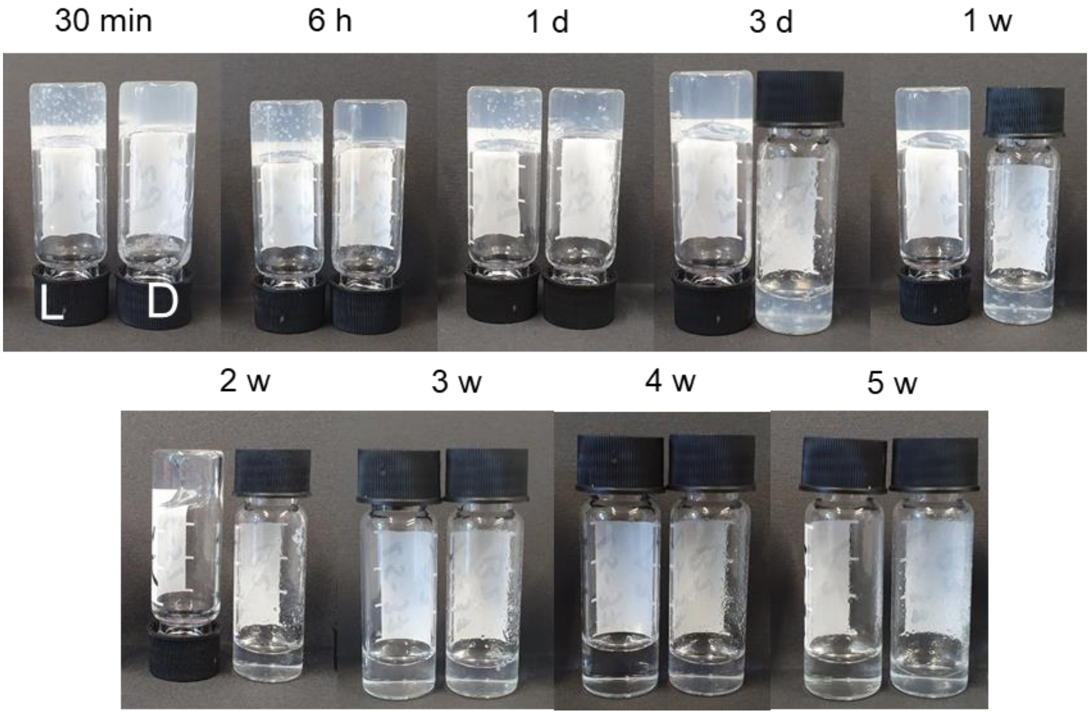

Figure S25: Images of vials containing the reaction of $Z^{L/D}$FEP (10 mM) with AcCHF (10 mM) in borate buffer (0.6 M, pH 9.1) over time. The vials on the left contain the left-handed acylating agent, whereas the vials on the right contain the right-handed acylating agent. Time units are abbreviated for clarity: hours (h), days (d) and weeks (w).

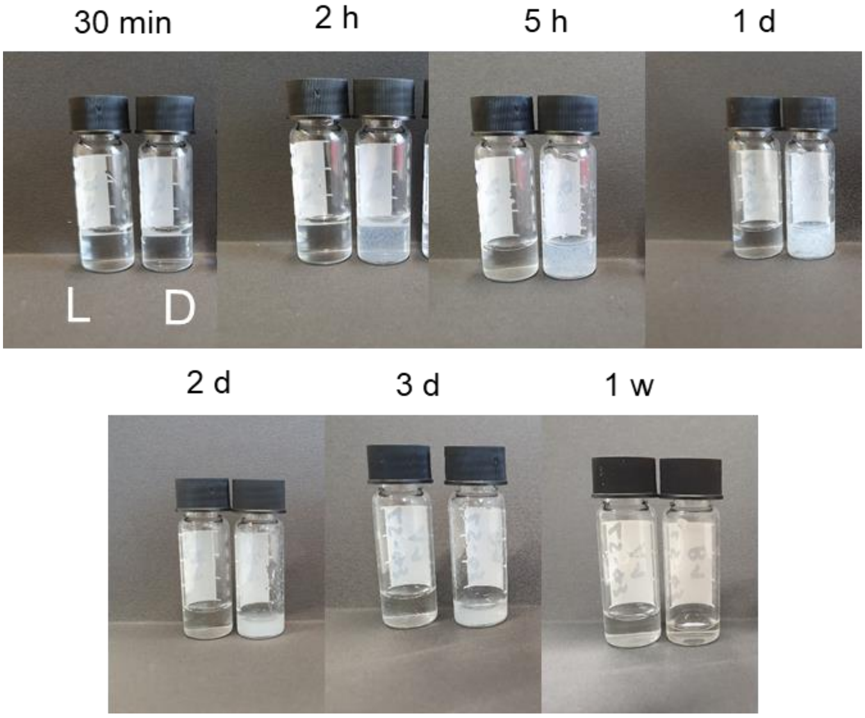

Figure S26: Images of vials containing the reaction of $Z^{L/D}$VEP (10 mM) with AcYF (20 mM) in bicarbonate buffer (0.2 M, pH 10.1) over time. The vials on the left contain the left-handed acylating agent, whereas the vials on the right contain the right-handed acylating agent. Time units are abbreviated for clarity: hours (h), days (d) and weeks (w).

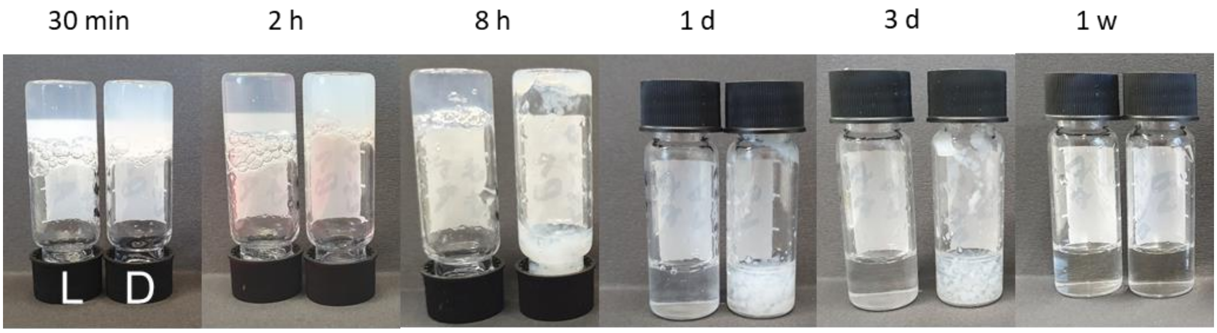

Figure S27: Images of vials containing the reaction of Z$^{L/D}$FEP (10 mM) with AcYF (20 mM) in bicarbonate buffer (0.2 M, pH 10.1) over time. The vials on the left contain the left-handed acylating agent, whereas the vials on the right contain the right-handed acylating agent. Time units are abbreviated for clarity: hours (h), days (d) and weeks (w).

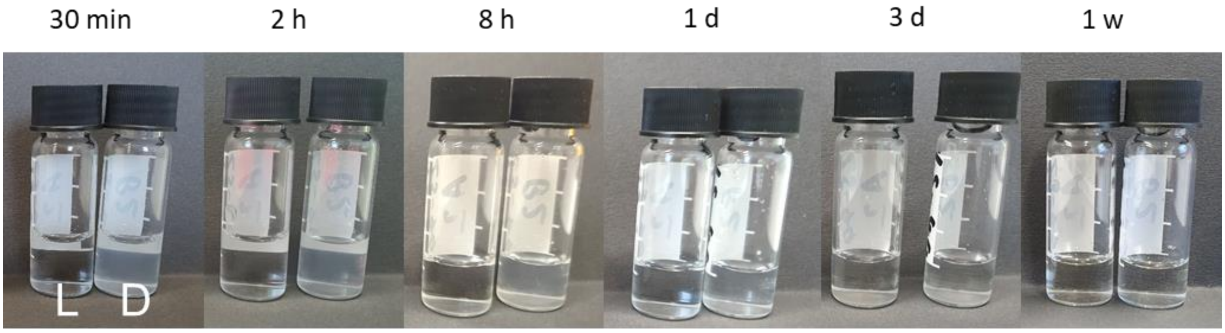

Figure S28: Images of vials containing the reaction of Z$^{L/D}$FEP (10 mM) with AcYH (20 mM) in bicarbonate buffer (0.2 M, pH 10.1) over time. The vials on the left contain the left-handed acylating agent, whereas the vials on the right contain the right-handed acylating agent. Time units are abbreviated for clarity: hours (h), days (d) and weeks (w).

6.6 Plots of concentration over time

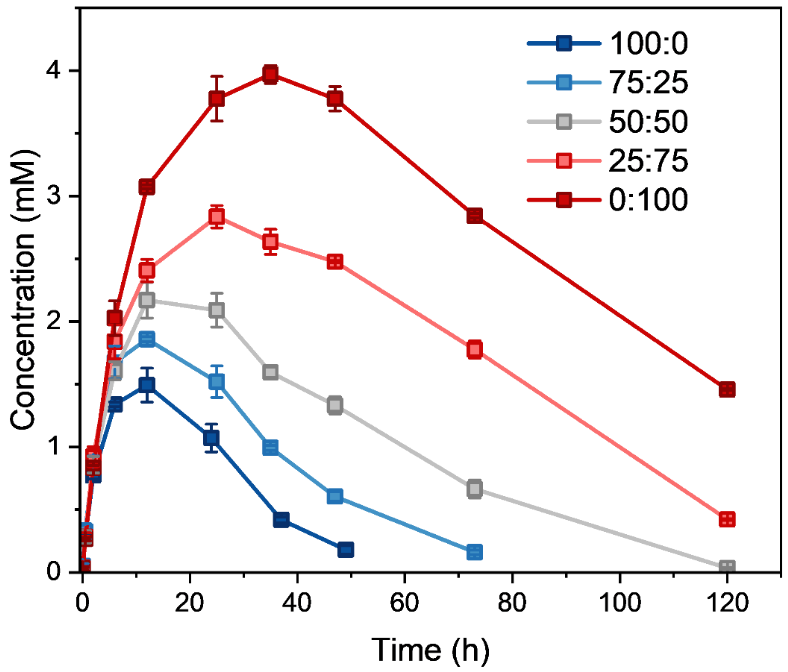

Figure S29: Plot of ester concentration over time for the reaction of mixtures of Z^{L}VEP and Z^{D}VEP with different L:D-ratios (total Z$^{L/D}$VEP concentration of 10 mM) with AcYF (20 mM) in bicarbonate buffer (0.2 M, pH 10.1) obtained from HPLC measurements using method 1.

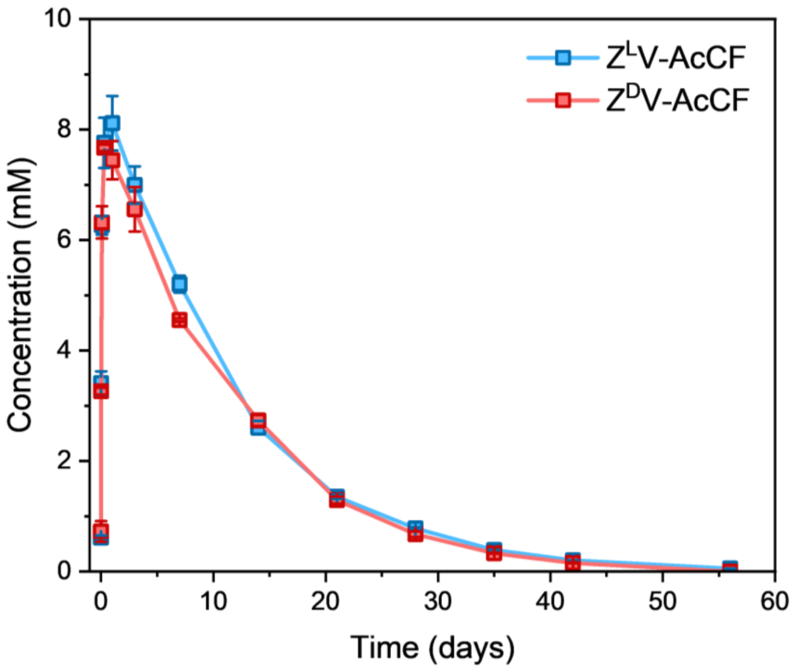

Figure S30: Plot of thioester concentration over time for the reaction of $Z^{L/D}$VEP (10 mM) with AcCF (10 mM) in borate buffer (0.6 M, pH 9.1) obtained from HPLC measurements using method 1.

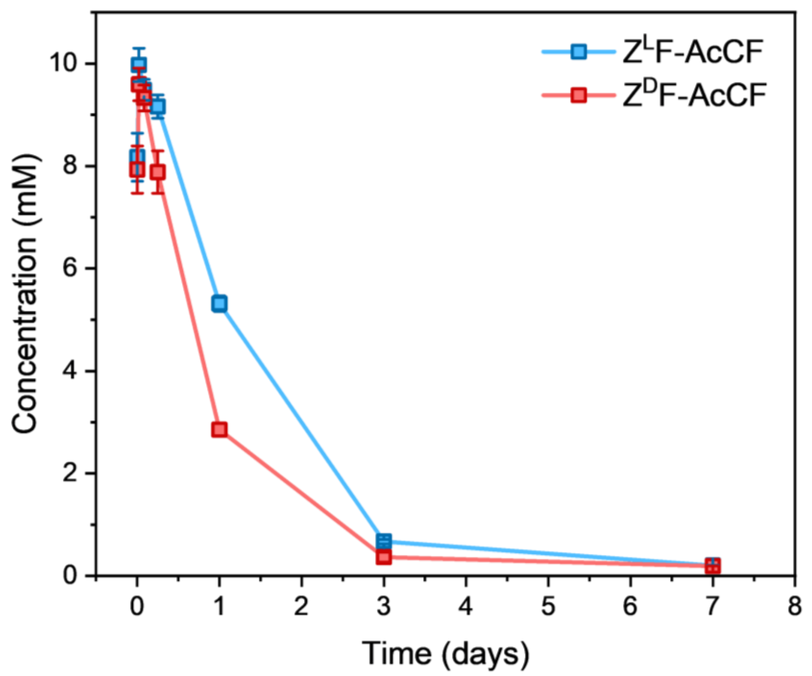

Figure S31: Plot of thioester concentration over time for the reaction of $Z^{L/D}$FEP (10 mM) with AcCF (10 mM) in borate buffer (0.6 M, pH 9.1) obtained from HPLC measurements using method 1.

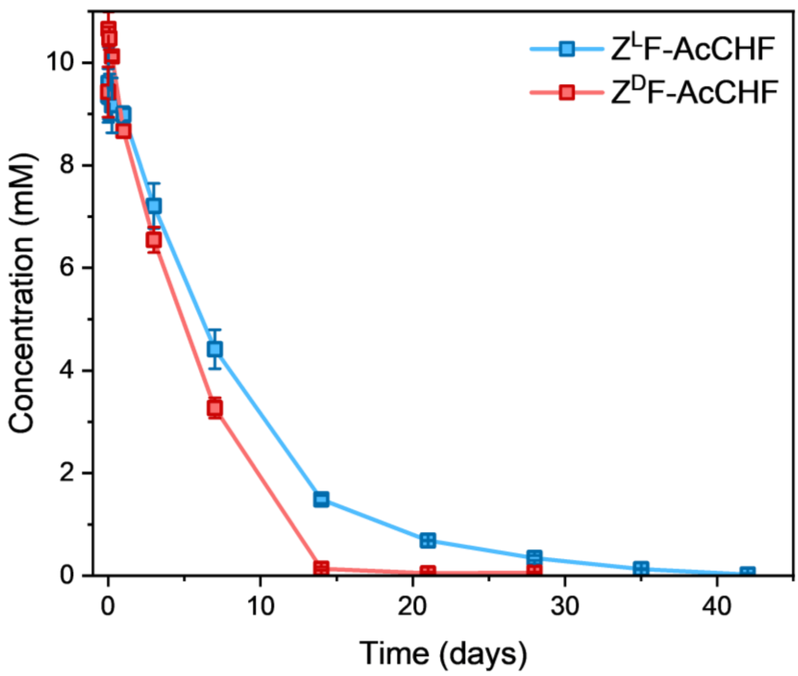

Figure S32: Plot of thioester concentration over time for the reaction of $Z^{L/D}$FEP (10 mM) with AcCHF (10 mM) in borate buffer (0.6 M, pH 9.1) obtained from HPLC measurements using method 1.

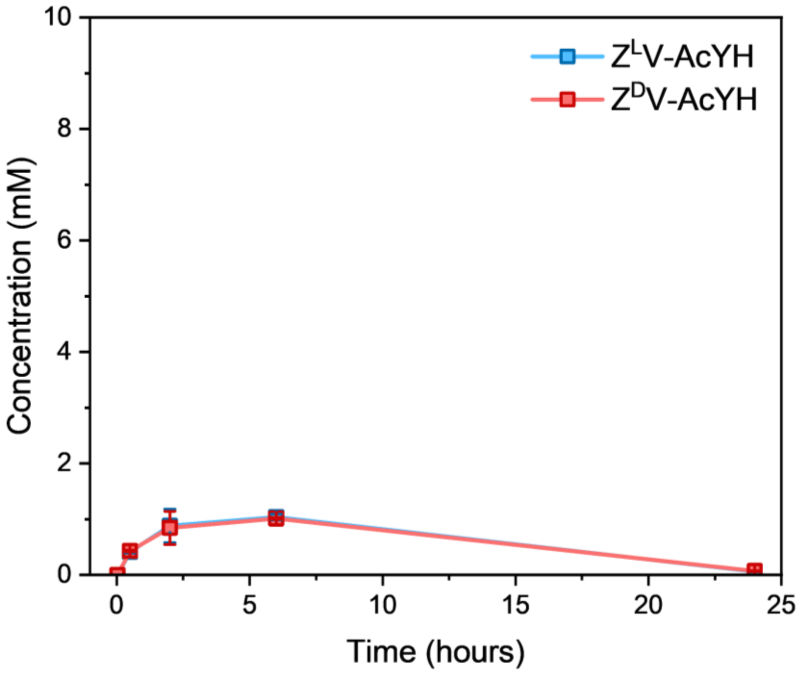

Figure S33: Plot of ester concentration over time for the reaction of $Z^{L/D}$VEP (10 mM) with AcYH (20 mM) in bicarbonate buffer (0.2 M, pH 10.1) obtained from HPLC measurements using method 1.

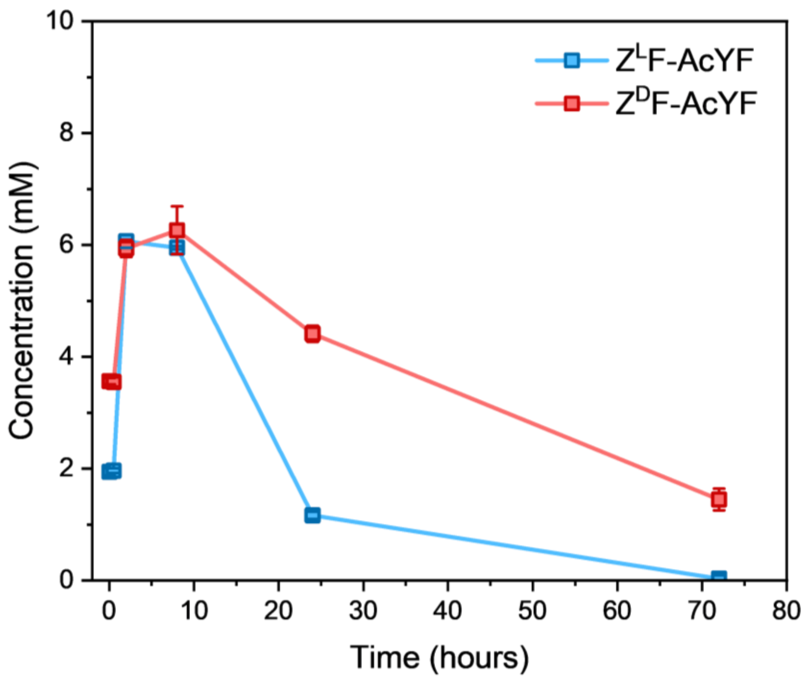

Figure S34: Plot of ester concentration over time for the reaction of $Z^{L/D}$FEP (10 mM) with AcYF (20 mM) in bicarbonate buffer (0.2 M, pH 10.1) obtained from HPLC measurements using method 1.

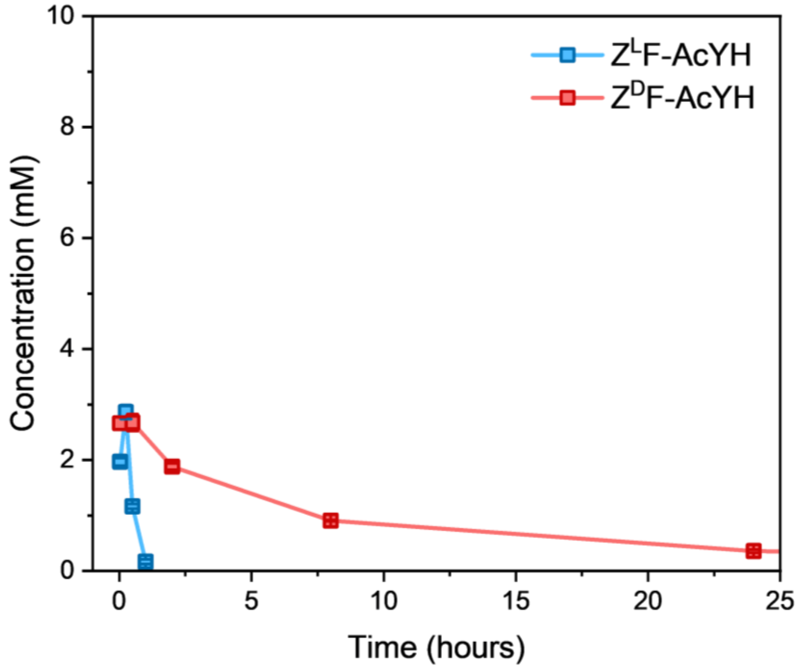

Figure S35: Plot of ester concentration over time for the reaction of $Z^{L/D}$FEP (10 mM) with AcYH (20 mM) in bicarbonate buffer (0.2 M, pH 10.1) obtained from HPLC measurements using method 1.

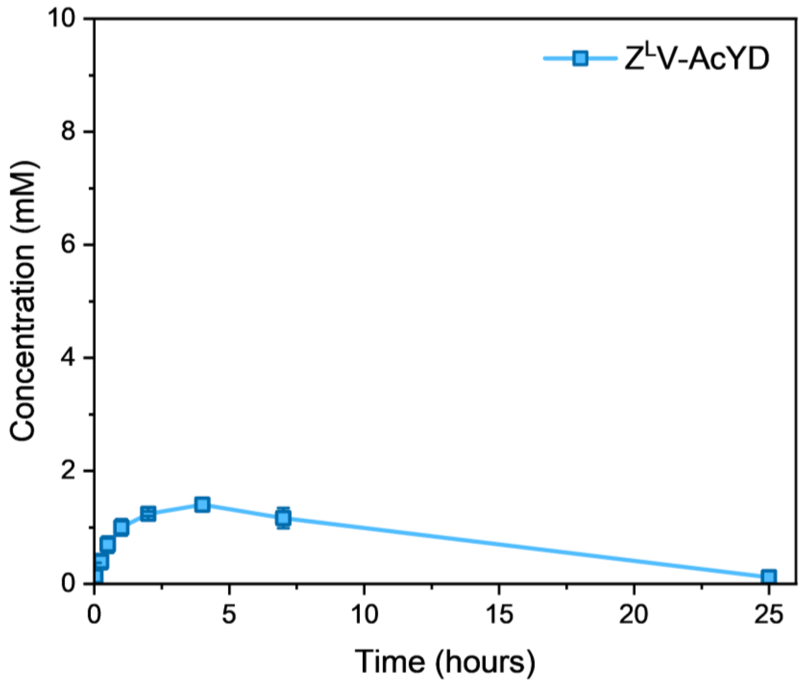

Figure S36: Plot of ester concentration over time for the reaction of $Z^{L/D}$VEP (10 mM) with AcYD (20 mM) in bicarbonate buffer (0.2 M, pH 10.1) obtained from HPLC measurements using method 1.

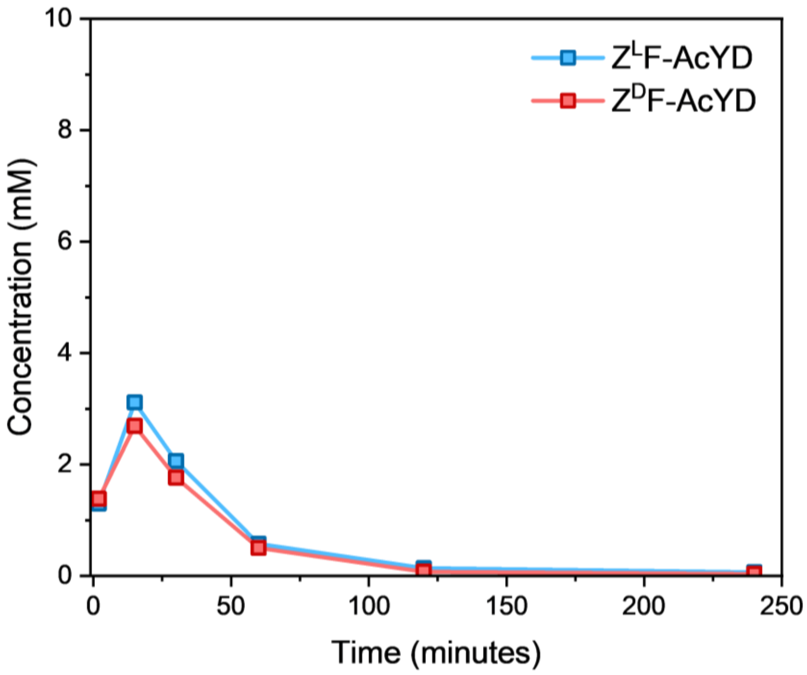

Figure S37: Plot of ester concentration over time for the reaction of $Z^{L/D}$FEP (10 mM) with AcYD (20 mM) in bicarbonate buffer (0.2 M, pH 10.1) obtained from HPLC measurements using method 1.

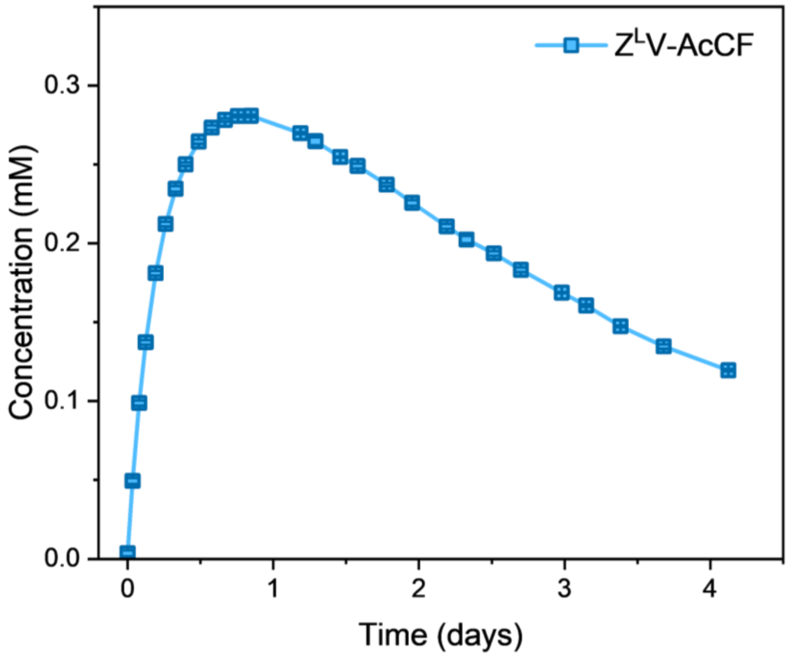

Figure S38: Plot of ester concentration over time for the reaction of Z^LVEP (0.5 mM) with AcCF (0.5 mM) in borate buffer (0.6 M, pH 9.1) obtained by injecting the reaction mixture without dilution to HPLC measurements using method 1.

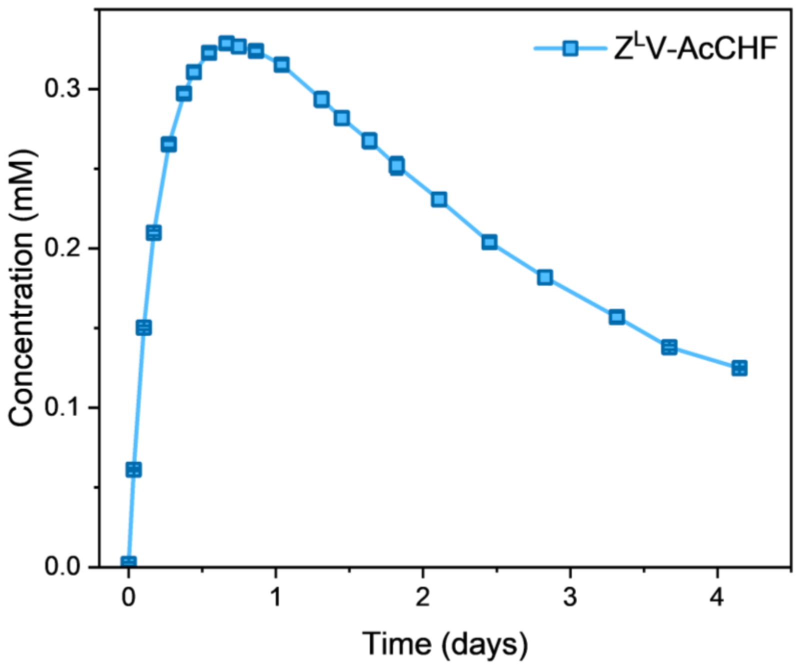

Figure S39: Plot of ester concentration over time for the reaction of Z^{L}VEP (0.5 mM) with AcCHF (0.5 mM) in borate buffer (0.6 M, pH 9.1) obtained by injecting the reaction mixture without dilution to HPLC measurements using method 1.

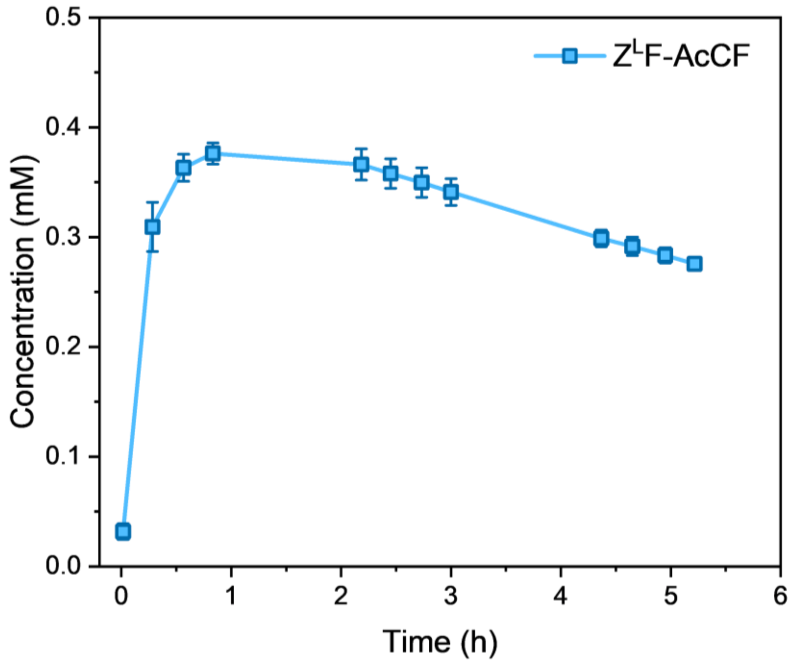

Figure S40: Plot of ester concentration over time for the reaction of Z^LFEP (0.5 mM) with AcCF (0.5 mM) in borate buffer (0.6 M, pH 9.1) obtained by injecting the reaction mixture without dilution to HPLC measurements using method 1.

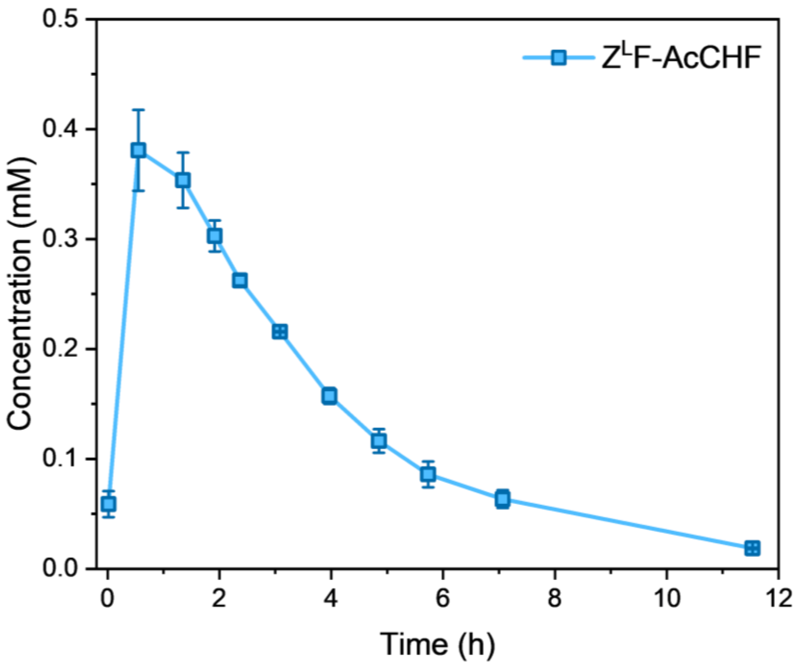

Figure S41: Plot of ester concentration over time for the reaction of Z^{L}FEP (0.5 mM) with AcCHF (0.5 mM) in borate buffer (0.6 M, pH 9.1) obtained by injecting the reaction mixture without dilution to HPLC measurements using method 1.

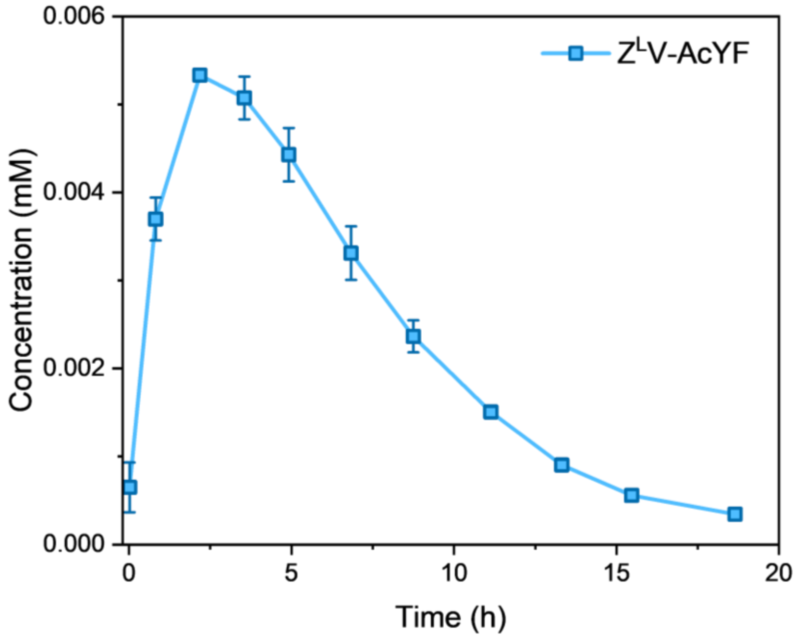

Figure S42: Plot of ester concentration over time for the reaction of Z^{L}VEP (0.5 mM) with AcYF (1 mM) in bicarbonate buffer (0.2 M, pH 10.1) obtained by injecting the reaction mixture without dilution to HPLC measurements using method 1.

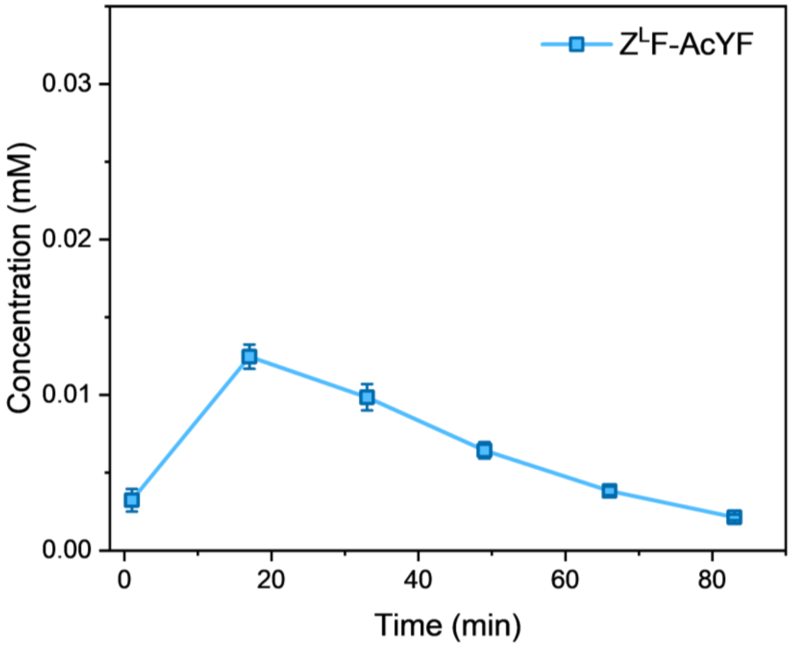

Figure S43: Plot of ester concentration over time for the reaction of Z[L]FEP (0.5 mM) with AcYF (1 mM) in bicarbonate buffer (0.2 M, pH 10.1) obtained by injecting the reaction mixture without dilution to HPLC measurements using method 1.

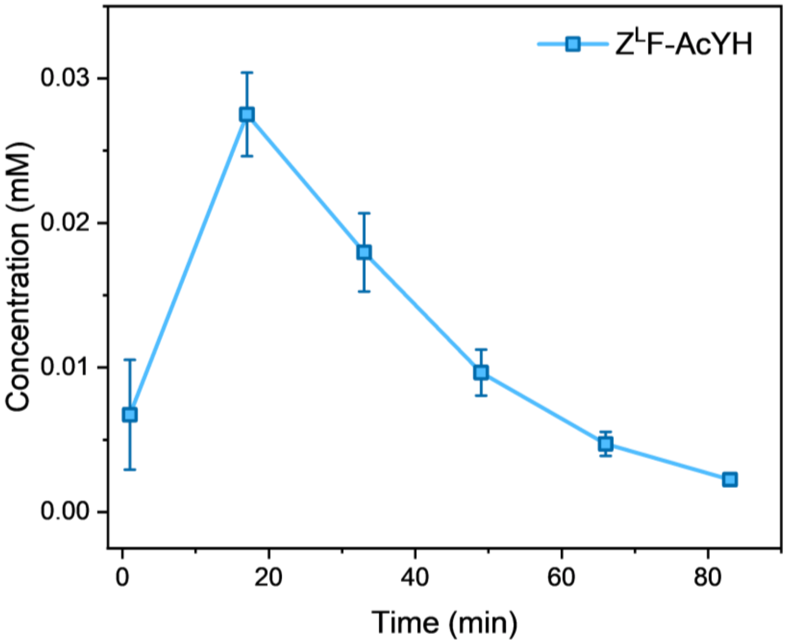

Figure S44: Plot of ester concentration over time for the reaction of Z^LFEP (0.5 mM) with AcYH (1 mM) in bicarbonate buffer (0.2 M, pH 10.1) obtained by injecting the reaction mixture without dilution to HPLC measurements using method 1.

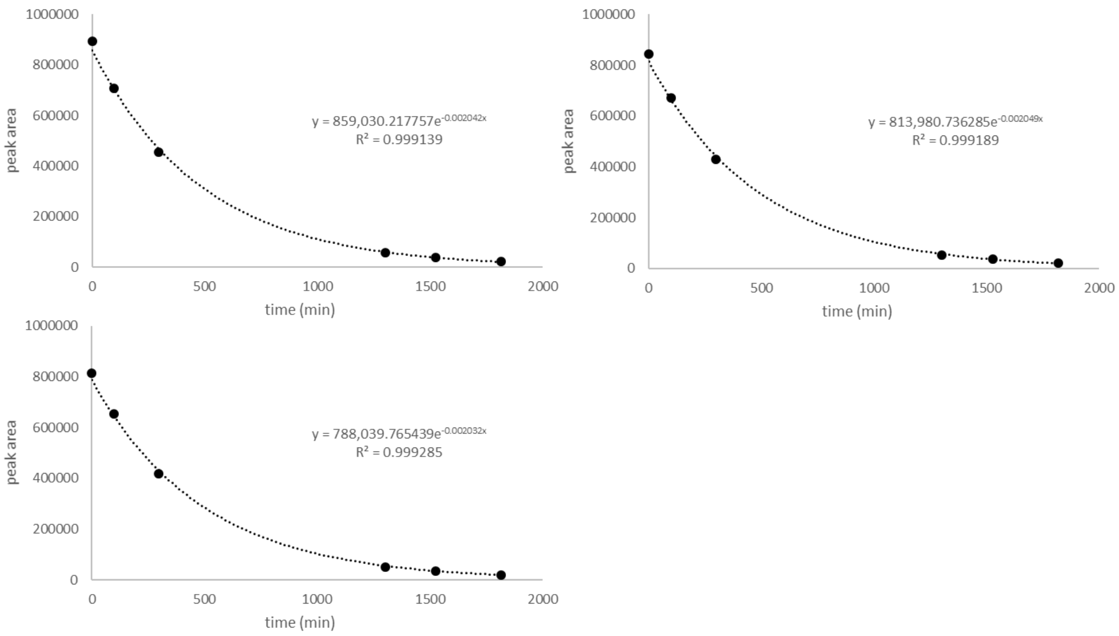

Figure S45: Experimental peak area over time for Z^LVEP in bicarbonate buffer (0.2 M, pH 10.1) obtained from HPLC method 1. Exponential fit gives an average k-value of 0.002041 with a standard deviation of 6.98727E-06 resulting in a half-lifetime of 5.6 h. Z^LFEP has a half lifetime of 0.5 h under these conditions. In borate buffer (0.6 M, pH 9.1), Z^LFEP has a half lifetime of 1.5 h compared to 29 h for Z^LVEP.[2,4]

6.7 Calibration curves

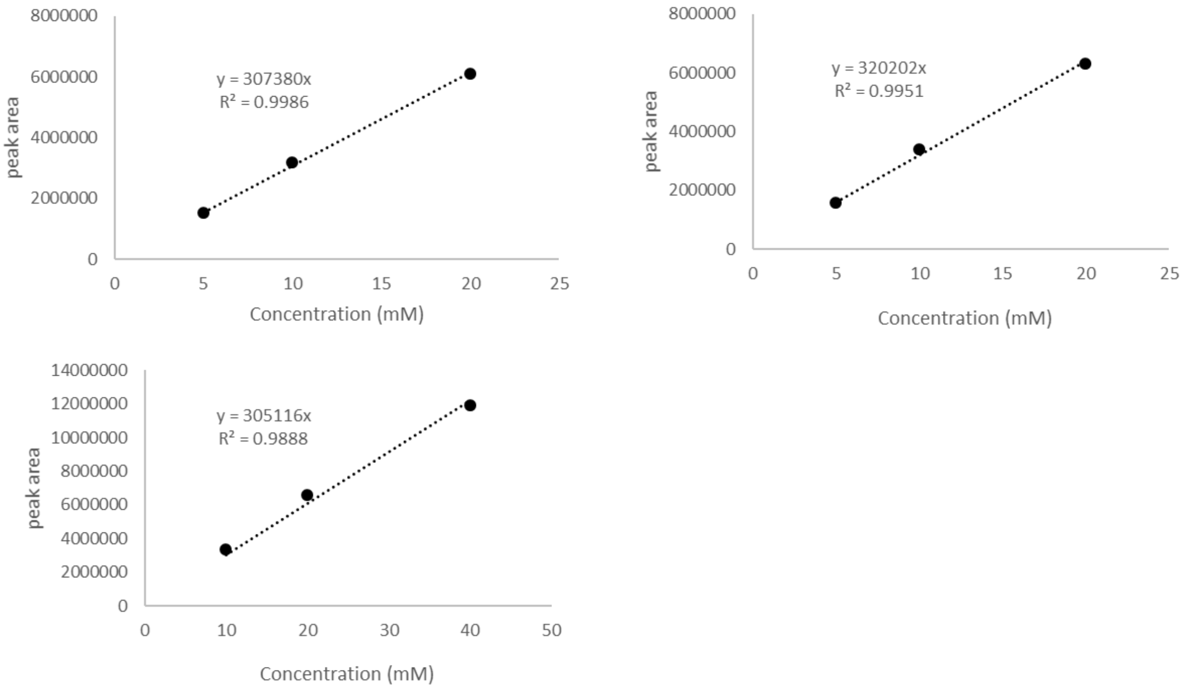

Figure S46: Calibration curves for Z^LF (average: 310899, standard deviation: 6642). The same value was used for Z^DF.

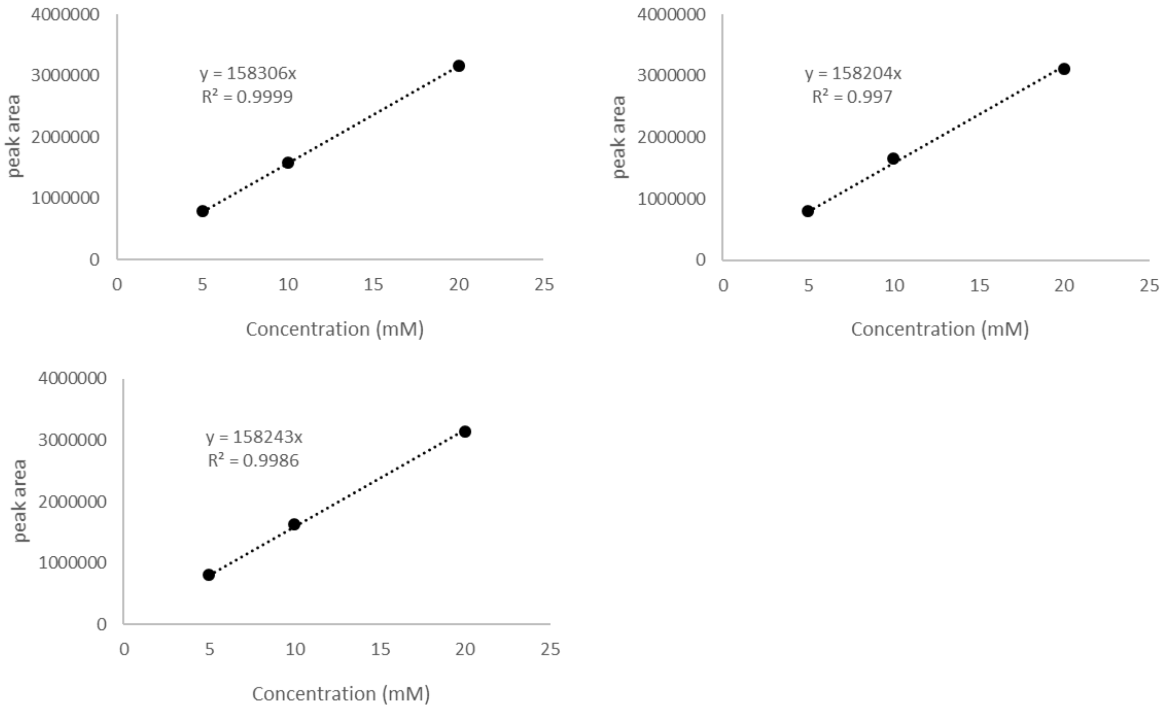

Figure S47: Calibration curves for Z^LV (average: 158251, standard deviation: 42). The same value was used for Z^DV.

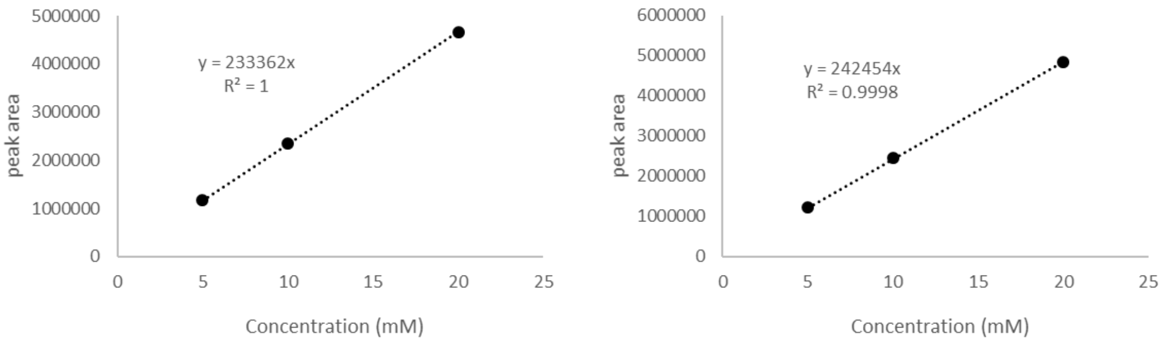

Figure S48: Calibration curves for AcCF (average: 237908, standard deviation: 4546).

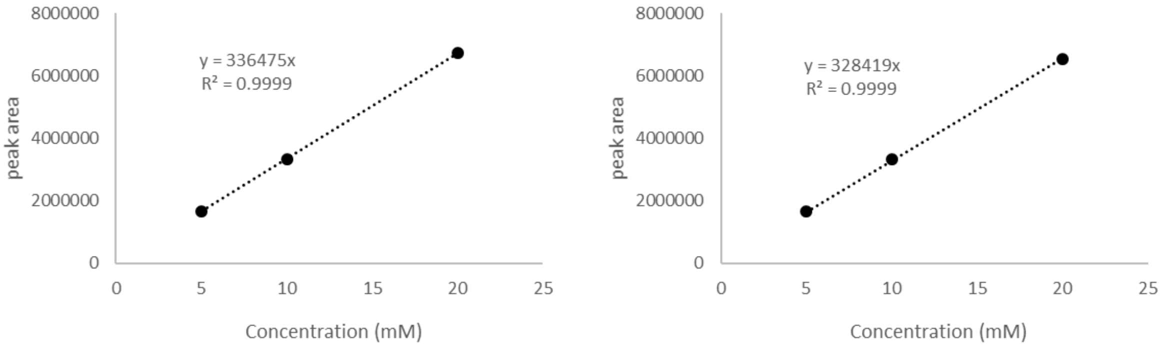

Figure S49: Calibration curves for AcCHF (average: 332447, standard deviation: 4028).

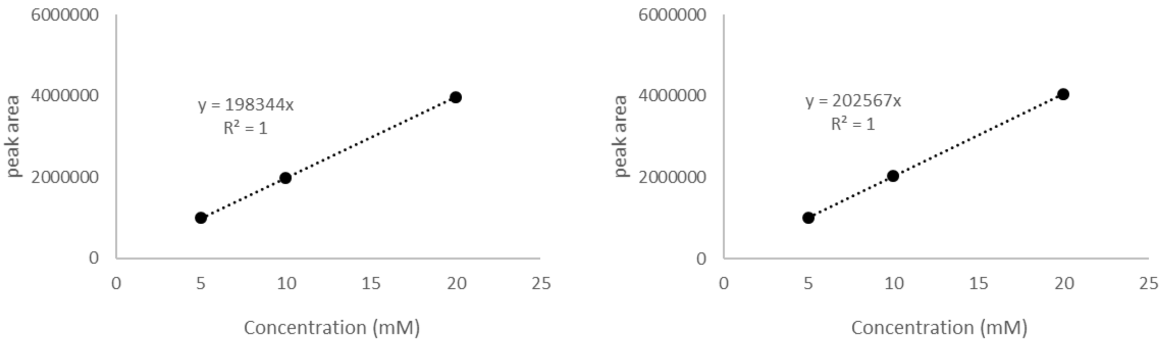

Figure S50: Calibration curves for AcCY (average: 200456, standard deviation: 2112).

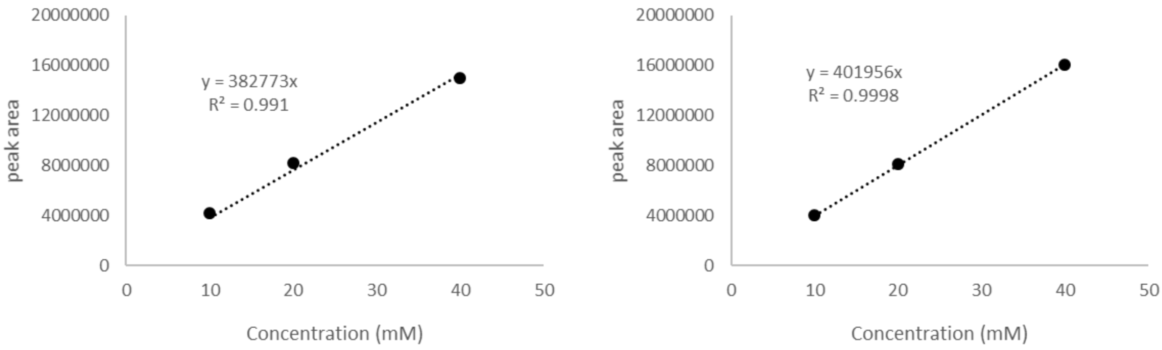

Figure S51: Calibration curves for AcYF (average: 392364, standard deviation: 9591).

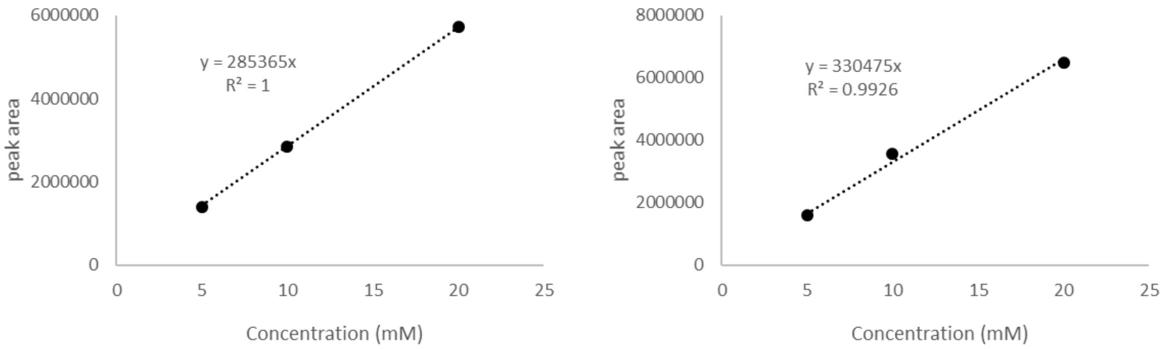

Figure S52: Calibration curves for AcYH (average: 307920, standard deviation: 22555).

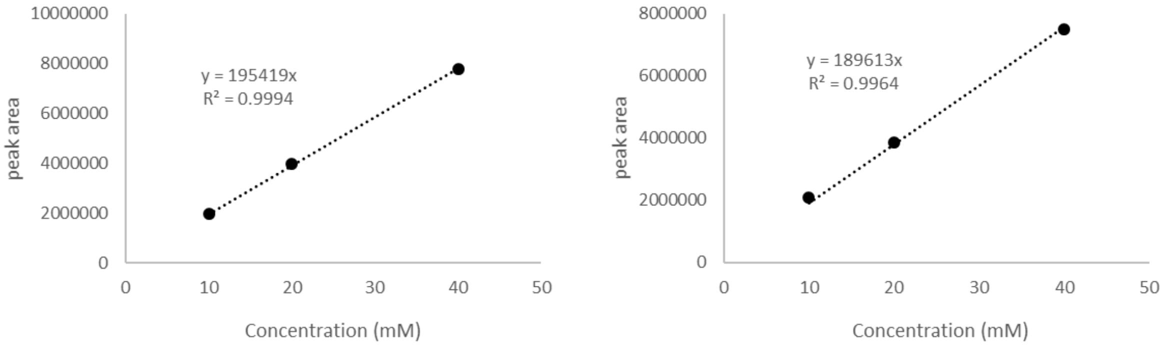

Figure S53: Calibration curves for AcYD (average: 194411, standard deviation: 4798).

6.8 LC-MS analysis

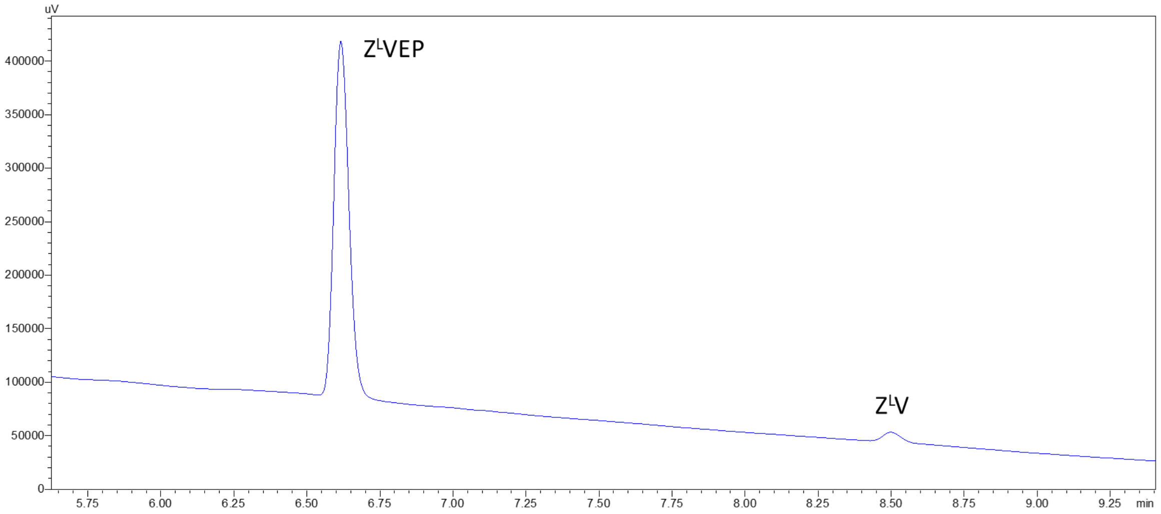

Figure S54: Chromatogram obtained from method 1 for Z^{L}VEP and its hydrolysis product Z^{L}V (4% peak area).

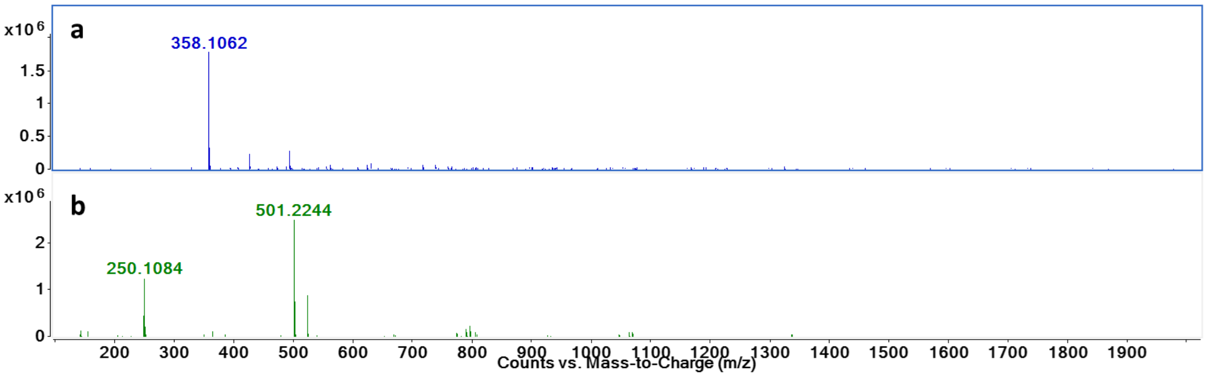

Figure S55: Mass spectra of a) Z^{L}VEP (retention time 6.61min, m/z $[M+H]^{-}$ calculated: 358.1061, found: 358.1062), b) Z^{L}V (retention time 8.49 min, m/z $[M]^{-}$ calculated: 250.1084, found: 250.1084, m/z $[2M]^{-}$ calculated: 501.2242, found: 501.2244). Retention times are similar for the Z^{D}VEP, and masses are identical.

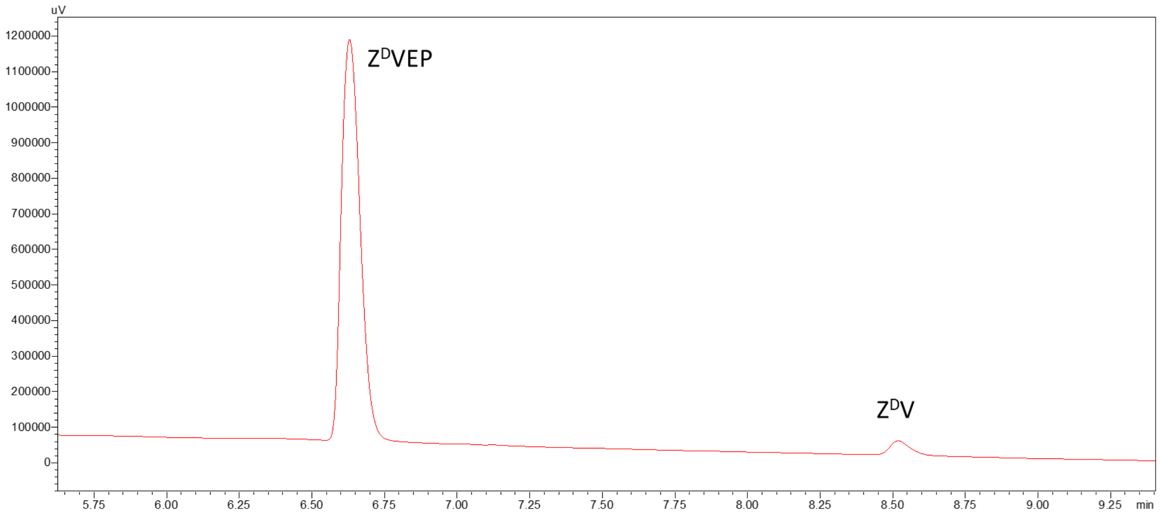

Figure S56: Chromatogram obtained from method 1 for $Z^{D}VEP$ and its hydrolysis product $Z^{D}V$ (4% peak area).

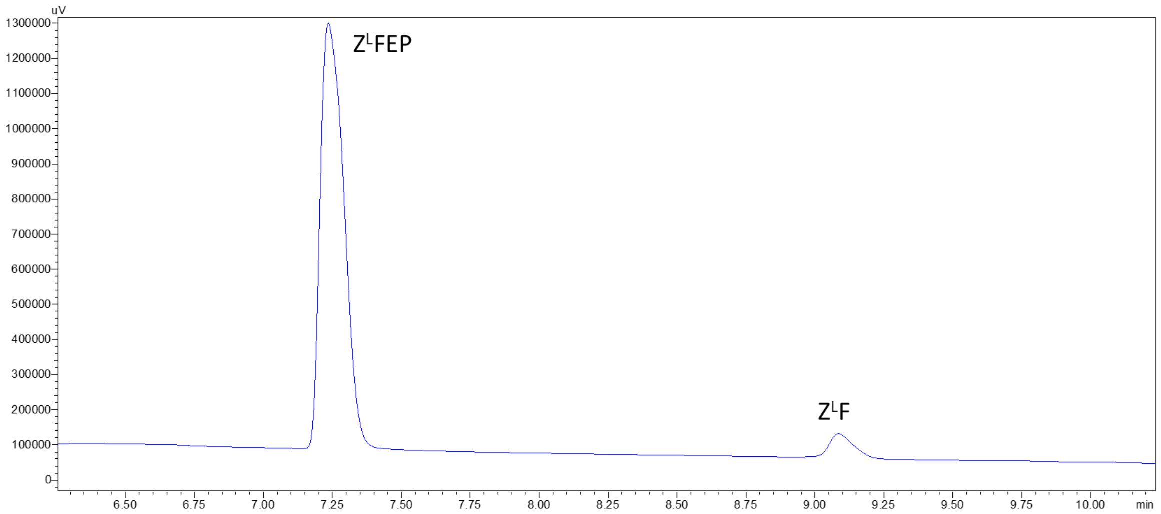

Figure S57: Chromatogram obtained from method 1 for Z^{L}FEP and its hydrolysis product Z^{L}F (6% peak area).

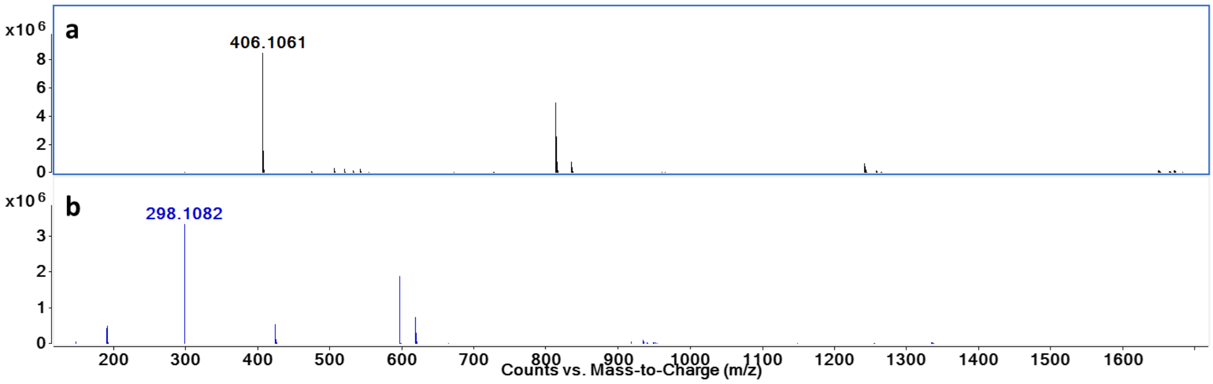

Figure S58: Mass spectra of a) Z^{L}FEP (retention time 7.20 min, m/z $[M+H]^-$ calculated: 406.1061, found: 406.1061), b) Z^{L}F (retention time 9.09 min, m/z $[M]^-$ calculated: 298.1084, found: 298.1082). Retention times are similar for the Z^{D}FEP, and masses are identical.

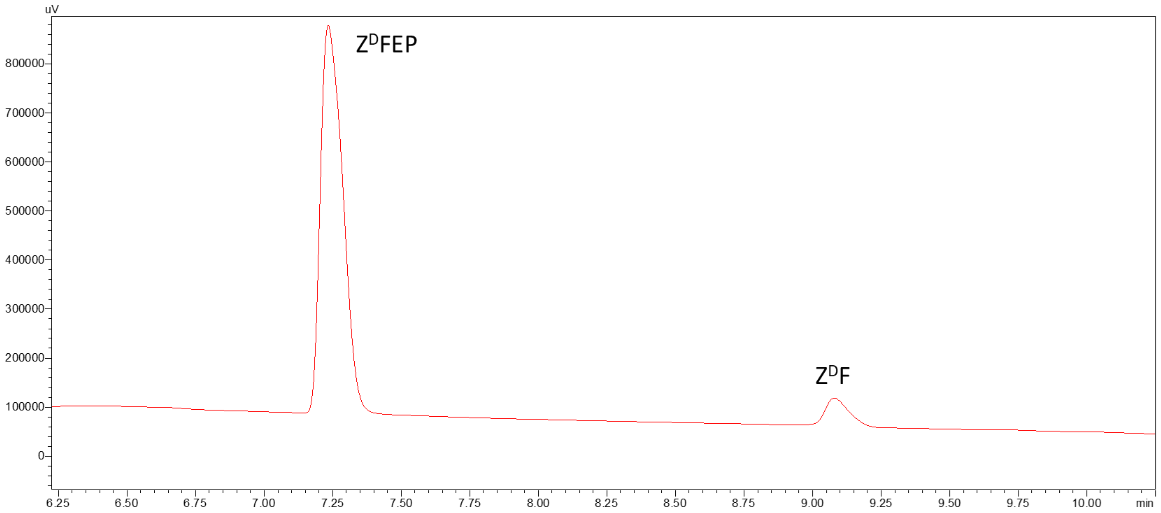

Figure S59: Chromatogram obtained from method 1 for Z^{D}FEP and its hydrolysis product Z^{D}F (7% peak area).

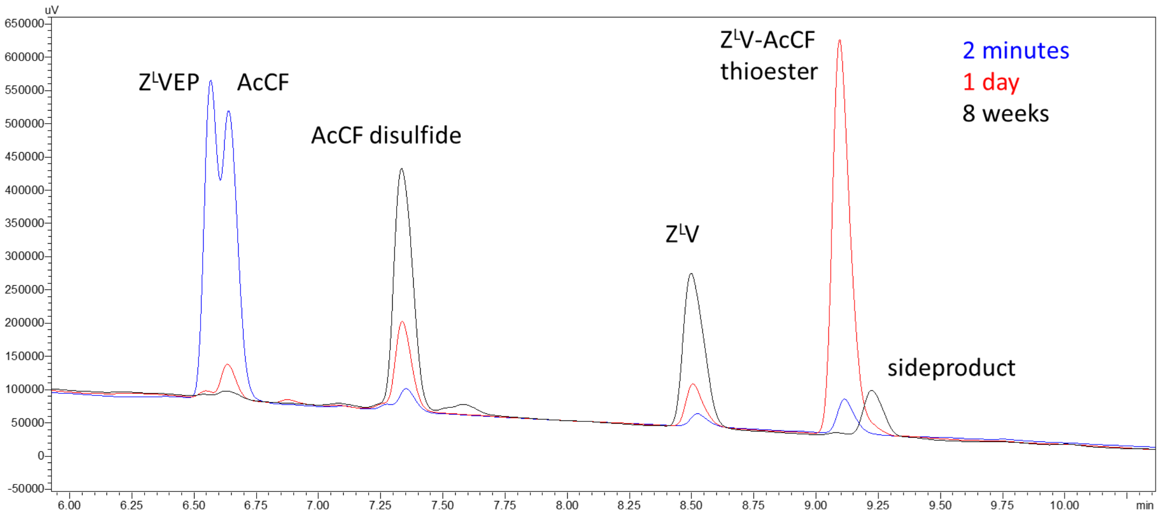

Figure S60: Overlaid chromatograms obtained from method 1 for the reaction of Z^{L}VEP (10 mM) with AcCF (10 mM) in borate buffer (0.6 M, pH 9.1) after 2 minutes (blue), 1 day (red) and 8 weeks (black). Side products were not characterized.

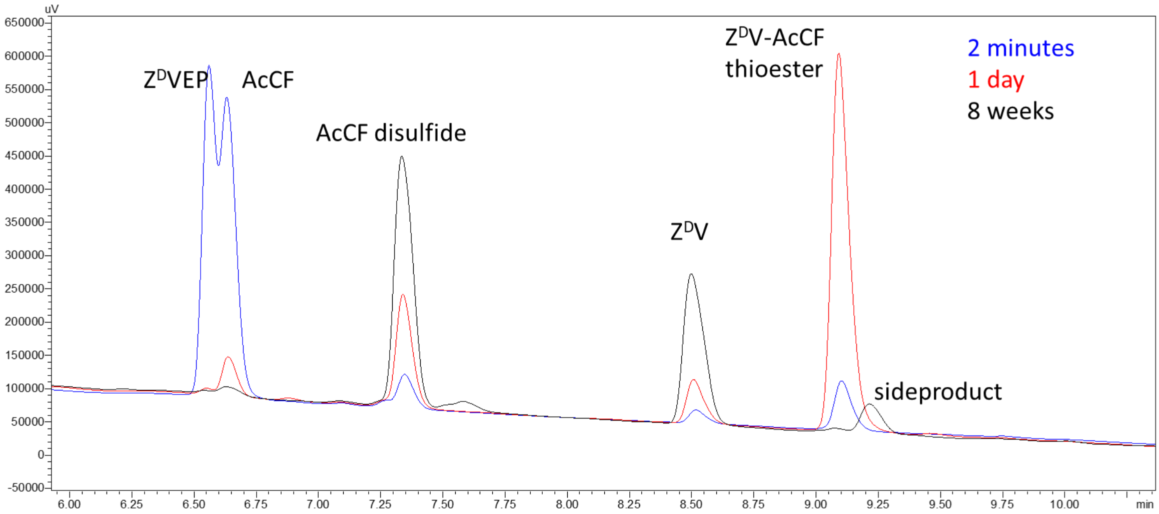

Figure S61: Overlaid chromatograms obtained from method 1 for the reaction of Z^{D}VEP (10 mM) with AcCF (10 mM) in borate buffer (0.6 M, pH 9.1) after 2 minutes (blue), 1 day (red) and 8 weeks (black). Side products were not characterized.

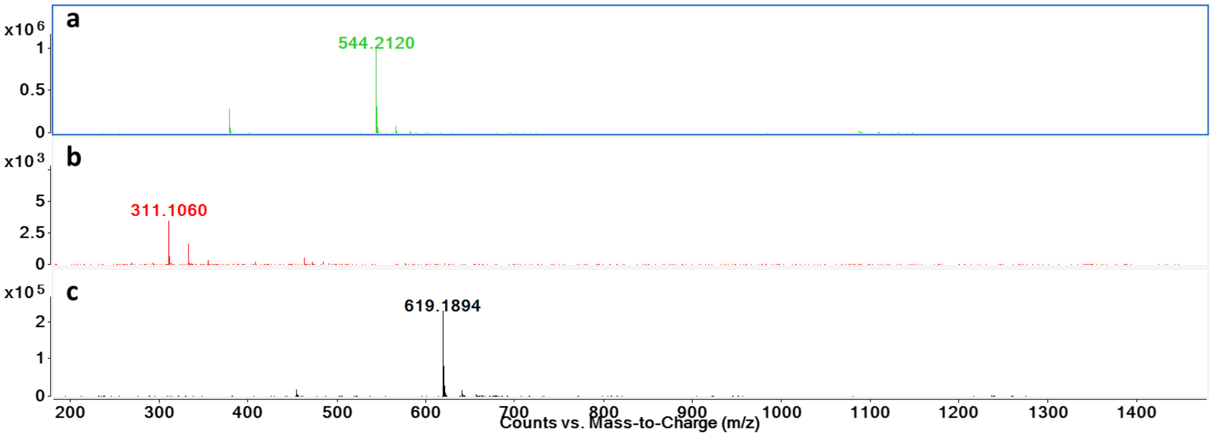

Figure S62: Mass spectra for the reaction of Z^{L}VEP (10 mM) with AcCF (10 mM) in borate buffer (0.6 M, pH 9.1). Retention times are similar for the D-driven system, and masses are identical. a) Z^{L}V-AcCF ester (retention time 9.09 min, m/z [M+H]$^+$ calculated: 544.2112, found: 544.2120), b) AcCF (retention time 6.63 min, m/z [M+H]$^+$ calculated: 311.1061, found: 311.1060) and c) AcCF disulfide (retention time 7.34 min, m/z [M+H]$^+$ calculated: 619.1891, found: 619.1894).

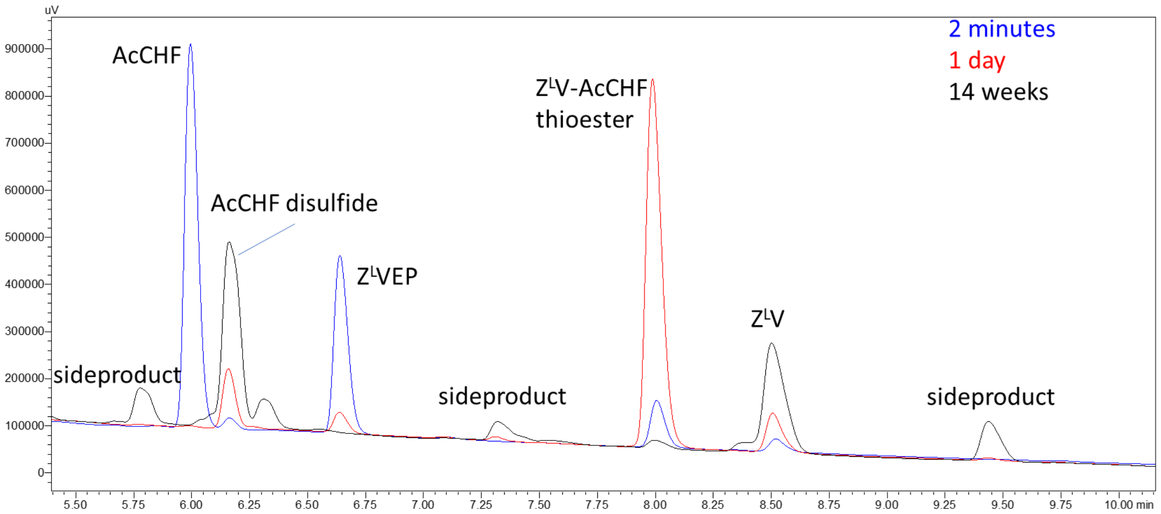

Figure S63: Overlaid chromatograms obtained from method 1 for the reaction of Z^{L}VEP (10 mM) with AcCHF (10 mM) in borate buffer (0.6 M, pH 9.1) after 2 minutes (blue), 1 day (red) and 14 weeks (black). Side products were not characterized.

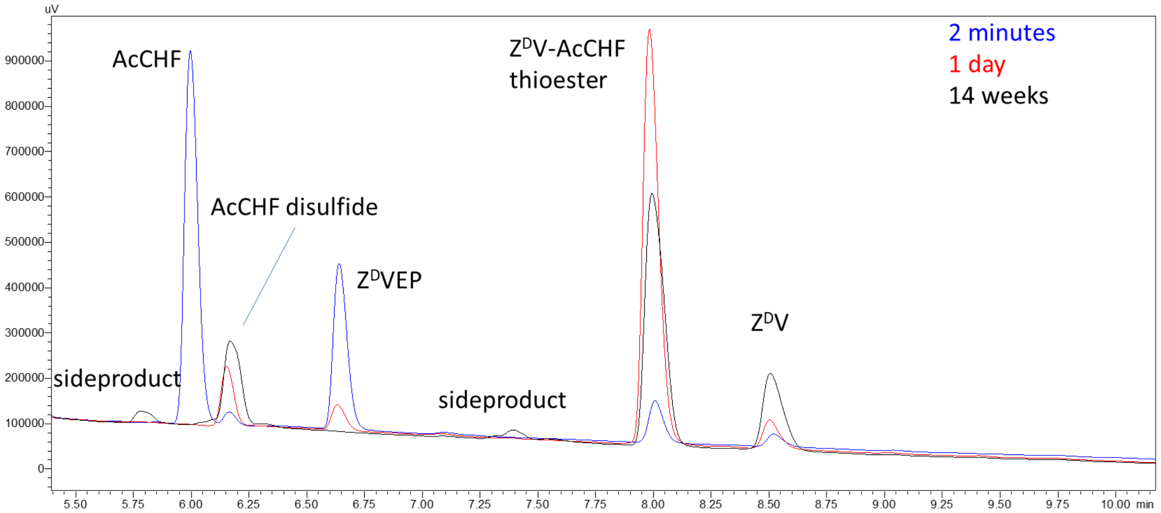

Figure S64: Overlaid chromatograms obtained from method 1 for the reaction of Z^{D}VEP (10 mM) with AcCHF (10 mM) in borate buffer (0.6 M, pH 9.1) after 2 minutes (blue), 1 day (red) and 14 weeks (black). Side products were not characterized.

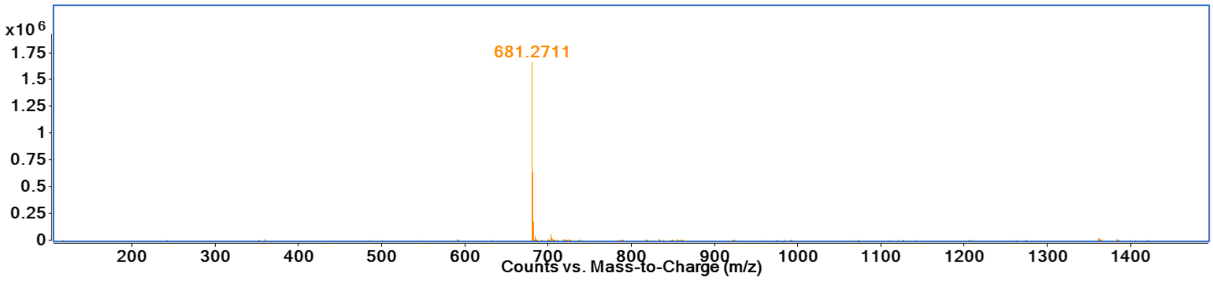

Figure S65: Mass spectra for the reaction of Z^{L}VEP (10 mM) with AcCHF (10 mM) in borate buffer (0.6 M, pH 9.1). Retention times are similar for the D-driven system, and masses are identical. Mass analysis for AcCHF and AcCHF disulfide can be found: in Figure S72. Z^{L}V-AcCHF ester: retention time 7.93 min, m/z $[M+H]^+$ calculated: 681.2702, found: 681.2711.

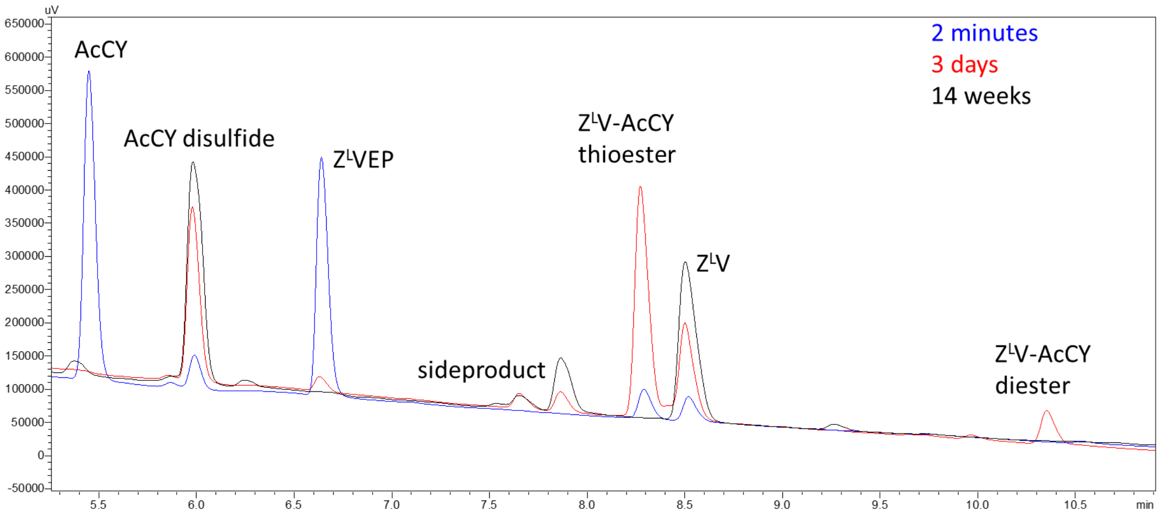

Figure S66: Overlaid chromatograms obtained from method 1 for the reaction of Z^{L}VEP (10 mM) with AcCY (10 mM) in borate buffer (0.6 M, pH 9.1) after 2 minutes (blue), 3 days (red) and 14 weeks (black). Side products were not characterized.

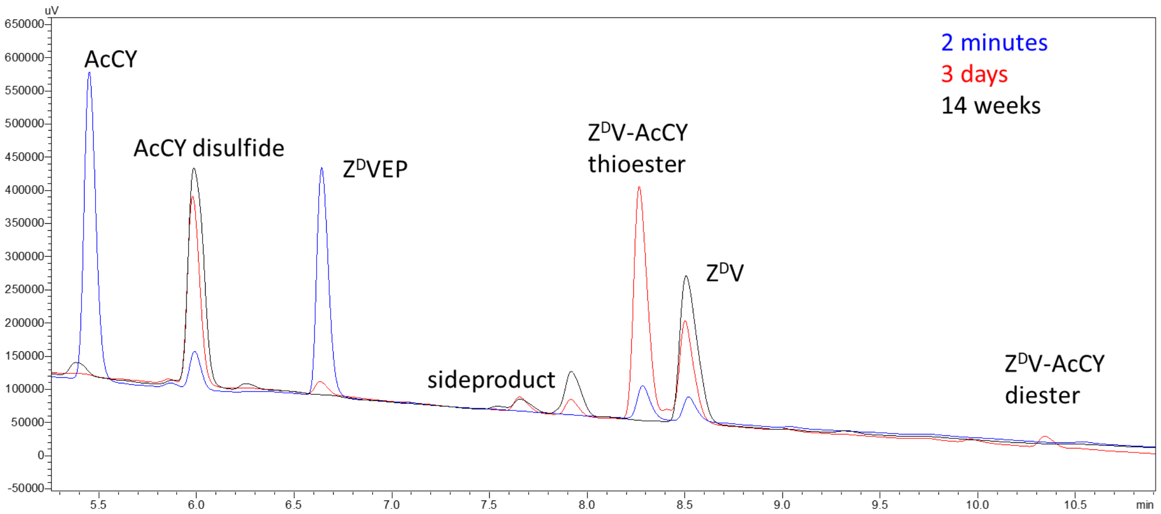

Figure S67: Overlaid chromatograms obtained from method 1 for the reaction of Z^{D}VEP (10 mM) with AcCY (10 mM) in borate buffer (0.6 M, pH 9.1) after 2 minutes (blue), 3 days (red) and 14 weeks (black). Side products were not characterized.

LC-MS analysis for the reaction of Z^{L}VEP (10 mM) with AcCY (10 mM) in borate buffer (0.6 M, pH 9.1) can be found in our previous publication.[2] Differences in yield for the diester compared to our previous study arise from two reasons: The pH was slightly lower in this study (8.53 after 4 weeks vs 8.74 following the procedure of the previous study) because the acylating agent was dissolved in 0.1 M MES pH 5.5 buffer first before addition of the pH-controlled peptide solution instead of directly adding the pH-controlled peptide solution to a powder of the acylating agent. Additionally, the sample preparation previously included vortexing and sonication of the reaction before taking out the UPLC-sample while the samples in this study were not submitted to mechanical stress during the reaction.

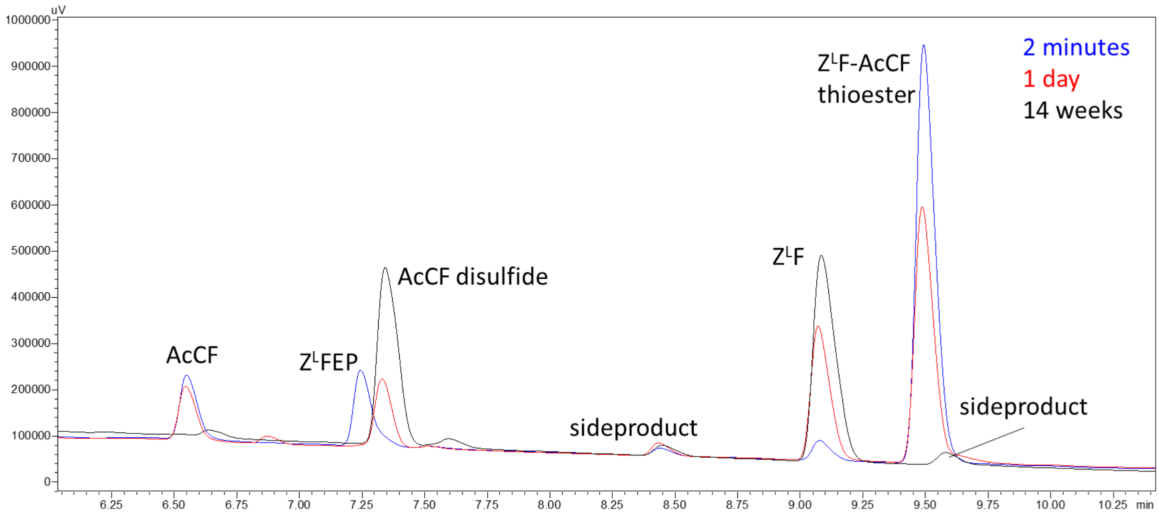

Figure S68: Overlaid chromatograms obtained from method 1 for the reaction of Z^LFEP (10 mM) with AcCF (10 mM) in borate buffer (0.6 M, pH 9.1) after 2 minutes (blue), 1 day (red) and 14 weeks (black). Side products were not characterized.

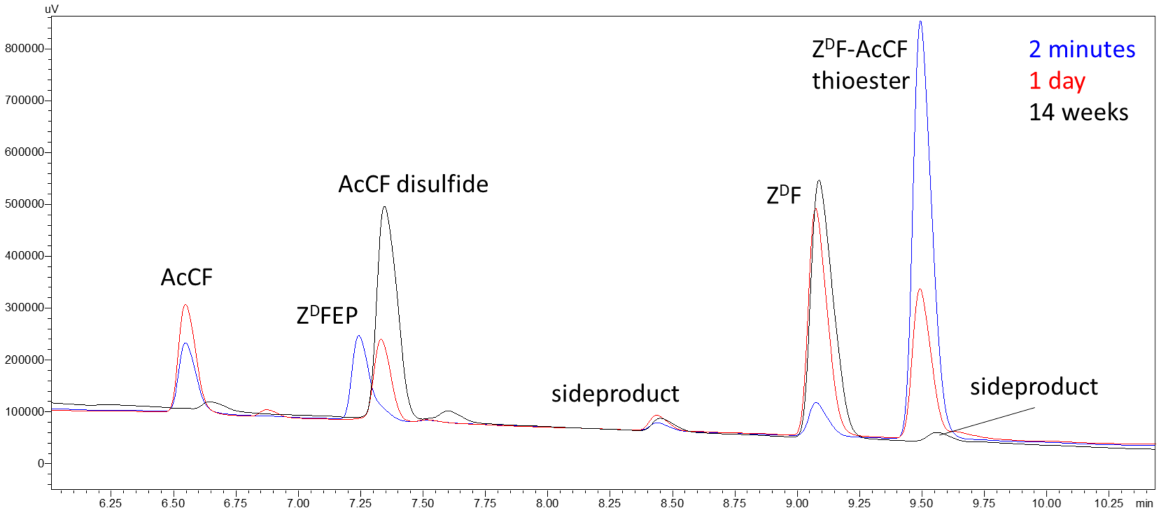

Figure S69: Overlaid chromatograms obtained from method 1 for the reaction of Z^DFEP (10 mM) with AcCF (10 mM) in borate buffer (0.6 M, pH 9.1) after 2 minutes (blue), 1 day (red) and 14 weeks (black). Side products were not characterized.

LC-MS analysis for the reaction of Z^LFEP (10 mM) with AcCF (10 mM) in borate buffer (0.6 M, pH 9.1) can be found in our previous publication.[4]

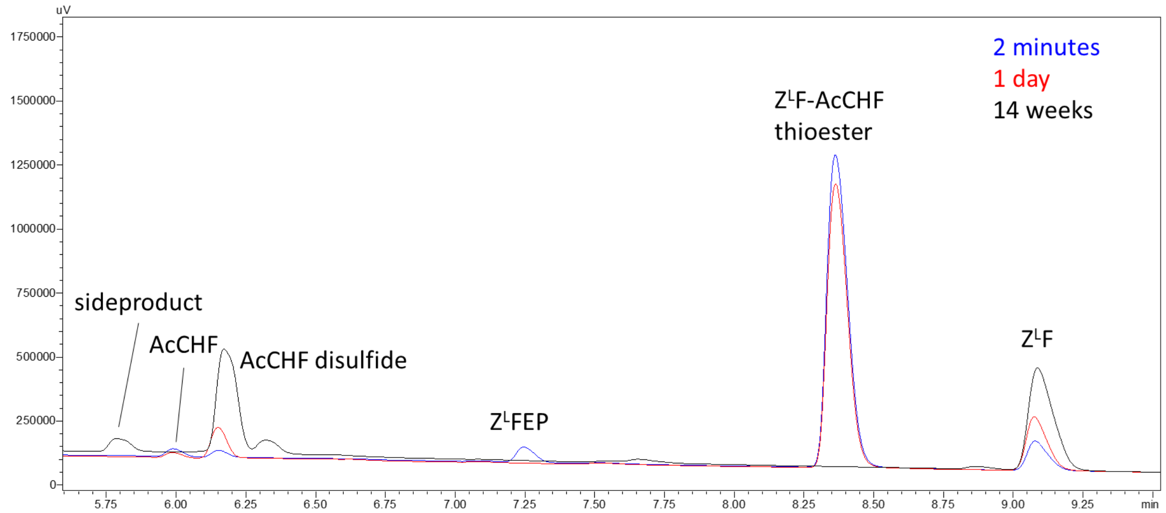

Figure S70: Overlaid chromatograms obtained from method 1 for the reaction of Z^{L}FEP (10 mM) with AcCHF (10 mM) in borate buffer (0.6 M, pH 9.1) after 2 minutes (blue), 1 day (red) and 14 weeks (black). Side products were not characterized.

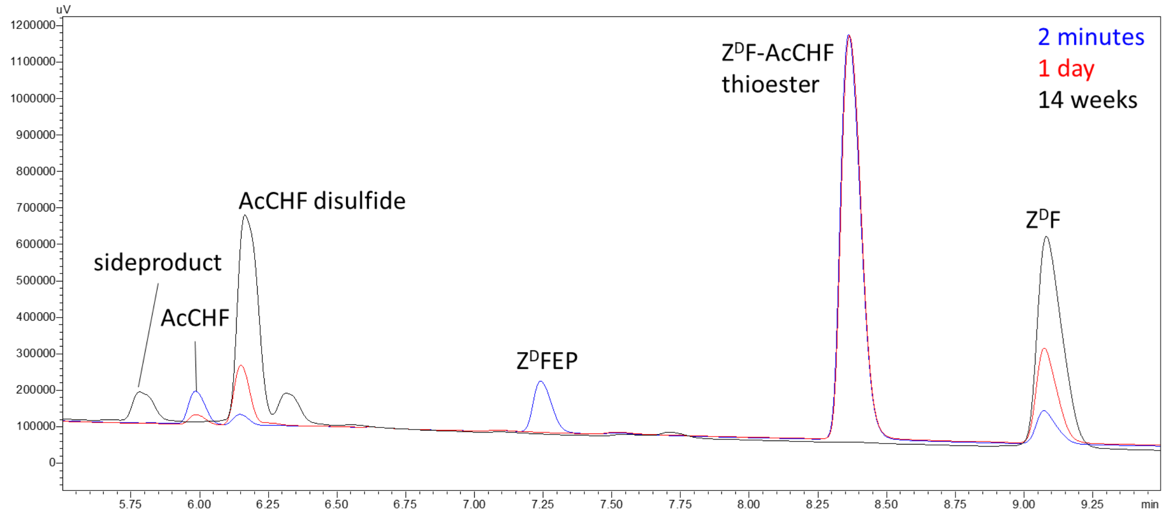

Figure S71: Overlaid chromatograms obtained from method 1 for the reaction of Z^{D}FEP (10 mM) with AcCHF (10 mM) in borate buffer (0.6 M, pH 9.1) after 2 minutes (blue), 1 day (red) and 14 weeks (black). Side products were not characterized.

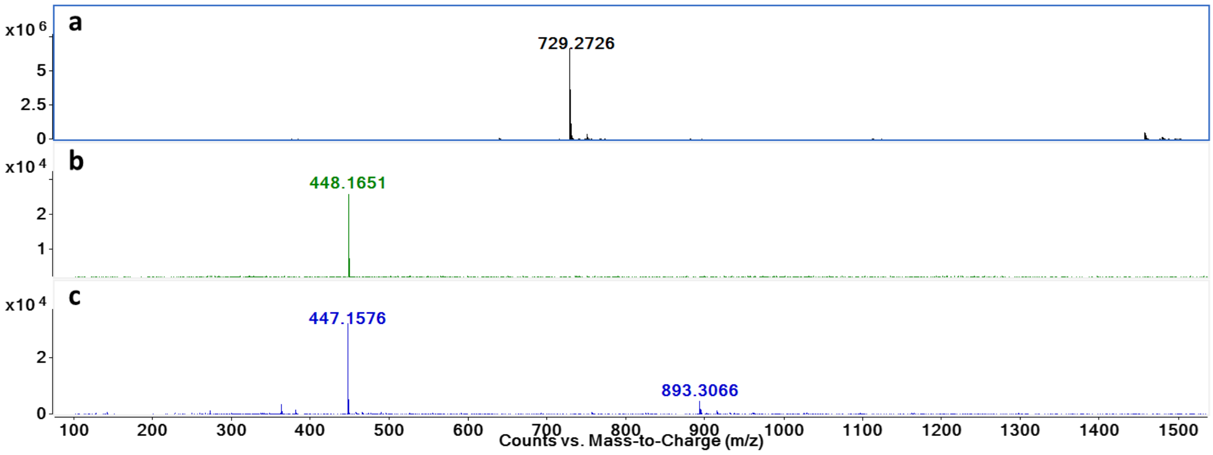

Figure S72: Mass spectra for the reaction of Z^{L}FEP (10 mM) with AcCHF (10 mM) in borate buffer (0.6 M, pH 9.1). Retention times are similar for the D-driven system, and masses are identical. a) Z^{L}F-AcCHF ester (retention time 8.38 min, m/z $[M+H]^+$ calculated: 729.2702, found: 729.2762), b) AcCHF (retention time 5.98 min, m/z $[M+H]^+$ calculated: 448.1650, found: 448.1651) and c) AcCHF disulfide (retention time 6.18 min, m/z $[M+H]^+$ calculated: 893.3070, found: 893.3066).

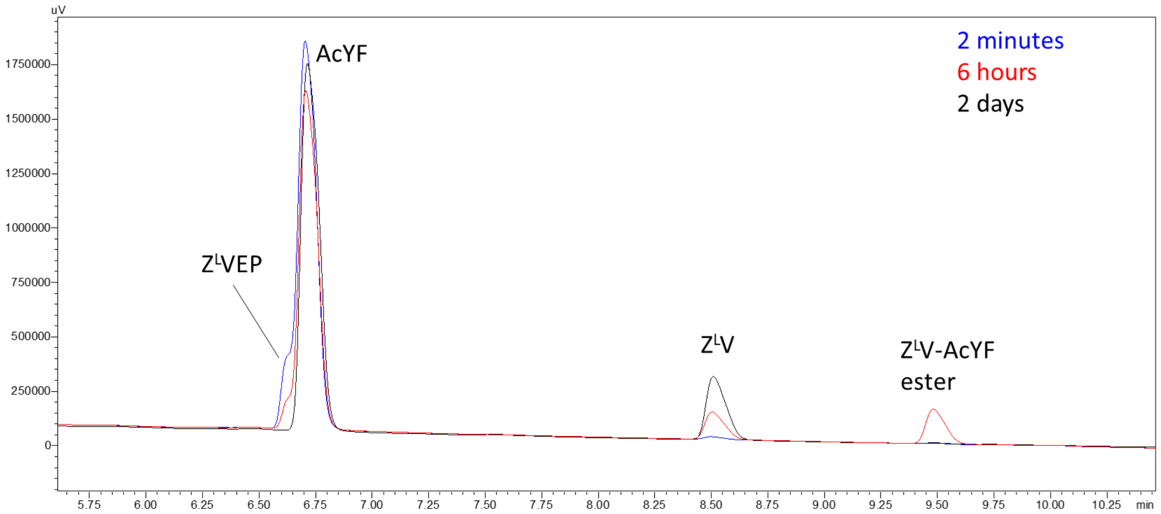

Figure S73: Overlaid chromatograms obtained from method 1 for the reaction of Z^{L}VEP (10 mM) with AcYF (20 mM) in bicarbonate buffer (0.2 M, pH 10.1) after 2 minutes (blue), 6 hours (red) and 2 days (black).

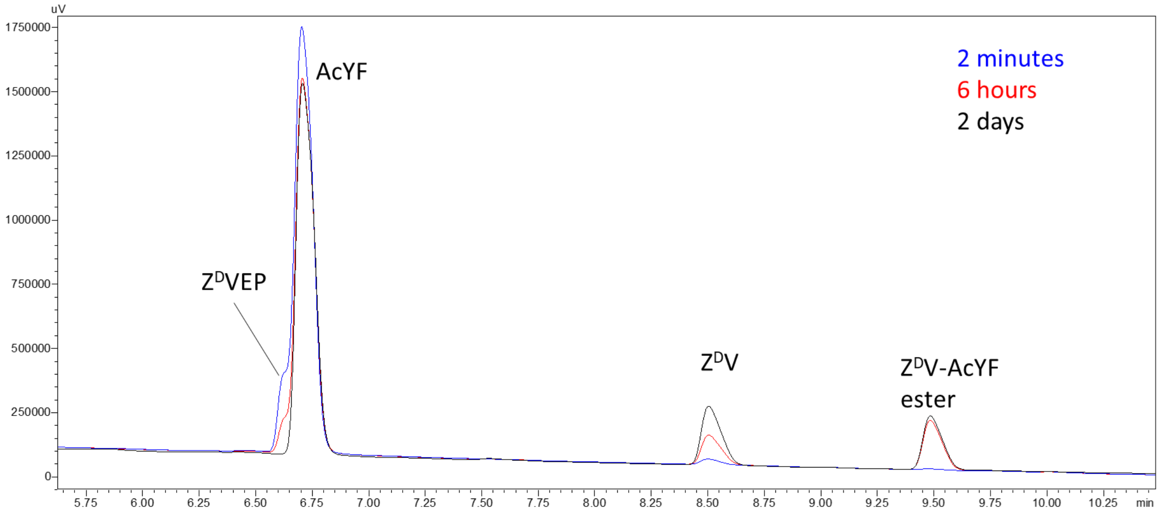

Figure S74: Overlaid chromatograms obtained from method 1 for the reaction of Z^{D}VEP (10 mM) with AcYF (20 mM) in bicarbonate buffer (0.2 M, pH 10.1) after 2 minutes (blue), 6 hours (red) and 2 days (black).

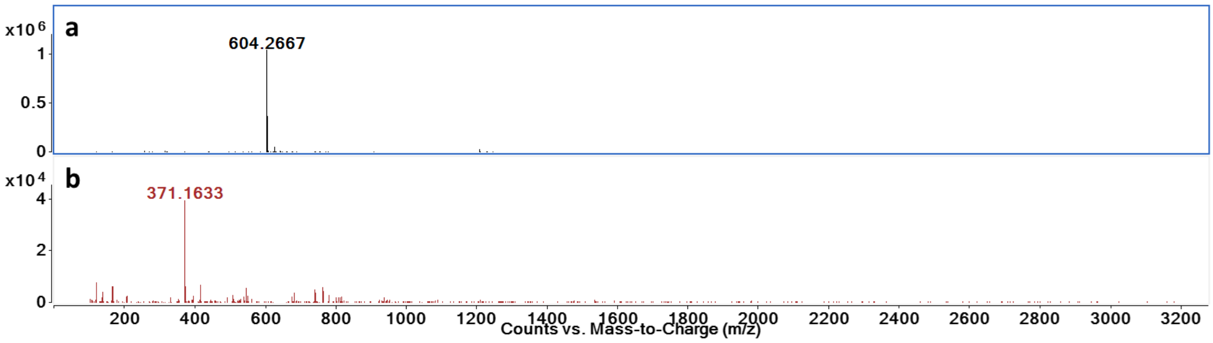

Figure S75: Mass spectra for the reaction of Z^{L}VEP (10 mM) with AcYF (20 mM) in bicarbonate buffer (0.2 M, pH 10.1). Retention times are similar for the D-driven system, and masses are identical. a) Z^{L}V-AcYF ester (retention time 9.48 min, m/z $[M+H]^+$ calculated: 604.2654, found: 604.2667) and b) AcYF (retention time 6.68 min, m/z $[M+H]^+$ calculated: 371.1602, found: 371.1633).

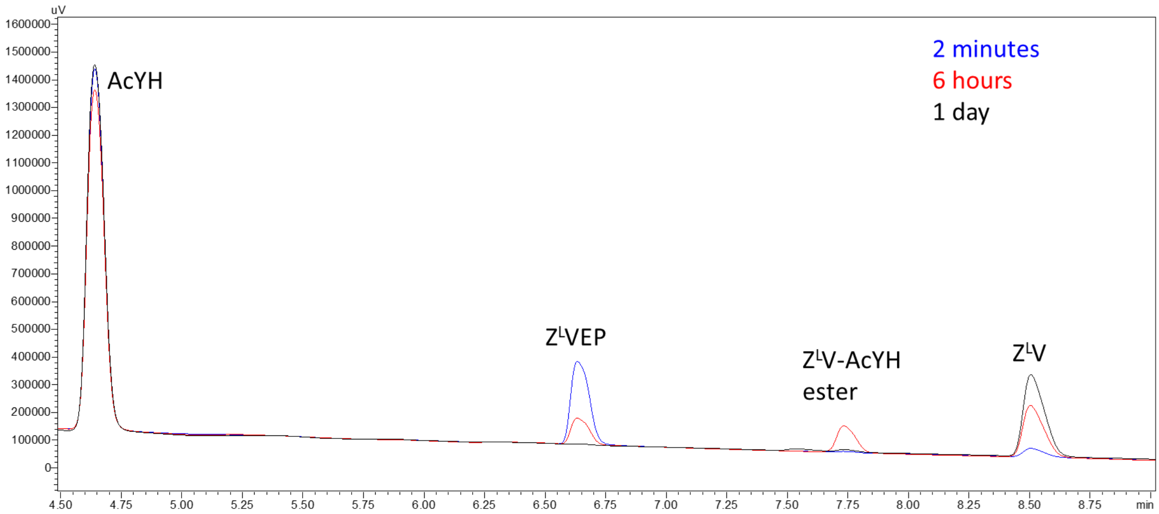

Figure S76: Overlaid chromatograms obtained from method 1 for the reaction of Z^{L}VEP (10 mM) with AcYH (20 mM) in bicarbonate buffer (0.2 M, pH 10.1) after 2 minutes (blue), 6 hours (red) and 1 day (black).

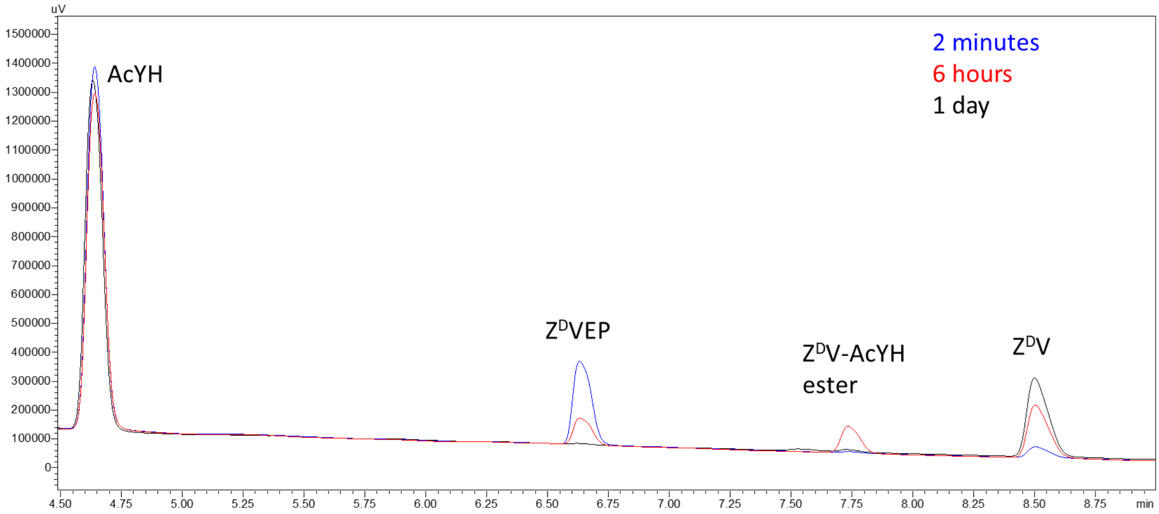

Figure S77: Overlaid chromatograms obtained from method 1 for the reaction of Z^{D}VEP (10 mM) with AcYH (20 mM) in bicarbonate buffer (0.2 M, pH 10.1) after 2 minutes (blue), 6 hours (red) and 1 day (black).

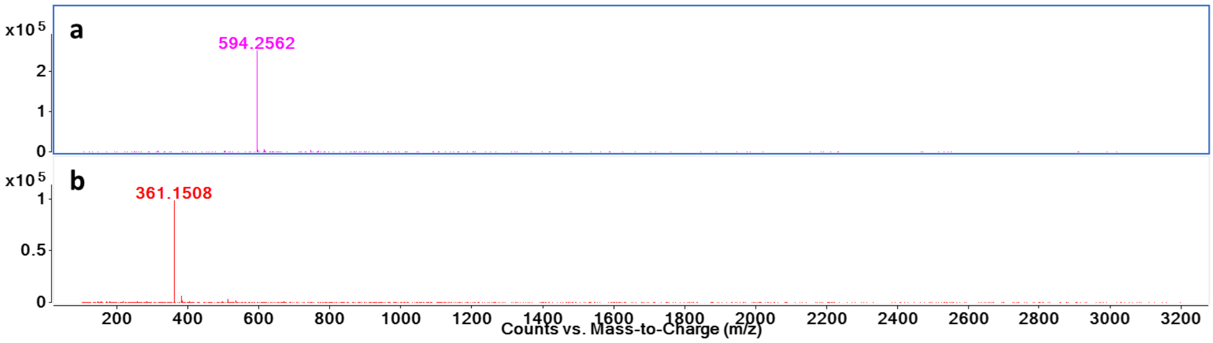

Figure S78: Mass spectra for the reaction of Z^{L}VEP (10 mM) with AcYH (20 mM) in bicarbonate buffer (0.2 M, pH 10.1). Retention times are similar for the D-driven system, and masses are identical. a) Z^{L}V-AcYH ester (retention time 7.73min, m/z $[M+H]^+$ calculated: 594.2559, found: 594.2562) and b) AcYH (retention time 4.62 min, m/z $[M+H]^+$ calculated: 361.1507, found: 361.1508).

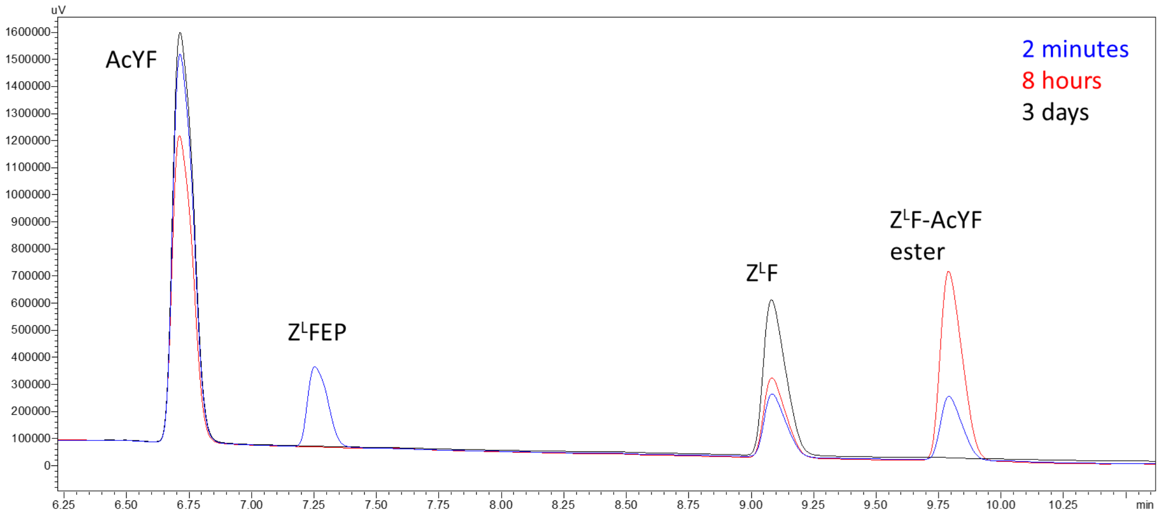

Figure S79: Overlaid chromatograms obtained from method 1 for the reaction of Z^{L}FEP (10 mM) with AcYF (20 mM) in bicarbonate buffer (0.2 M, pH 10.1) after 2 minutes (blue), 8 hours (red) and 3 days (black).

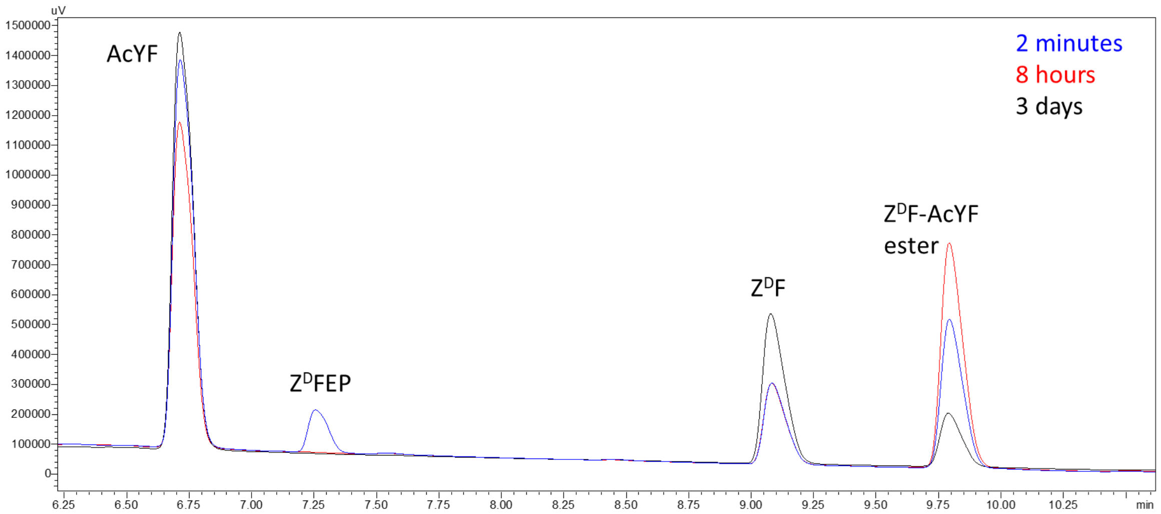

Figure S80: Overlaid chromatograms obtained from method 1 for the reaction of Z^{D}FEP (10 mM) with AcYF (20 mM) in bicarbonate buffer (0.2 M, pH 10.1) after 2 minutes (blue), 8 hours (red) and 3 days (black).

LC-MS analysis for the reaction of Z^{L}FEP (10 mM) with AcYF (20 mM) in bicarbonate buffer (0.2 M, pH 10.1) can be found in our previous publication.[4]

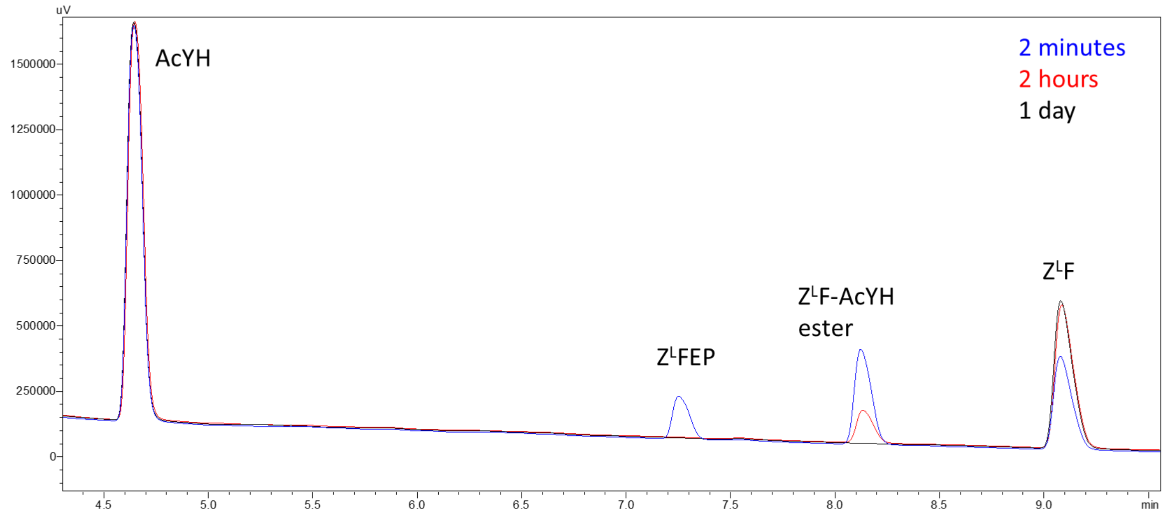

Figure S81: Overlaid chromatograms obtained from method 1 for the reaction of Z^LFEP (10 mM) with AcYH (20 mM) in bicarbonate buffer (0.2 M, pH 10.1) after 2 minutes (blue), 2 hours (red) and 1 day (black).

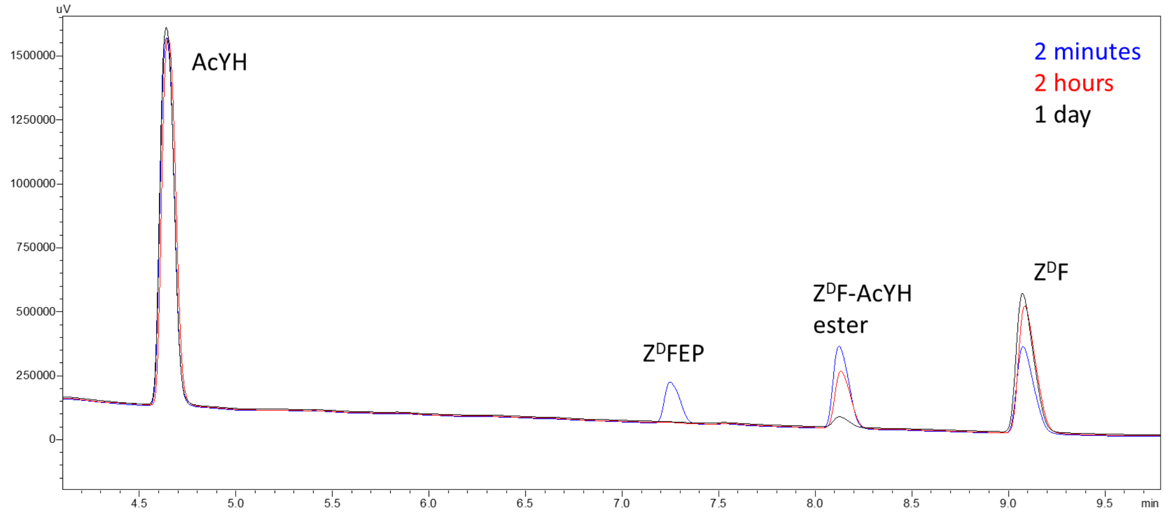

Figure S82: Overlaid chromatograms obtained from method 1 for the reaction of Z^DFEP (10 mM) with AcYH (20 mM) in bicarbonate buffer (0.2 M, pH 10.1) after 2 minutes (blue), 2 hours (red) and 1 day (black).

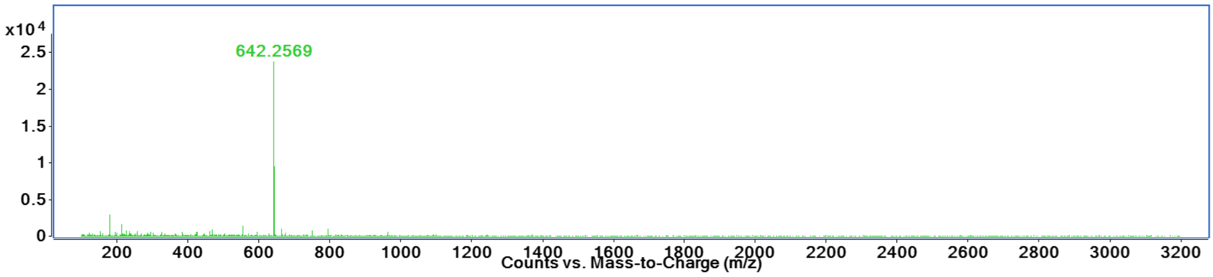

Figure S83: Mass spectra for the reaction of Z^LFEP (10 mM) with AcYH (20 mM) in bicarbonate buffer (0.2 M, pH 10.1). Retention times are similar for the D-driven system, and masses are identical. Z^LF-AcYH ester (retention time 8.12 min, m/z $[M+H]^+$ calculated: 642.2559, found: 642.2569)

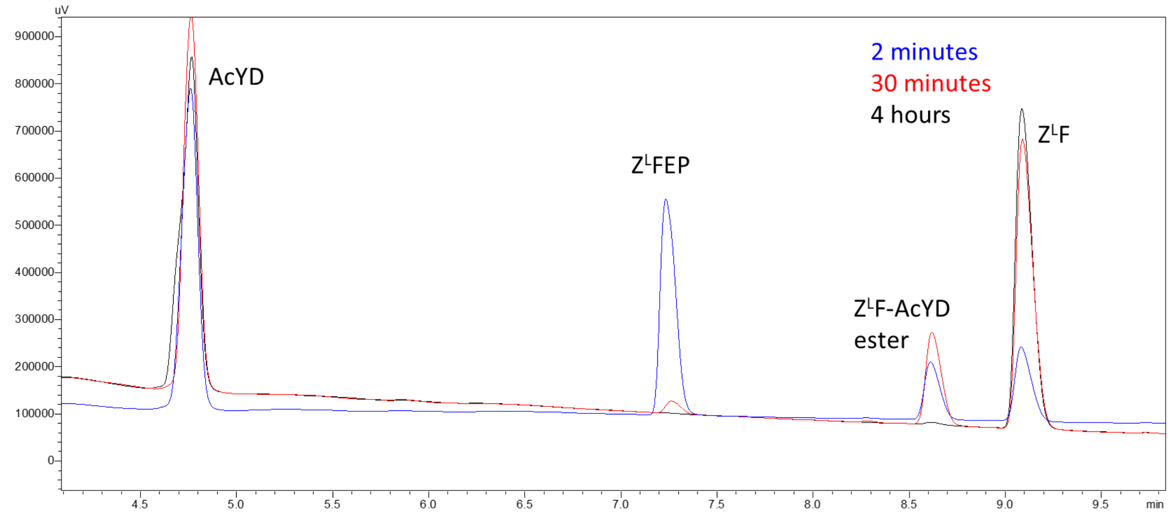

Figure S84: Overlaid chromatograms obtained from method 1 for the reaction of Z^LFEP (10 mM) with AcYD (20 mM) in bicarbonate buffer (0.2 M, pH 10.1) after 2 minutes (blue), 30 minutes (red) and 4 hours (black).

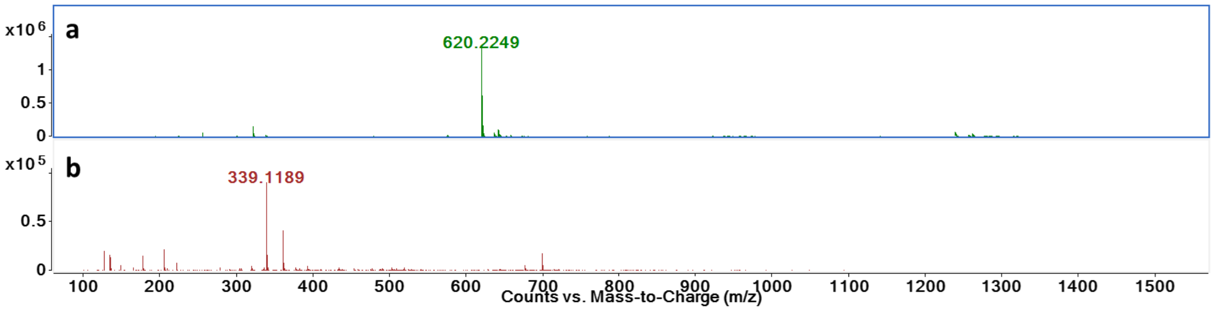

Figure S85: Mass spectra for the reaction of Z^LFEP (10 mM) with AcYD (20 mM) in bicarbonate buffer (0.2 M, pH 10.1). Retention times are similar for the D-driven system, and masses are identical. a) Z^LF-AcYD ester (retention time 8.62 min, m/z $[M+H]^+$ calculated: 620.2239, found: 620.2249) and b) AcYD (retention time 4.74 min, m/z $[M+H]^+$ calculated: 339.1187, found: 339.1189).

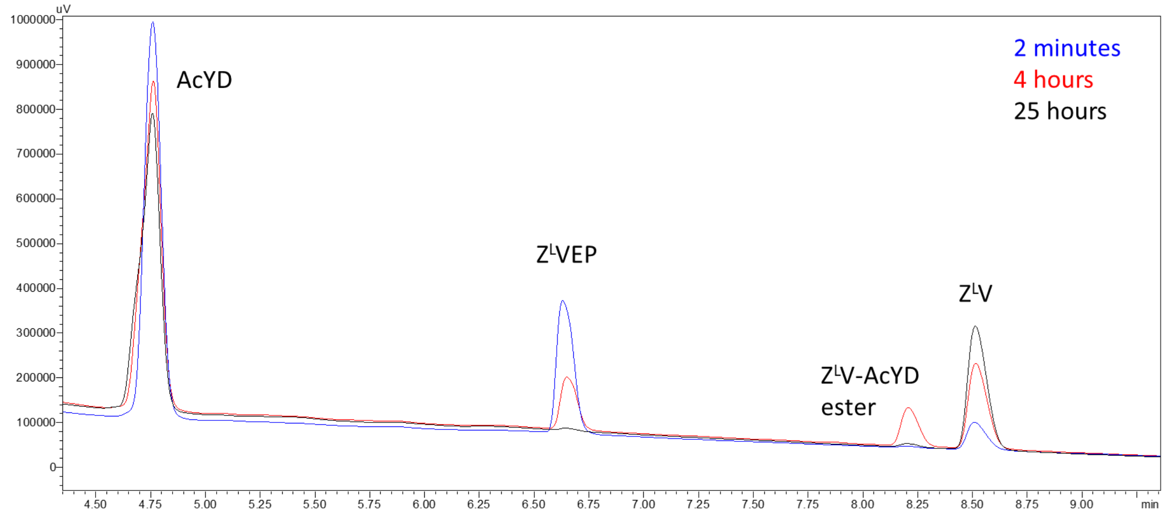

Figure S86: Overlaid chromatograms obtained from method 1 for the reaction of Z^LVEP (10 mM) with AcYD (20 mM) in bicarbonate buffer (0.2 M, pH 10.1) after 2 minutes (blue), 4 hours (red) and 25 hours (black).

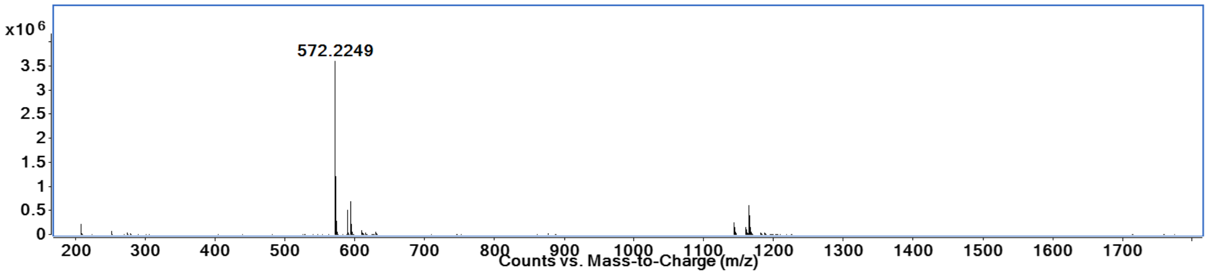

Figure S87: Mass spectra for the reaction of $Z^{L}VEP$ (10 mM) with AcYD (20 mM) in bicarbonate buffer (0.2 M, pH 10.1). Retention times are similar for the D-driven system, and masses are identical. $Z^{L}V$-AcYD ester (retention time 8.19 min, m/z $[M+H]^{+}$ calculated: 572.2239, found: 572.2249)

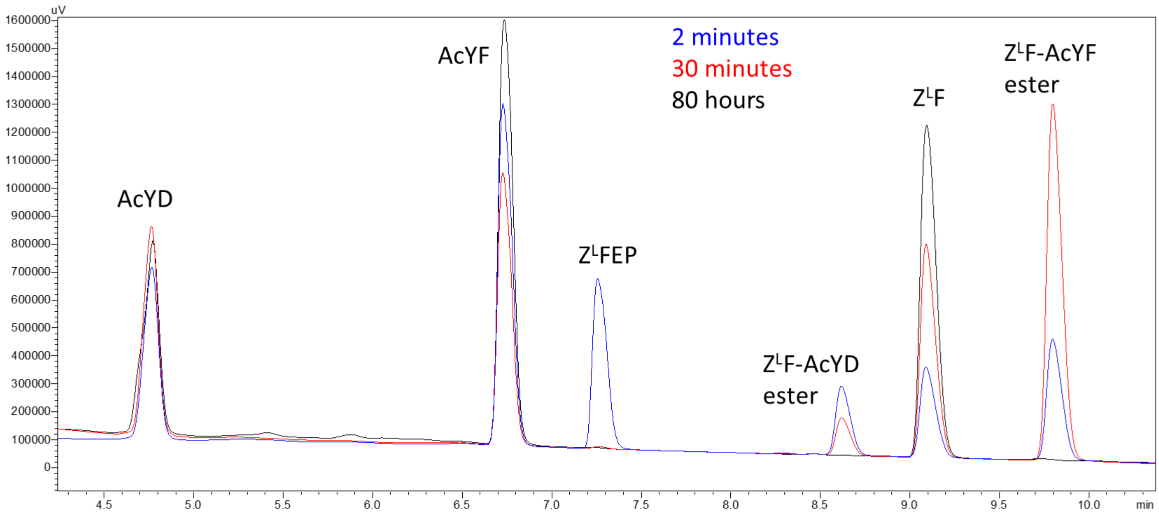

Figure S88: Overlaid chromatograms obtained from method 1 for the reaction of Z^{L}FEP (20 mM) with AcYD (20 mM) and AcYF (20 mM) in bicarbonate buffer (0.2 M, pH 10.1) after 2 minutes (blue), 30 minutes (red) and 80 hours (black).

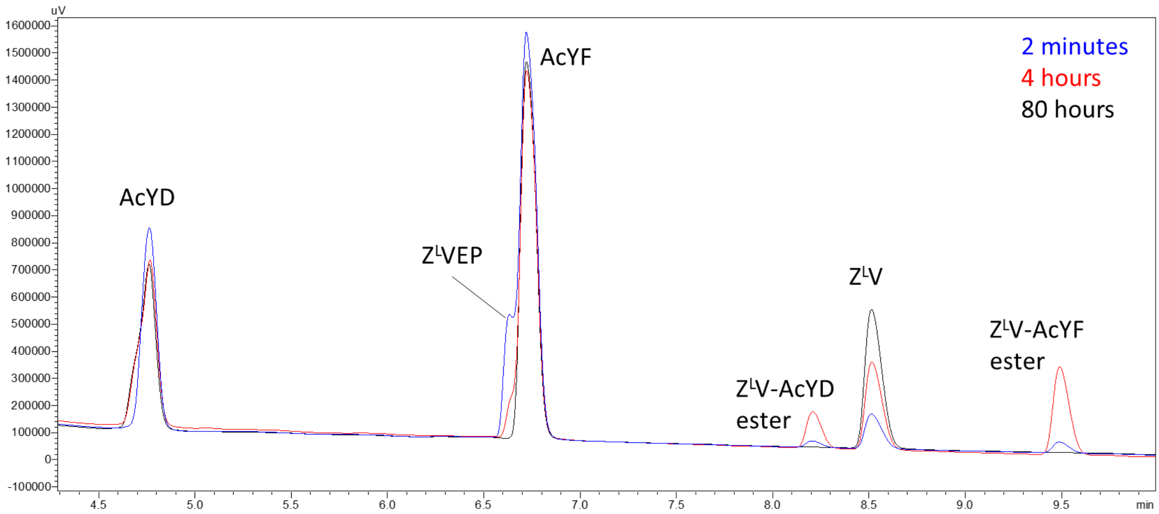

Figure S89: Overlaid chromatograms obtained from method 1 for the reaction of Z^{L}VEP (20 mM) with AcYD (20 mM) and AcYF (20 mM) in bicarbonate buffer (0.2 M, pH 10.1) after 2 minutes (blue), 4 hours (red) and 80 hours (black).

6.9 References

[1] A. Englert, F. Majer, J. L. Schiessl, A. J. C. Kuehne, M. Von Delius, *Chem* **2024**, *10*, 910–923.

[2] M.D. Pol, R. Thomann, Y. Thomann, C.G. Pappas, *J. Am. Chem. Soc.* **2024**, *146*, 29621–29629.

[3] S. Hoops, S. Sahle, R. Gauges, C. Lee, J. Pahle, N. Simus, M. Singhal, L. Xu, P. Mendes, U. Kummer, *Bioinformatics* **2006**, *22*, 3067–3074.

[4] M. D. Pol, K. Dai, R. Thomann, S. Moser, S. Kanti Roy, C. G. Pappas, *Angew. Chem. Int. Ed.* **2024**, *63*, e202404360.
